# Benchmarking antigen-aware inverse folding methods for antibody design

**DOI:** 10.1093/bioadv/vbag081

**Published:** 2026-03-20

**Authors:** Bartosz Janusz, Dawid Chomicz, Sonia Wrobel, Pawel Dudzic, Adithya Polasa, Kyle Martin, Steven Darnell, Stephen R Comeau, Konrad Krawczyk

**Affiliations:** NaturalAntibody, Szczecin, Poland; NaturalAntibody, Szczecin, Poland; NaturalAntibody, Szczecin, Poland; NaturalAntibody, Szczecin, Poland; Boehringer-Ingelheim, Ridgefield, CT, United States; Boehringer-Ingelheim, Ridgefield, CT, United States; Boehringer-Ingelheim, Ridgefield, CT, United States; Boehringer-Ingelheim, Ridgefield, CT, United States; NaturalAntibody, Szczecin, Poland

## Abstract

Computational antibody design has seen many recent advances pioneered via the use of language models and advanced structure prediction tools. Developing a de novo antibody against a specific antigen requires structural awareness that most language models lack. A prominent class of machine learning methods combining the best of language model and structural worlds is inverse folding. This approach aims to predict a sequence that would fit a given structure. Such methods are now increasingly used to predict alternate sequences given a structure of a binder. It is known that, just like language models, such methods have certain predictive power in identifying binders. Here we performed a set of tests to reveal where, if at all, such methods provide value in the realistic setting of antibody discovery.

## Introduction

Antibodies play a critical role in the immune system and have become essential in therapeutics and diagnostics due to their highly specific antigen recognition capabilities. Engineering antibodies to enhance properties such as affinity, specificity, and stability has thus become an active research area, increasingly powered by machine learning and computational approaches (Bielska *et al.* 2025). Among these, inverse folding methods present a promising paradigm for antibody design (Li *et al.* 2024). Unlike traditional design approaches, which aim to determine a protein’s 3D structure based on a given sequence, inverse folding begins with a target structure and searches for sequences that best fold into that structure. This shift in focus has been particularly invigorated by advancements in structural prediction models like AlphaFold, which generates millions of predicted protein structures, fueling the training of inverse folding models (Varadi *et al.* 2022).

In the realm of antibody design, inverse folding methods offer distinct advantages by focusing on maintaining the native fold of an antibody structure, crucial for preserving its antigen-binding capabilities. Recent advancements in protein-generic inverse folding algorithms, such as ProteinMPNN (Dauparas *et al.* 2022) and ESM-IF (Shanker *et al.* 2024), have achieved high sequence recovery and structural fidelity, establishing a foundation for antibody-specific inverse folding models. For instance, AbMPNN (Dreyer *et al.* 2023) and AntiFold (Høie *et al.* 2025) have fine-tuned these foundational methods using antibody-focused datasets, yielding notable improvements in sequence recovery, structural accuracy, and binding affinity predictions across antibody complementarity-determining regions (CDRs).

Despite these innovations, evaluating the effectiveness of inverse folding methods for antibody design poses unique challenges. Current evaluation metrics for generative models often rely on in silico criteria, which are split into sequence-based metrics, such as amino acid recovery (AAR), and structure-based metrics, such as root-mean-square deviation (RMSD) from target structures. Previous study into benchmarking antibody inverse folding methods chiefly focused on such in-silico metrics (Li *et al.* 2024). Using public datasets, this study revealed important shortcomings of these methods, such as weak correlations with affinity and limited usage of antigen information, in contradiction to previous findings (Shanker *et al.* 2024). Such metrics, though useful, might not be directly transferable to antibody discovery campaigns functional performance. Furthermore, structure-based confidence scores like predicted Local Distance Difference Test (pLDDT) and interface-predicted Template Modeling (ipTM), while valuable for structural validation, are inadequate proxies for binding efficacy in antibodies. Physics-based metrics, though theoretically promising due to their consideration of biophysical properties, face high computational demands and have shown weak correlations with experimental affinity measurements (Hummer *et al.* 2023).

In this work, we address these limitations by benchmarking state-of-the-art inverse folding models specifically for antibody design. We perform evaluation on three diverse experimental datasets, focusing on realistic design applications, including a proper hold out in the form of our structure-informed AbDesign dataset (Janusz *et al.* 2025). This benchmark confirms that ML methods such as inverse folding can be used to enrich binders from a large pool of potential ones. Nonetheless, our results show that the methods make very limited use of the antigen, which leaves a clear direction for improvement.

## Methods

### AbDesign DB dataset

The AbDesign DB dataset was generated by selecting seven antigens, with each one being recognized by two distinct antibodies. Some of these targets were therapeutically significant, like VEGF and PD-1, while others were common antigens that are frequently crystallized in the PDB. The CDR-H3 regions had a median length of 12 residues. However, our primary focus was not specifically on therapeutic antibodies or relevant targets.

We identified interactions between the CDR-H3 regions and antigen residues, defining these contacts as cases where heavy atoms were within a distance of 4.5 Å. These contact positions were subsequently chosen for point mutations. The aim was to maximize the number of single mutants and distinct mutation sites while keeping the total number of variants capped at 96 antibodies per plate per target. To achieve this, mutations were distributed automatically to ensure a wide range of positions were covered, avoiding a bias towards any specific site. Each mutation set was then manually reviewed to exclude non-informative or irrelevant mutations, such as the introduction of cysteines. This resulted in a total of 658 mutations, averaging 47 mutations per antibody.

We expect this dataset to be particularly useful in affinity maturation experiments, where mutations need to be introduced without affecting wild-type binding. To support this, we used structures modelled and refined for our AbDesign dataset (https://pubmed.ncbi.nlm.nih.gov/41058476/). Each mutated antibody sequence was modeled using ABodyBuilder2 (Abanades *et al.* 2023) and optimized with OpenMM. Wild-type structures were also refined using the same relaxation procedure. Each file was labeled with its specific mutation. The structural data can be cross-referenced with sequence information and ELISA ratios, making it suitable for enhanced machine learning analyses.

### Anti-PD1 dataset

The original dataset was composed of 59 unique heavy and light sequences of binding antibodies only. Additional 59 non-binder sequences were generated in the following manner: for each heavy binder sequence, replace part of CDR3 (leave 2 AAs at the beginning, 3 at the end) with a random AA sequence (excluding cysteine) matching the original length. The sequences have varying lengths (Supplementary Figure 1, available as supplementary data at *Bioinformatics* online).

### Trastuzumab HER2 large

The HER2-aff-large dataset consists of over half a million Trastuzumab variants categorized into three affinity classes—‘high,’ ‘medium,’ and ‘low’—based on their binding strength to HER2 (Chinery *et al.* 2024). These variants were derived by limiting mutations to the IMGT positions between 107 and 116 of the antibody’s heavy chain. The dataset is an extension of previous DMS (Deep Mutational Scanning) results from Mason *et al.* and includes 178,160 high-affinity, 196,392 medium-affinity, and 171,732 low-affinity binders.

A small percentage of overlap between affinity classes was observed, with 1.1% of sequences classified as both ‘high’ and ‘medium’ binders, and 2.9% found in both ‘high’ and ‘low’ categories. By addressing these overlaps, the dataset was refined to 530,357 sequences, resulting in a 33.6% class imbalance. Further refining by removing all overlapping sequences entirely reduced the dataset to 524,346 sequences with a class imbalance of 32.8%. CDRH3 residue distributions can be seen in Supplementary Figures 2 and 3, available as supplementary data at *Bioinformatics* online.

The dataset was used to analyze binding behaviors, with clustering analysis showing that the ‘medium’ and ‘low’ classes grouped with negative binders from the original Mason *et al.* study. Classification models predicted high binding probabilities for the ‘high’ affinity class and low probabilities for the ‘medium’ and ‘low’ affinity groups. For binary classification, the high-affinity sequences were labeled as positive binders, and the medium- and low-affinity sequences were grouped as negative binders, aligning with the goal of selecting high-affinity antibodies during the optimization process.

### Inverse folding methods benchmarked

Four inverse-folding models were selected in this study, two protein generic ones (ESM-IF and ProteinMPNN) and their two antibody-focused versions (AntiFold and AbMPNN).

The ESM-IF1 (Hsu *et al.* 2022) inverse folding model is a computational tool developed by Meta AI as part of the Evolutionary Scale Modeling (ESM) series, which focuses on protein structure prediction and analysis. ESM-IF1 is specifically designed for protein inverse folding, a task that involves predicting a protein sequence given its three-dimensional (3D) structure, rather than the more common task of predicting the structure from a sequence. It is based on a GVP network trained on millions of structures from the AlphaFold2 Database.

AntiFold (Høie *et al.* 2025) is fine-tuned from the ESM-IF1 model with improved sequence recovery in complementarity-determining regions (CDRs), which are crucial for antigen recognition. It significantly improves upon existing models in this area, achieving a mean root-mean-square deviation (RMSD) of 0.95 for CDR regions, compared to higher RMSD values from other models like AbMPNN and ProteinMPNN.

ProteinMPNN (Dauparas *et al.* 2022) is a deep learning-based method for protein sequence design in the Baker Lab. It is based on a message passing neural network, and it was trained on the crystal structures from the PDB rather than model structures, as was the case with ESM-IF.

The AbMPNN (Dreyer *et al.* 2023) model is a fine-tuned, antibody-specific graph neural network model based on ProteinMPNN, designed to predict and design antibody sequences. It focuses on the variable CDR loops, particularly CDR-H3, which is vital for antigen recognition. The model is trained on large-scale antibody datasets such as SAbDab and OAS, as well as predicted structures from ABodyBuilder2, to provide state-of-the-art performance in antibody sequence recovery and design.

### Perplexity definition

In all benchmarked models, perplexity is defined as the exponential of cross entropy loss, averaged over all (or subset) residues in a scored sequence.


$$\mathrm{MeanCrossEntropy}\;=l(p,\;y)\;=\;-\frac{\sum_{i\epsilon N}^{}y_{i}\;\text{log}(p_{i})}{i}$$

$$\mathrm{Perplexity}\;=\;e^{\mathrm{MeanCrossEntropy}}$$

Where *N* is sequence length.

While perplexity has inherent limitations as a direct proxy for binding free energy, it remains a uniquely practical and principled metric for comparative benchmarking across diverse model architectures. Perplexity provides a task-agnostic surrogate for a model’s confidence that a sequence is compatible with a given structural or sequence context, enabling fair cross-model comparison without requiring task-specific training or fine-tuning—the alternative would demand integrating explicit binding affinity prediction heads directly into each model’s architecture, substantially complicating deployment and limiting comparability across heterogeneous model families (sequence-only language models, structure-conditioned inverse folding models, etc.). Although perplexity reflects global sequence likelihood under the model’s training distribution and therefore conflates many constraints rather than isolating thermodynamic contributions at the interface, these same limitations are why it serves as a robust comparative lens: it captures each model’s implicit learning of biophysically-relevant intra-sequence/sequence-structure relationships without imposing external constraints. Dedicated interaction models and affinity-trained predictors will consistently outperform raw perplexity for absolute binding affinity prediction, but perplexity remains the most practical and principled metric for comparative enrichment analysis across models when such specialized architectures are unavailable or impractical to deploy uniformly.

## Results

### Overview of experiments

We tested four inverse folding models: ProteinMPNN, AbMPNN, ESM-IF, and AntiFold. ProteinMPNN and ESM-IF are state-of-the-art protein inverse folding models, whereas AbMPNN and ESM-IF are their antibody versions, respectively. The choice of the datasets reflected state-of-the-art & allowed us to test whether fine-tuning on antibodies brings any benefits in actual antibody discovery tasks beyond amino acid recovery, which is the objective task the models are trained on.

We performed tests on three datasets, HER2, BI-PD1, and NaturalAntibody AbDesign DB. The HER2 dataset is a public resource with 500,000 trastuzumab variants. We employed this dataset as a sanity check to see whether in our hands, we could get the inverse folding models to demonstrate their basic reported function—correlate with higher affinity binders. The BI-PD1 dataset was internally provided by Boehringer-Ingelheim (BI) with PD1 binders and non-binders. The experiment on HER2, which was supposed to separate higher from lower affinity binders, was supposed to be repeated on a real dataset used for antibody discovery. The final dataset, NaturalAntibody AbDesign DB, is a heterogeneous dataset of CDR-H3 mutants of existing structures. This was the final experimental test on how the methods fare in seemingly easy tasks of suggesting binding CDR-H3 mutations.

### Benchmarking enrichment of Trastuzumab variants against HER2

We benchmarked four inverse folding models—ESM-IF, Antifold (ESM-IF fine-tuned on antibodies), ProteinMPNN, and AbMPNN (ProteinMPNN fine-tuned on antibodies)—using the HER2-aff-large dataset containing over 500,000 CDRH3 sequences, labeled with three affinity categories: high, medium, and low. Our goal was to evaluate each model’s ability to accurately discriminate between high-affinity binders, medium-affinity binders, and low-affinity non-binders. This was designed to simulate a realistic antibody design scenario when we are faced with a set of sequences with unknown binding labels. We test whether a model, in this case inverse folding, has capacity to enrich top binders. It must be noted that in no case here do we talk about zero-shot predictions, as HER2 and Trastuzumab were likely seen by all models involved.

For comparison, we also added well known sequence only models (AbLang2, ESM2_650M), structure-aware sequence model SaProt and basic PWM as baseline.

**Figure 1:**
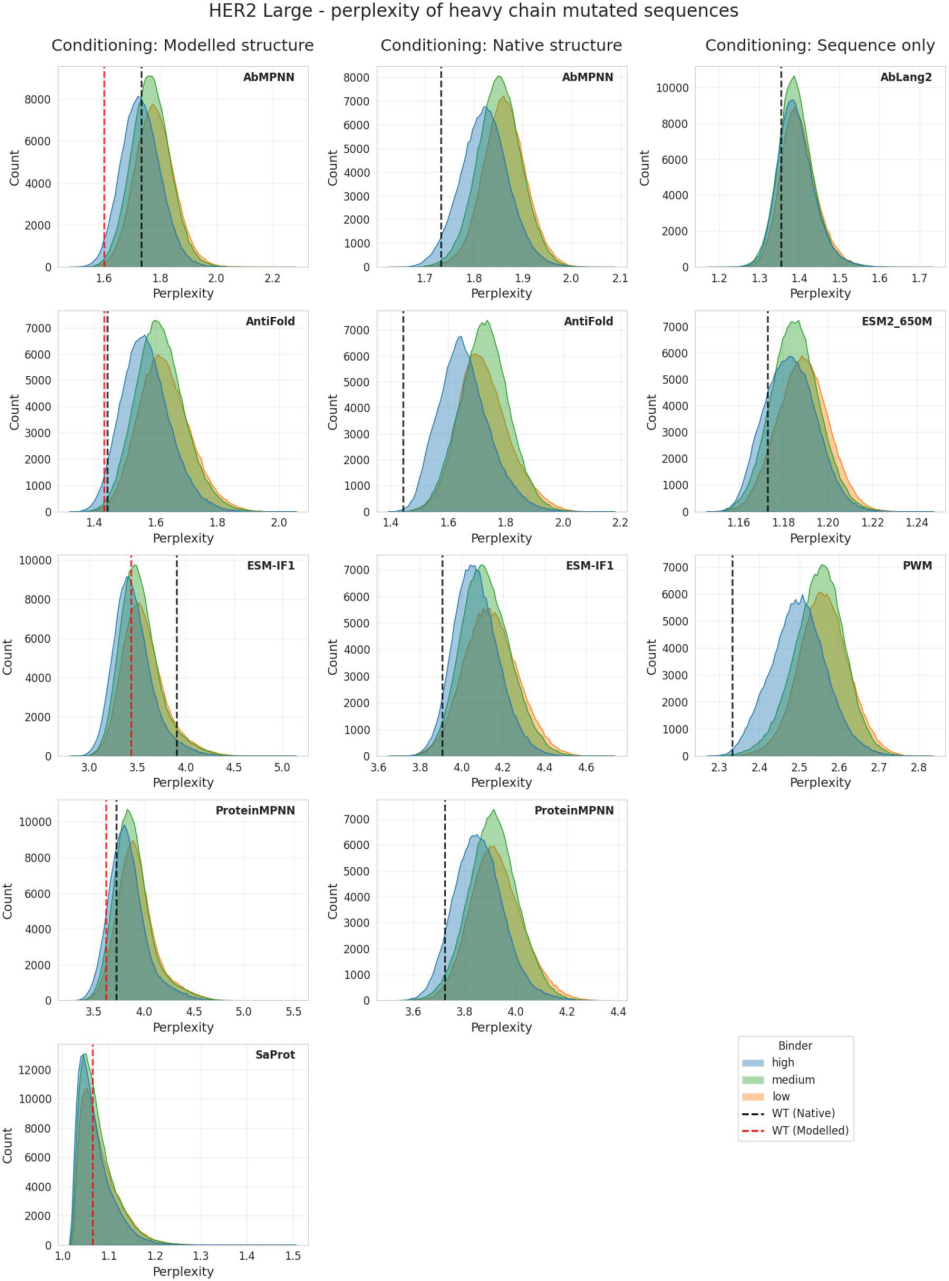
**Sorting sequences by perplexity provides a degree of separation of binders and nonbinders**. For each sequence in the HER-2 datasets, we calculated perplexity using one of four models ESM-IF, ProteinMPNN, AntiFold, or AbMPNN conditioned either on the native Trastuzumab structure or on the ABodyBuilder2 model. Black lines indicate native sequence (Trastuzumab) conditioned on native structure. We also added sequence-only models (AbLang2, ESM2_650M), structure-aware sequence model (SaProt) and baseline PWM to the comparison.

First, we performed a simple check to see whether sorting the model’s output has any predictive power in separating binders from nonbinders on this dataset. We plotted histograms of the perplexity scores across these affinity groups under two structural conditioning scenarios: native structures and structures modeled with ABodyBuilder2 (Fig. 1). Additional analysis revealed that while all benchmarked models favored mutants with higher sequence identity to the wild-type (Supplementary Figure 45, available as supplementary data at *Bioinformatics* online), strictly preserving Trastuzumab’s core contact residues did not significantly influence the perplexity scores (Supplementary Figure 44, available as supplementary data at *Bioinformatics* online). It is clear that regardless of the model or whether we employ the native structure, there is a certain degree of separation between high/medium/low affinity binders. The main question is whether this slight degree of separation translates to an enrichment that is useful in an antibody engineering scenario.

**Figure 2:**
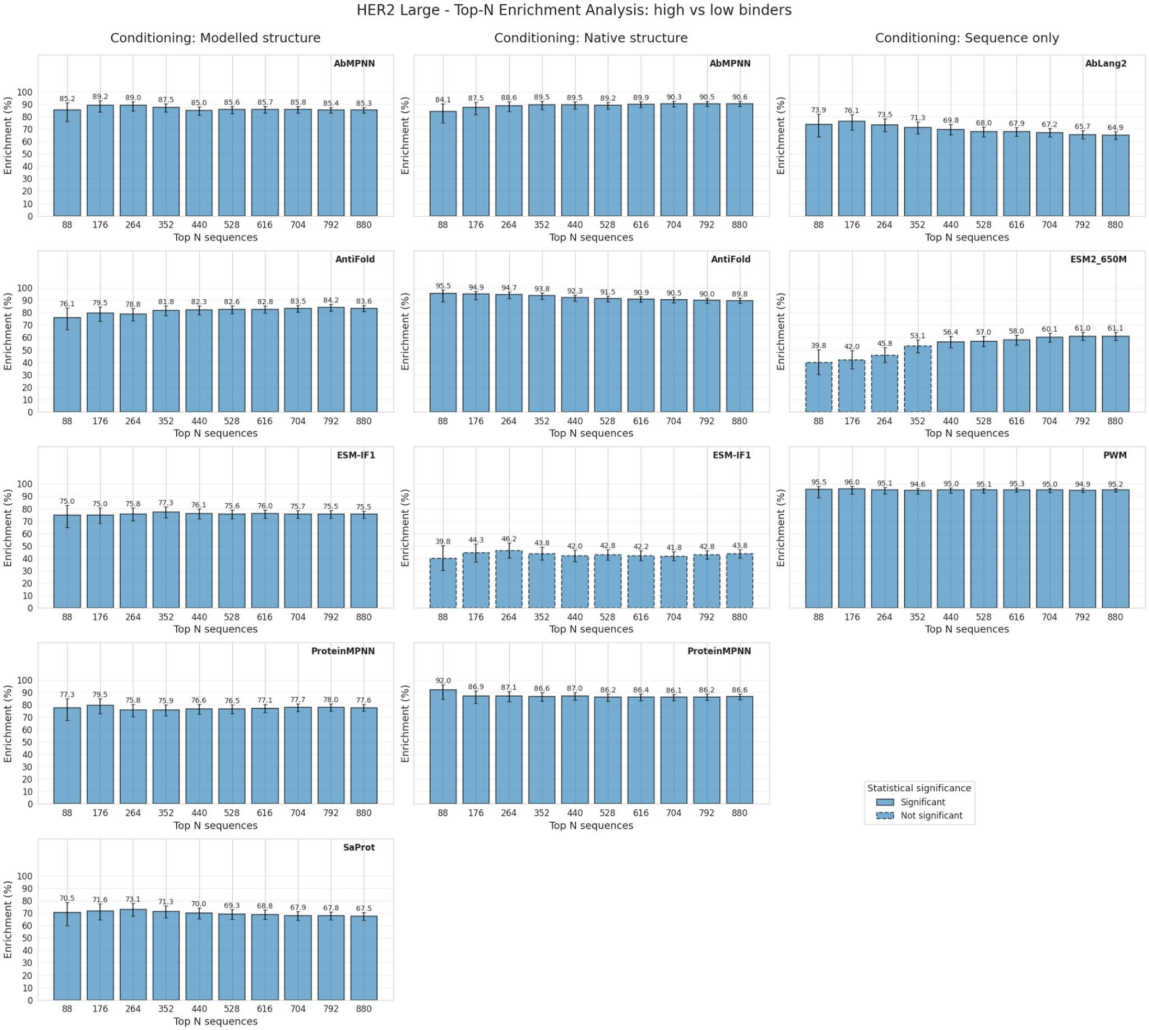
**Sorting the top-N sequences based on perplexity—low vs high binders**. The bins are multiples of 88, simulating a scenario where we get to select top N sequences from a larger set of unknown binders/nonbinders and can test them in batches of 88. We removed medium binders for this analysis. ESM-IF1 conditioned on native structure showed as outlier, due to the high number of low binders labelled with minimum perplexity. We investigated this further in Supplement (figure 30).

**Figure 3:**
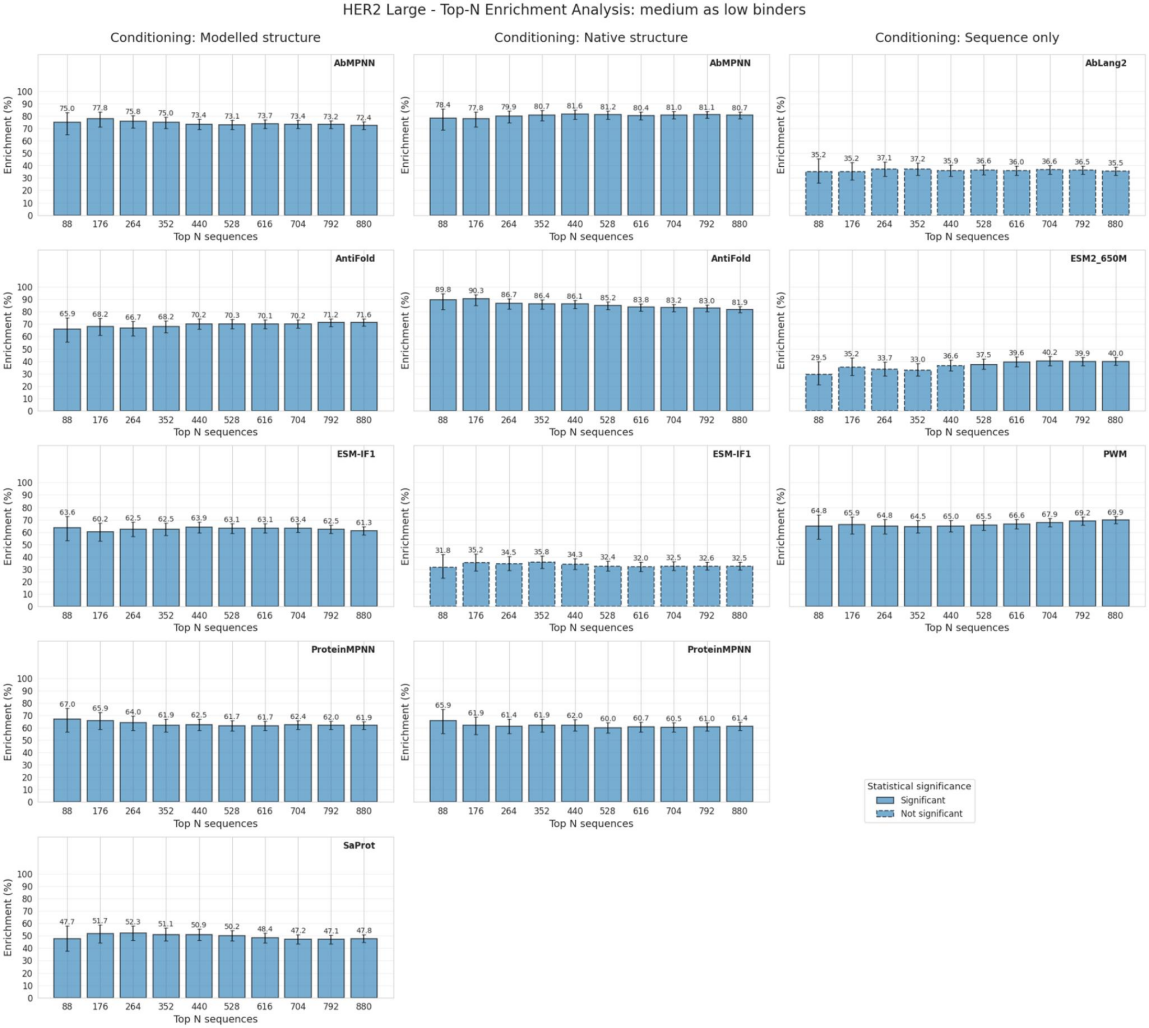
Sorting the top-N sequences based on perplexity—medium are treated as nonbinders.

To quantify whether inverse folding models can effectively enrich high-affinity binders in sequences sorted by low perplexity, we analyzed the HER2-aff-large dataset using microplate-scale sampling (88-well increments, testing *N *= 88, 176, 264, …, 880 sequences). We addressed two complementary questions: (1) enrichment of high binders when distinguishing from low binders (Fig. 2), and (2) enrichment of high binders when medium-affinity sequences were classified as non-binders (Fig. 3). All six inverse folding models were evaluated under three conditioning strategies: sequence-only, modeled antibody structure (ABodyBuilder2), and native crystal structure.

To rigorously quantify the statistical significance and uncertainty of enrichment metrics across sampling thresholds, we applied a hypergeometric test combined with confidence interval estimation and multiple testing correction. For each threshold, we calculated exact p-values using the hypergeometric distribution, which precisely models our experimental design of sampling without replacement from a finite population. We computed 95% Wilson Score confidence intervals for each enrichment ratio, a method preferred in biostatistical analyses for its superior coverage properties relative to standard approximations. To account for multiple hypothesis testing across thresholds, we applied Benjamini-Hochberg false discovery rate (FDR) correction, which effectively controls the expected proportion of false discoveries while maintaining statistical power. Enrichment metrics are presented with point estimates, confidence intervals, and FDR-adjusted p-values with statistical significance indicators.

The antibody-specific models demonstrated complementary strengths depending on structural conditioning: AbMPNN exhibited superior performance with modeled structures, achieving 85.2% enrichment (high vs. low binders) at the first sampling threshold (*N *= 88) and maintaining 85.3% even at *N *= 880, demonstrating exceptional consistency across the entire sampling range. When medium binders were classified as non-binders, AbMPNN achieved 75.0% enrichment with modeled structures. In marked contrast, AntiFold dominated when conditioned on native structures, reaching exceptional enrichment of 95.5% for high vs. low classifications at *N *= 88 and sustaining 89.8% at *N *= 880—a 11.4 percentage point advantage over AbMPNN’s 84.1% on native structures. When medium binders were reclassified as non-binders, AntiFold achieved 89.8% enrichment with native structures compared to AbMPNN’s 78.4%, further highlighting these complementary strengths. Notably, the sequence-only PWM model achieved exceptional enrichment of 95.5% for high vs. low binders, matching AntiFold’s native structure performance and substantially exceeding all other models on this task. However, PWM’s enrichment declined substantially to 64.8% when medium binders were reclassified as non-binders, indicating lower discrimination capability in more challenging classification scenarios. ProteinMPNN and ESM-IF1 exhibited substantial performance degradation under native structure conditioning, with ESM-IF1 showing minimal enrichment (31.8–46.2%) despite strong performance in the modeled structure setting (75.0%). This drastic drop for ESM-IF1 is primarily driven by a high number of low-affinity binders being assigned minimum perplexity scores, an anomaly we explore further in Supplementary Figure 46, available as supplementary data at *Bioinformatics* online.

Other sequence-only models demonstrated markedly limited enrichment capability, with AbLang2 achieving only 73.9–76.1% on high vs. low tasks and declining to 35.2% when medium binders were treated as negatives, while ESM2-650M remained below 65% across all conditioning scenarios. Persistent statistical significance across all sampled thresholds and conditioning approaches (FDR *P* < 0.05 for well-performing models) indicates genuine model enrichment of high-affinity binders rather than spurious effects arising from random variation.

These results demonstrate that domain-specific inverse folding models substantially outperform general protein folding models when tasked with antibody-antigen affinity prediction, and reveal how model choice and structural conditioning strategy present complementary trade-offs: AbMPNN for robust performance with predicted structures, AntiFold for superior enrichment when native structures are available, with sequence-only approaches showing promise for simple binary discrimination but limited utility for nuanced affinity prediction.

### Anti-PD1 - internal BI dataset

Following our assessment of inverse folding methods on the publicly available HER2 dataset, we investigated the applicability of these approaches in an industrial context using an internal BI dataset comprising PD1 binder and non-binder antibody pairs. This dataset includes 59 distinct heavy and light chain sequences of varying lengths. Due to the absence of native structural information, we relied entirely on structures modeled using ABodyBuilder2 for all analyses. Evaluation was conducted by conditioning heavy chain sequences on their corresponding modeled structures, with a balanced dataset (50% binders, 50% non-binders).

Initially, we examined the perplexity score distributions of binders and non-binders across all four models: ESM-IF, Antifold, ProteinMPNN, and AbMPNN (Fig. 4). Consistent with our HER2 observations, there was modest enrichment of binders at the lower perplexity scores, although the separation was less pronounced due to the smaller dataset size.

**Figure 4:**
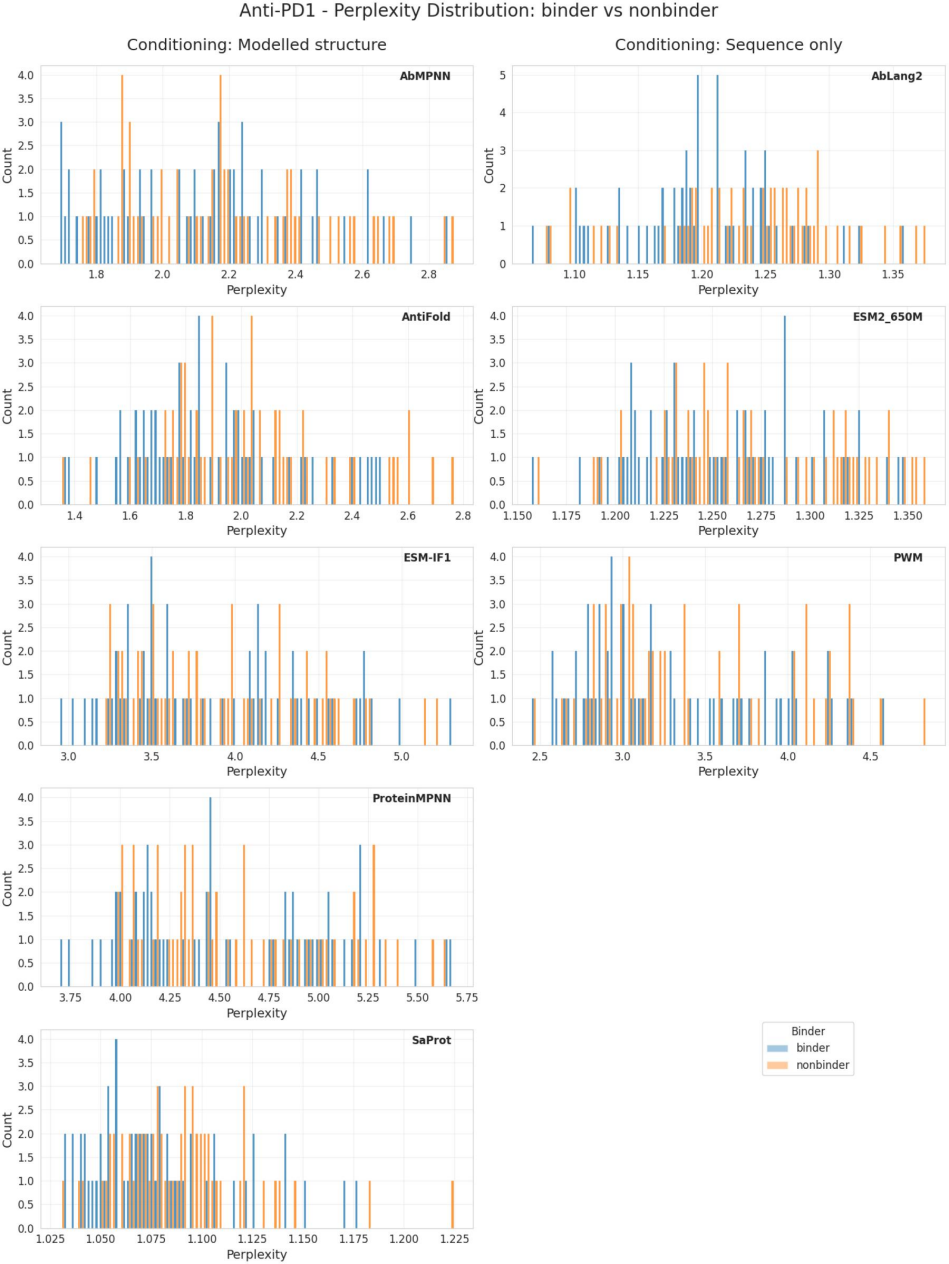
**Separation of binders vs non-binders for the BI-PD1 dataset. Top**: binders and non-binders histograms by their perplexities. Bottom: binders in top N sequences scored by perplexity, taken in batches of 12.

**Figure 4:**
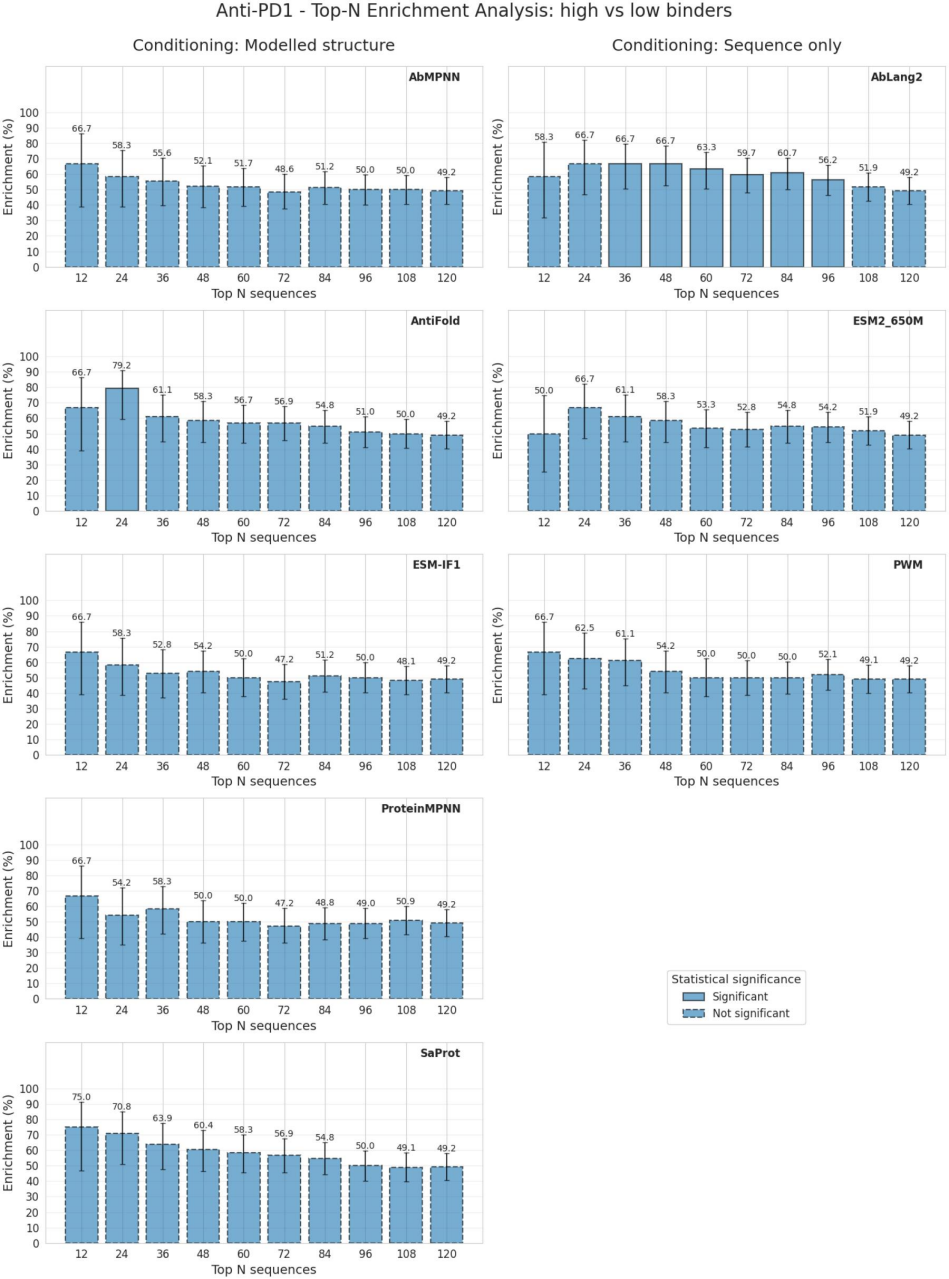
(Continued).

Among the tested models, Antifold provided the clearest differentiation between binder and non-binder sequences based on perplexity distributions, achieving a stable binder enrichment of 75% among the top 12 sequences, steadily declining toward the expected baseline as more sequences were considered. This indicates that Antifold reliably captures sequence features associated with higher affinity, even with limited data.

AbMPNN initially matched Antifold’s performance with 75% enrichment among the top 12 sequences. However, enrichment declined rapidly in larger sequence groups, suggesting lower stability and less robust separation between binder types. The stochastic nature inherent to ProteinMPNN-based models likely contributed to this performance variability, highlighting potential limitations when applied to smaller datasets.

ESM-IF, despite being a general-purpose model, surprisingly showed competitive initial performance, reaching 71% enrichment in the top 24 sequences. Nonetheless, this advantage quickly diminished beyond this threshold, dropping to about 54% at larger selections and demonstrating relatively limited ability to maintain robust binder enrichment across a wider dataset.

ProteinMPNN exhibited the weakest performance overall, with no clear separation between binders and non-binders. The maximum observed enrichment was only 67%, and enrichment was inconsistent, with binder peaks appearing at both moderate and higher perplexity scores. This further underscores the advantage of antibody-specific models, particularly in datasets with limited sequence diversity and size.

Collectively, our analysis demonstrates that antibody-specific inverse folding models, particularly Antifold, perform more reliably and effectively in discriminating binders from non-binders in industrial-scale datasets. General protein models and stochastic antibody-specific models (e.g., AbMPNN) exhibit variable performance, underscoring the need for further optimization or larger datasets for consistent predictive accuracy.

### Performance on AbDesign DB

The previous HER2 and PD1 datasets served to demonstrate that there is some predictive power in applying inverse folding models to surface antibodies with a higher likelihood to bind, out of a set of known binders/non-binders. In these datasets, however, it was impossible to gauge to what extent the antigen affects the performance of the models. In the case of HER2, the mutants were typically multiple mutations away from the parent Trastuzumab, so modeling the complex between it and HER2 would be unreliable in the first place. In the case of PD1, we had no way of modeling the interaction between the sequences and PD1 as antibody-antigen docking is still an open problem (Hitawala and Gray 2024).

To address this, we employed the NaturalAntibody AbDesign DB. The resource consists of 14 antibody-antigen complexes, seven targets, and two antibodies per target. There are ca. 700 point mutants of CDR-H3, measured by ELISA. Therefore, we know the precise structural configuration, the mutations, and the resulting effect on binding. The effect of mutation is measured as a ratio of ELISA binding of the mutant to the wild type. Therefore, values close to 1.0 indicate maintenance of binding at least as good as the parent, whereas values closer to 0.0 indicate loss in binding.

We applied the inverse folding models to it to check whether the single-point mutation effect can be predicted. We performed conditioning on the model of the antibody as well as native structure to see the effects.

For each of 14 complexes, we plotted the perplexity score versus the ELISA ratio (Supplementary Figure 4–11, available as supplementary data at *Bioinformatics* online). An ideal correlation would be an increasing linear slope from left to right, however, in many cases, the tendency was negative. We collated the overall correlations in Table 1. In multiple instances better scores favor native structures, however this is not a norm. Furthermore, the antibody-specific version AntiFold, performs better than ESM-IF, retaining some predictive power in the models, though a very weak one. There appears to be a minor improvement in correlation, while using antigen, but it is very weak, if any. We recognize that our proxy binding metric of ELISA ratio might be indicative in a more binary fashion whether there is maintenance of binding. Otherwise, the correlation, as would be expected with affinity, is quite tenuous.

**Table 1:** Comparison of correlations between perplexities and the ELISA ratio on NaturalAntibody AbDesign dataset.

Model	Conditioning type	Single chain pearson		Multi chain pearson	
		Correlation	*P*-value	Correlation	*P*-value
AbMPNN	modelled	0.30	4.10E-15	0.26	6.34E-12
	native	0.33	3.10E-18	0.32	1.38E-17
AntiFold	modelled	0.40	3.32E-27	0.38	5.24E-24
	native	0.34	1.88E-19	0.36	3.76E-22
ESM-IF1	modelled	0.18	3.72E-06	0.22	1.03E-08
	native	0.28	5.10E-13	0.29	6.63E-14
ProteinMPNN	modelled	0.33	5.70E-18	0.3	8.38E-15
	native	0.31	1.47E-16	0.27	8.50E-13
SaProt	modelled	0.24	7.37E-10	0.24	1.03E-09
PWM	sequence only	0.30	6.39E-15	–	–
ESM2_650M	sequence only	0.21	1.05E-07	–	–
AbLang2	sequence only	0.21	6.93E-08	–	–

To account for unreliability of the correlation scheme, we reformulated the task in a binary fashion, defining binding as everything above 1.0 and non-binding as below 1.0 (Supplementary Figures 12–19, available as supplementary data at *Bioinformatics* online). This was supposed to simulate a scenario where we enumerated the mutations in question, calculated perplexities and only kept as binding those with scores above the wild type 1.0 If the scores have any predictive power, the box plot for each of the PDBs should be tending towards zero towards the left, and towards one to the right. We counted how many such PDBs had tendency in the direction of making correct predictions and called it a success, with the results in Table 2. Again, the performance of native structures greatly outperforms the models, underpinning the need for better structural predictions. Including the antigen in the equation did not appear to improve matters.

**Table 2:** Success rate of picking the mutants that maintain binding.

Model	Conditioning type	Multi chain success (*n *= 14)	Single chain success (*n *= 14)
Antifold	modeled	5	5
	native	10	10
ESM-IF	modeled	3	4
	native	11	9

The limited influence of antigen context on current inverse folding predictions, as identified in recent benchmarking studies, can be attributed to the overwhelming dominance of structural constraints over functional binding signals. We argue that models such as AntiFold and ESM-IF1 are optimized primarily to maximize the likelihood of a sequence adopting a specific backbone geometry, effectively prioritizing “foldability” and internal loop stability over the subtler, sparser interactions required for antigen specificity. This tendency is exacerbated by training datasets populated largely by stable, single-chain globular proteins, leading general models like ProteinMPNN to misinterpret the unique, polar nature of transient antibody-antigen interfaces as analogous to the hydrophobic cores of protein complexes. Furthermore, the absence of contrastive objectives—where models explicitly learn to distinguish non-binders from binders sharing the same fold—allows the system to minimize training loss by learning generic “valid antibody” features rather than antigen-dependent rules. Consequently, structural validity acts as a statistical shortcut, resulting in models that excel at constructing geometrically plausible CDR loops while remaining largely insensitive to the specific chemical environment of the target antigen.

In conclusion, the inverse folding scheme can be used, when starting from a native binder structure. If a model is used the results will not be as reliable. There appears to be a very weak effect of antigen on the predictions which leaves a lot of room for improvement of the methods.

### Testing the efficacy of sampling and oracle schemes

The previous analyses focused on pre-existing datasets to assess whether inverse folding scores could enrich for favorable sequences. While this demonstrates whether a model has a tendency to rank binders above non-binders, it does not address a more practical question: whether such sequences would actually be *generated* by the models during sampling.

To investigate this, we evaluated whether sampling from inverse folding models produces sequences that are predicted to bind. We trained oracle classifiers (CNN architecture following (Mason *et al.* 2019)) on the HER2 Large dataset under three configurations: (i) using only CDRH3 sequences with medium binders treated as low (Supplementary Figures 20–21, available as supplementary data at *Bioinformatics* online), (ii) using only CDRH3 sequences with medium binders removed (Supplementary Figure 22, available as supplementary data at *Bioinformatics* online), and (iii) using full heavy-chain sequences with medium binders treated as low (Supplementary Figure 23, available as supplementary data at *Bioinformatics* online). These classifiers were then used to label sampled sequences as binders or non-binders. As a sanity check, we additionally evaluated 10,000 randomly generated CDRH3 sequences. In all cases, sampling and classification were restricted to CDRH3.

We observed that sampling at low temperatures—i.e. close to the original binder sequence—can be problematic. Sampling is performed residue-wise from multivariate distributions produced by the model, and at low temperatures these distributions become strongly biased, causing the sampling process to repeatedly converge to near-identical sequences. As a result, the number of unique sequences generated is extremely low (≈0.1% or less at temperature 1 for fine-tuned models). While such biased sampling makes exploration of the binding-relevant region of sequence space far more tractable than uniform sampling over the full combinatorial space (e.g. 201020^{10}2010 possibilities for CDR-H3), most generated sequences still fall outside the experimentally observed binder set, and many may not form productive CDR-H3 loops. Importantly, focusing only on single or double mutants would underestimate how many sequences outside the observed set are non-binders, as randomized mutagenesis at low mutation orders can still retain substantial binding (Janusz *et al.* 2025). In practice, MPNN-based models generate approximately 10% more binders and reach higher sequence diversity more rapidly—over 100 unique sequences versus 36 for AntiFold within comparable sampling time.

We found that some of these issues can be partially mitigated by alternative sampling strategies, such as enumeration followed by sorting by perplexity. In this setting, the effect of temperature can be incorporated implicitly by filtering sequences based on sequence identity to the parental binder, rather than relying solely on temperature scaling.

A second issue concerns the reliability of the oracle classifiers themselves. Although training on large binder/non-binder datasets has been shown to yield experimentally actionable models (Mason *et al.* 2021; Lim *et al.* 2022), we observed substantial variation in prediction accuracy depending on whether medium-affinity binders were included as positives or treated as non-binders (Supplementary Figures 20–27, available as supplementary data at *Bioinformatics* online), highlighting the sensitivity of the task definition.

Most concerning were the sanity checks on random sequences (Supplementary Figures 36, 38, and 39, available as supplementary data at *Bioinformatics* online). A substantial fraction of randomly generated CDRH3s were classified as binders—5.6%, 38.0%, and 10.6% depending on the oracle configuration. Even under conservative labeling, it is striking that as many as 5.6% of random CDR-H3 sequences would be predicted to bind, although this was not experimentally verified. This suggests that the oracles may capture generic antibody-like features rather than true binding specificity. For instance, we checked to what degree ABLang2 distinguishes such sequences as well and it separated random and HER-2 dataset sequences in a zero-shot fashion (Supplementary Figure 47, available as supplementary data at *Bioinformatics* online). This stands to show that it is rather the naturalness that is captured and this is recapitulated by great results of PWMs on the same dataset.

### Sampled datasets statistics

**Figure 5:**
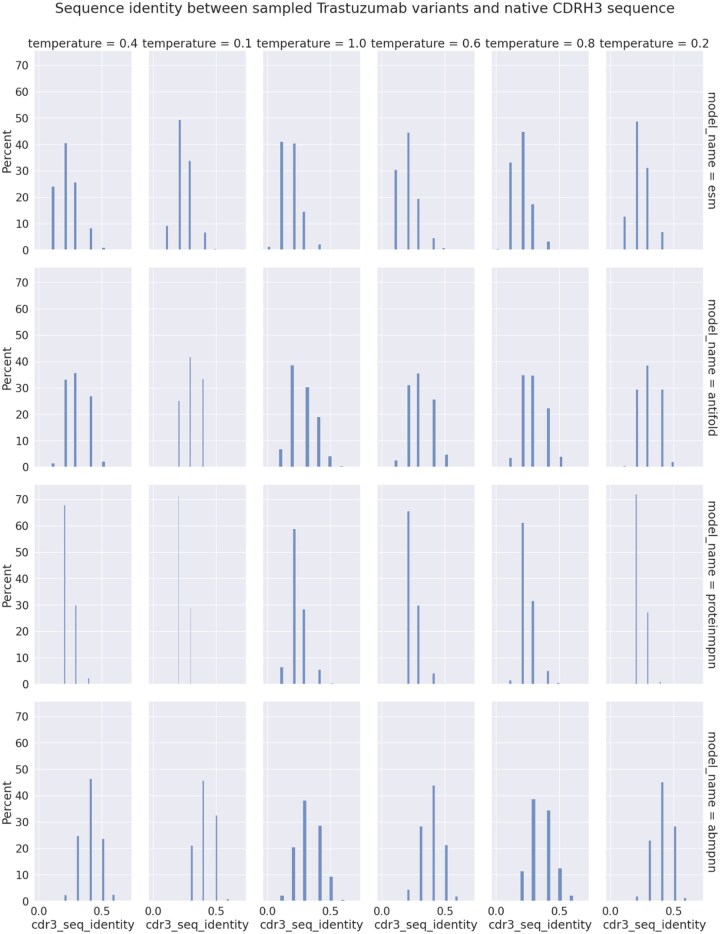
**Sequence identity distributions between sampled and native CDRH3 sequences**. Each model tends to output similarly distant sequences across different temperature settings. Sequence identity drops at most ∼10% between 0.1 and 1.0 temperature for ESM-IF and ProteinMPNN. For antibody-specific models the drop is even less (∼5%).

**Figure 6:**
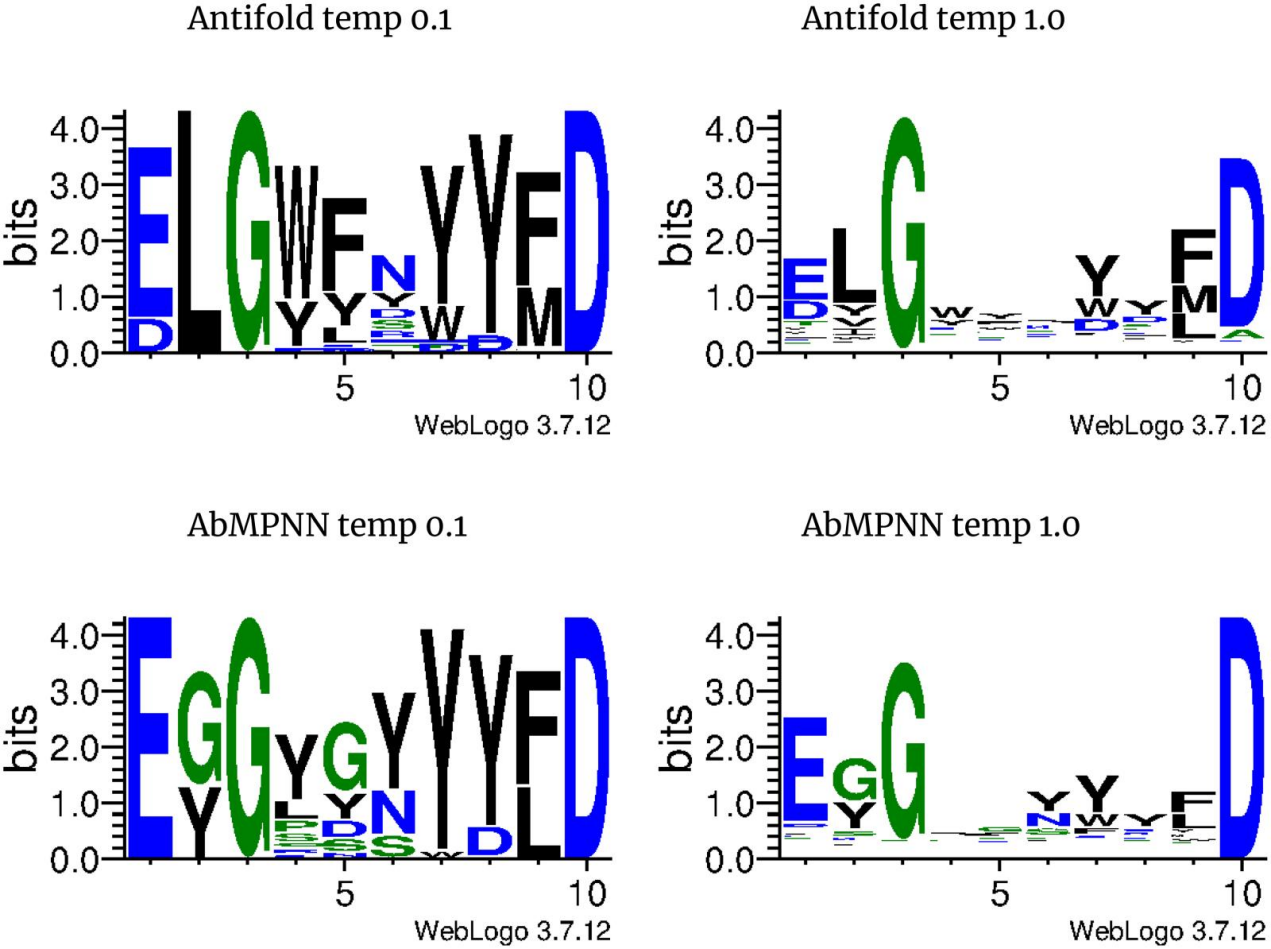
**WebLogo residue distributions for Antifold and AbMPNN sampled CDRH3 sequences**. We can see that 3rd (G) and 10th (D) positions are highly conserved for both AntiFold and AbMPNN.

The goal of the sampling procedure was to have 1000 unique sequences for each model-temperature combination. However, because of extremely long sampling time at lower temperatures (models generating a large number of non-unique sequences), we stopped the sampling with much smaller numbers of sequences (Table 3). We calculated sequence identity between each sampled CDRH3 sequence and the native one taken from Trastuzumab. We noticed that identity drops with rising temperature, which is the expected behavior, but to our surprise, not to a big extent (Fig. 5). Residue distributions confirmed that even with drastic temperature differences (0.1 vs 1.0), residues generated on some positions are highly conserved (Fig. 6).

**Table 3:** Sampled datasets counts.

	Sampling temperature					
Model name	0.1	0.2	0.4	0.6	0.8	1
AbMPNN	114	553	774	841	979	1000
Antifold	36	200	500	1000	964	999
ESM-IF	336	500	1000	999	1000	1000
ProteinMPNN	194	470	944	996	1000	1000

Counts of unique sequence sets sampled using 4 models on different temperature settings. The higher the temperature, the more diverse sequences a model produces, hence easier (faster) to generate unique examples. For the lowest (0.1) temperature, Antifold was generating identical sequences most of the time, thus such low cardinality (36) of the corresponding dataset.

## Discussion

Generative methods hold great promise in accelerating the costly and arduous experimental design process of biologics discovery. To this end, we benchmarked structure-aware generative methods of the inverse-folding disposition. This was dictated by desire to see whether these methods are useful in a realistic discovery setting and to what extent the antigen influences the predictions.

Our analysis confirms observations of other studies that, given a set of antibodies, these methods are useful in enriching for the higher-affinity antibodies (Hie *et al.* 2024). They do so in a manner agnostic to the antigen, and they can operate on a model of the antibody sequence. Therefore, they are suitable for NGS datasets from display or immunizations, as typically used in the course of discovery campaigns (Erasmus *et al.* 2023).

We tested the methods on our own internal dataset that was supposed to reveal to what extent the antigen information is taken into account. This test revealed that the antigen does not inform the predictions a great deal, if at all, as confirmed by other studies on this topic (Uçar *et al.* 2024).

Altogether, our findings suggest that the inverse folding models are moderately useful in an industrial setting for filtering NGS datasets. There is however large room for improvement to bias the datasets in the direction of taking the binding interface into account. Currently it appears that similarly to language models, the models are primed to focus on the more conserved patterns of antibody sequences, which can be predicted without any resort to the antigen (Uçar and Sormanni 2025).

While inverse folding models have demonstrated utility in filtering NGS datasets, their current reliance on structural stability often overshadows the critical requirement of binding affinity optimization. Significant opportunities exist to bias these models toward functional interface design. AntiFold, despite its high sequence recovery, exhibits minimal dependence on antigen context; integrating explicit cross-attention mechanisms between antibody and antigen encoders, alongside contrastive learning objectives based on experimental binding data, would shift its focus from loop stability to interface complementarity. For graph-based models like ProteinMPNN and AbMPNN, the incorporation of ligand-aware nodes (as seen in LigandMPNN) is essential for targeting non-protein epitopes, while the adoption of interface-specific edge weighting and physics-informed loss functions could prioritize polar interactions critical for high-affinity binding. Finally, AbMPNN can further improve its real-world applicability by optimizing noise-augmentation strategies specifically for homology-modeled backbones—going beyond standard Gaussian perturbations to address the specific error profiles of antibody structure predictors. Collectively, these advancements would transition inverse folding from a stability-centric filter to a potent tool for functional affinity maturation.

## Supplementary Material

vbag081_Supplementary_Data

## Data Availability

AbDesign DB: https://naturalantibody.com/ab-design/ Trastuzumab HER2 Large: https://zenodo.org/records/10831512 Anti-PD1 dataset: This dataset was provided internally by Boehringer-Ingelheim and is proprietary.

## References

[vbag081-B1] Abanades B , Wong WK, Boyles F et al ImmuneBuilder: deep-learning models for predicting the structures of immune proteins. Commun Biol 2023;6:575. 37248282 10.1038/s42003-023-04927-7 PMC10227038

[vbag081-B2] Bielska W , Jaszczyszyn I, Dudzic P et al Applying computational protein design to therapeutic antibody discovery - current state and perspectives. Front Immunol 2025;16:1571371. 10.3389/fimmu.2025.1571371. 40475769 PMC12137305

[vbag081-B3] Chinery L , Hummer AM, Mehta BB et al Baselining the buzz trastuzumab-HER2 affinity, and beyond. *bioRxiv*. March 29, 2024. 10.1101/2024.03.26.586756.

[vbag081-B4] Dauparas J , Anishchenko I, Bennett N et al Robust deep learning-based protein sequence design using ProteinMPNN. Science (New York, N.Y.) 2022;378:49–56. 36108050 10.1126/science.add2187 PMC9997061

[vbag081-B5] Dreyer FA, Cutting D, Schneider C et al Inverse folding for antibody sequence design using deep learning. *arXiv preprint arXiv:2310.19513*. 2023. https://arxiv.org/abs/2310.19513

[vbag081-B6] Erasmus MF , Ferrara F, D’Angelo S et al Insights into next generation sequencing guided antibody selection strategies. Sci Rep 2023;13:18370. 37884618 10.1038/s41598-023-45538-w PMC10603065

[vbag081-B7] Hie BL , Shanker VR, Xu D et al Efficient evolution of human antibodies from general protein language models. Nat Biotechnol 2024;42:275–83. 37095349 10.1038/s41587-023-01763-2 PMC10869273

[vbag081-B8] Hitawala FN, Gray JJ. What does AlphaFold3 learn about antibody and nanobody docking, and what remains unsolved? *mAbs* 2025;**17**. 10.1080/19420862.2025.2545601 PMC12360200 40814020

[vbag081-B9] Høie MH , Hummer AM, Olsen TH et al AntiFold: improved structure-based antibody design using inverse folding. Bioinform Adv 2025;5:vbae202. 40170886 10.1093/bioadv/vbae202 PMC11961221

[vbag081-B10] Hsu C , Verkuil R, Liu J et al Learning inverse folding from millions of predicted structures. bioRxiv 2022;162:8946–70.

[vbag081-B11] Hummer AM, Schneider C, Chinery L et al Investigating the volume and diversity of data needed for generalizable antibody-antigen ΔΔG prediction. *Nat Comput Sci* 2025;**5**:635–47. 10.1038/s43588-025-00823-8 PMC12374841 40629090

[vbag081-B12] Janusz B, Chomicz D, Demharter S et al AbDesign: database of point mutants of antibodies with associated structures reveals poor generalization of binding predictions from machine learning models. *MAbs* 2025;**17**:2567319. 10.1080/19420862.2025.2567319 PMC12520099 41058476

[vbag081-B13] Lim YW , Adler AS, Johnson DS. Predicting antibody binders and generating synthetic antibodies using deep learning. MAbs 2022;14:2069075. 35482911 10.1080/19420862.2022.2069075 PMC9067455

[vbag081-B14] Li Y, Lang Y, Xu C et al Benchmarking inverse folding models for antibody CDR sequence design. *PLoS One* 2025;20:e0324566. 10.1371/journal.pone.0324566 PMC12136355 40465588

[vbag081-B15] Mason DM , Friedensohn S, Weber CR et al Deep learning enables therapeutic antibody optimization in mammalian cells by deciphering high-dimensional protein sequence space. *Synthetic Biology*. Biorxiv; 617860v3 BioRxiv, April 24, 2019. https://www.biorxiv.org/content/10.1101/617860v2.full.pdf.

[vbag081-B16] Mason DM , Friedensohn S, Weber CR et al Optimization of therapeutic antibodies by predicting antigen specificity from antibody sequence via deep learning. Nat Biomed Eng 2021;5:600–12. 33859386 10.1038/s41551-021-00699-9

[vbag081-B17] Shanker VR , Bruun TUJ, Hie BL et al Unsupervised evolution of protein and antibody complexes with a Structure-Informed language model. Science 2024;385:46–53. 38963838 10.1126/science.adk8946 PMC11616794

[vbag081-B18] Uçar T , Malherbe C, Gonzalez F. Exploring log-likelihood scores for ranking antibody sequence designs. *Bioinformatics*. Biorxiv; 2024.10.07.617023v4. BioRxiv, October 11, 2024. https://www.biorxiv.org/content/10.1101/2024.10.07.617023v1.

[vbag081-B19] Uçar T , Sormanni P. BLOSUM is all you learn–generative antibody models reflect evolutionary priors. *Bioinformatics*. Biorxiv; 2025.10.26.684652v2. BioRxiv, October 27, 2025. https://www.biorxiv.org/content/10.1101/2025.10.26.684652v1.full.

[vbag081-B20] Varadi M , Anyango S, Deshpande M et al AlphaFold protein structure database: massively expanding the structural coverage of Protein-Sequence space with High-Accuracy models. Nucleic Acids Res 2022;50:D439–D444. 34791371 10.1093/nar/gkab1061 PMC8728224

