# Supplementary material for: Benchmarking antigen-aware inverse folding methods for antibody design
Source: Bioinform Adv. 2026 Mar 20;6(1):vbag081. doi: 10.1093/bioadv/vbag081 (PMC13140557; doi:10.1093/bioadv/vbag081)
Supplement: vbag081_Supplementary_Data [file vbag081_supplementary_data.zip › Supplementary information inverse folding benchmarking.docx]

**Supplementary information.**


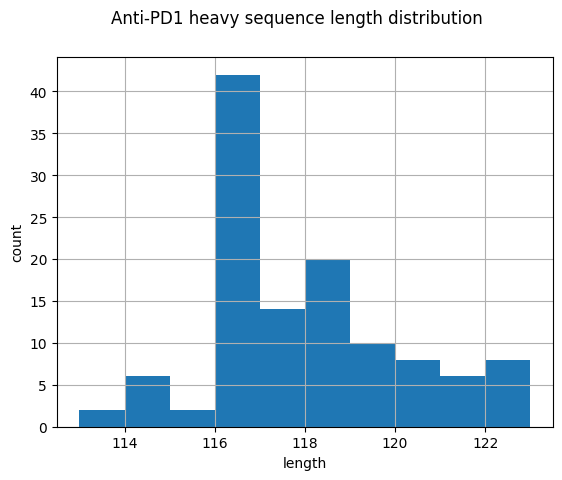

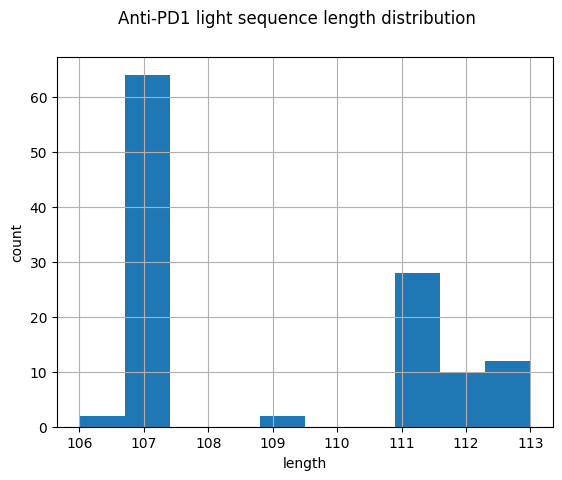

**Supplementary Figure 1: Anti-PD1 heavy (Left) and light (Right) variable region sequence length distribution.**

Binder Non-binder


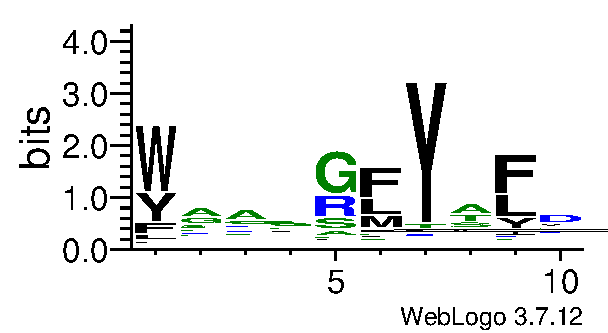

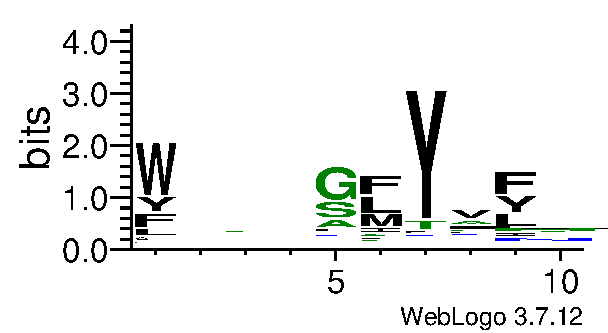


**Supplementary Figure 2: WebLogo residue distributions for HER2-large-aff CDRH3 sequences -** medium labeled as low binders, binder (Left) and non-binder (Right). We can see 7th position is highly conserved.

Binder Non - binder


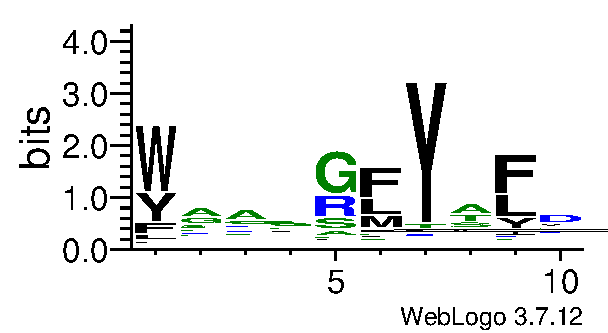

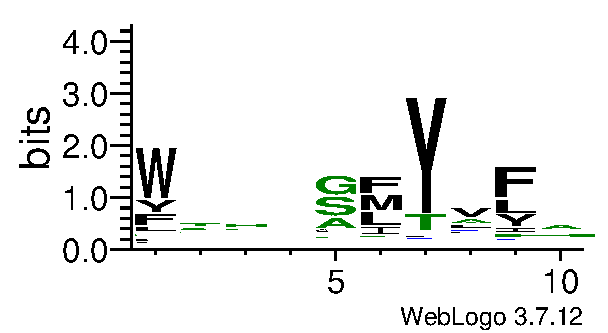


**Supplementary Figure 3: WebLogo residue distributions for HER2-large-aff CDRH3 sequences** - removed medium binders, binder (Left) and non-binder (Right). We can see 7th position is highly conserved.


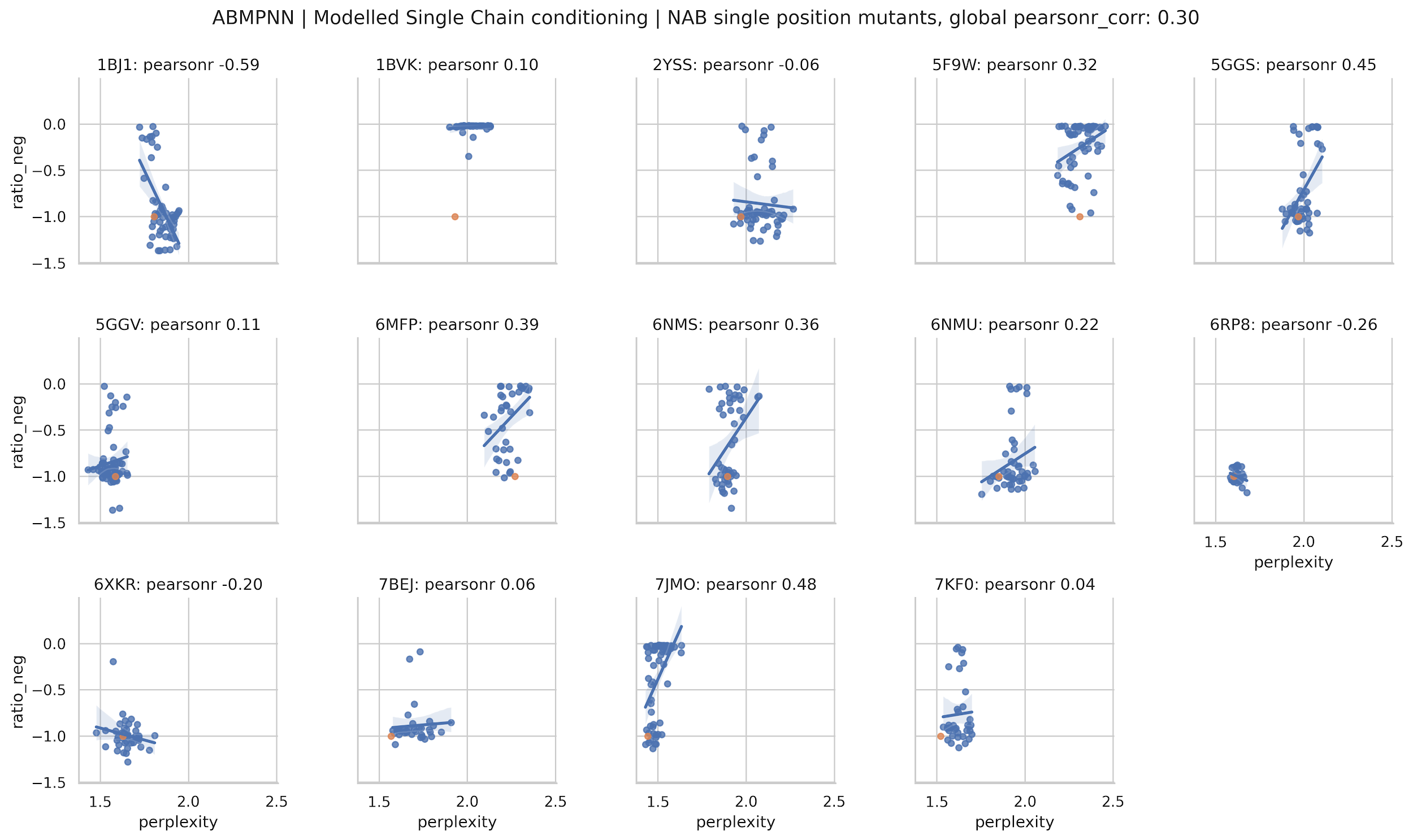


**Supplementary Figure 4. AbMPNN conditioned on modelled structure, no antigen.** Perplexity (x-axis) is plotted against the negative log ratio of the mutant/wt ELISA ratio (-1.0 is WT, closer to zero, worse binding).


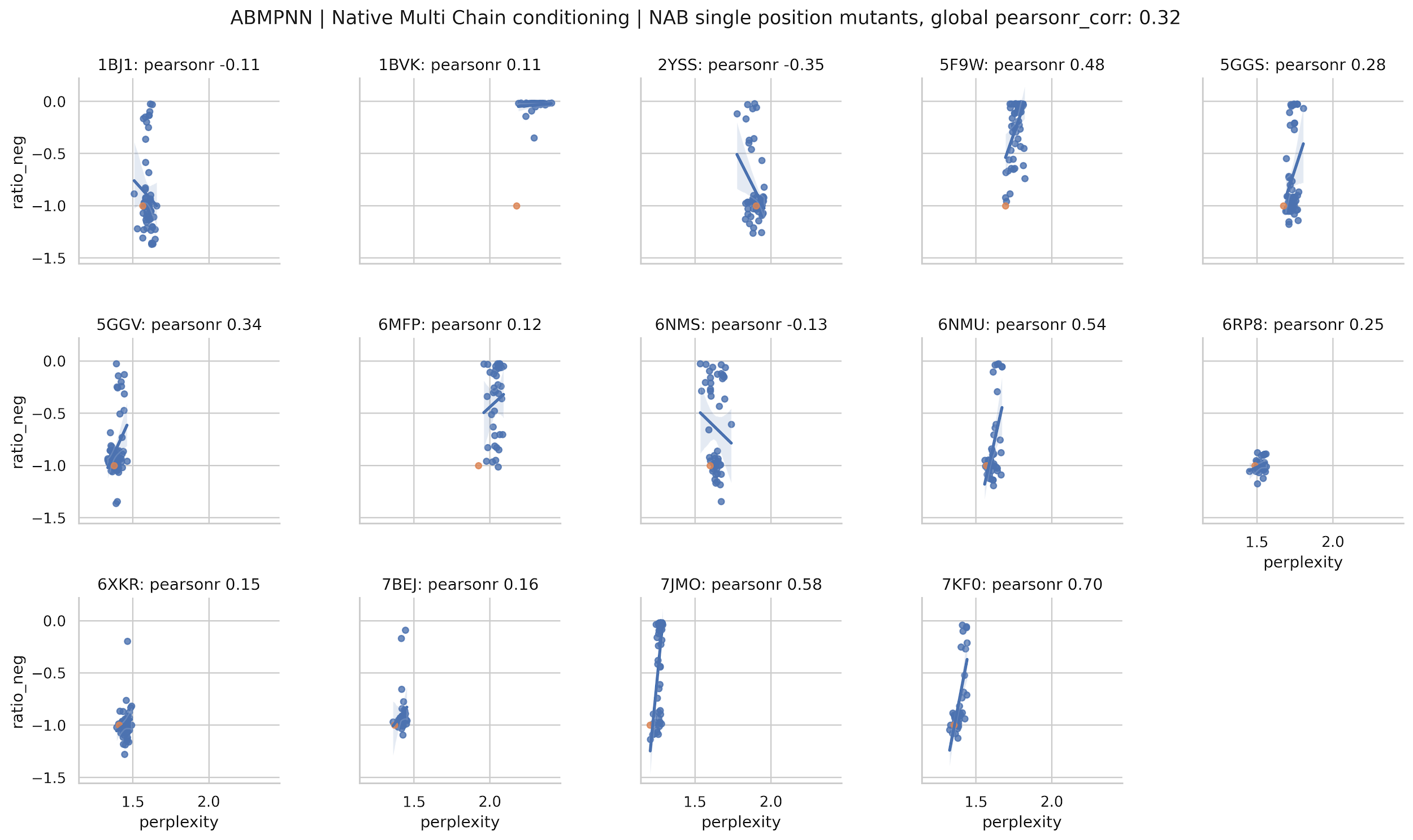
**Supplementary Figure 5. AbMPNN conditioned on native structure, with antigen.** Perplexity (x-axis) is plotted against the negative log ratio of the mutant/wt ELISA ratio (-1.0 is WT, closer to zero, worse binding).


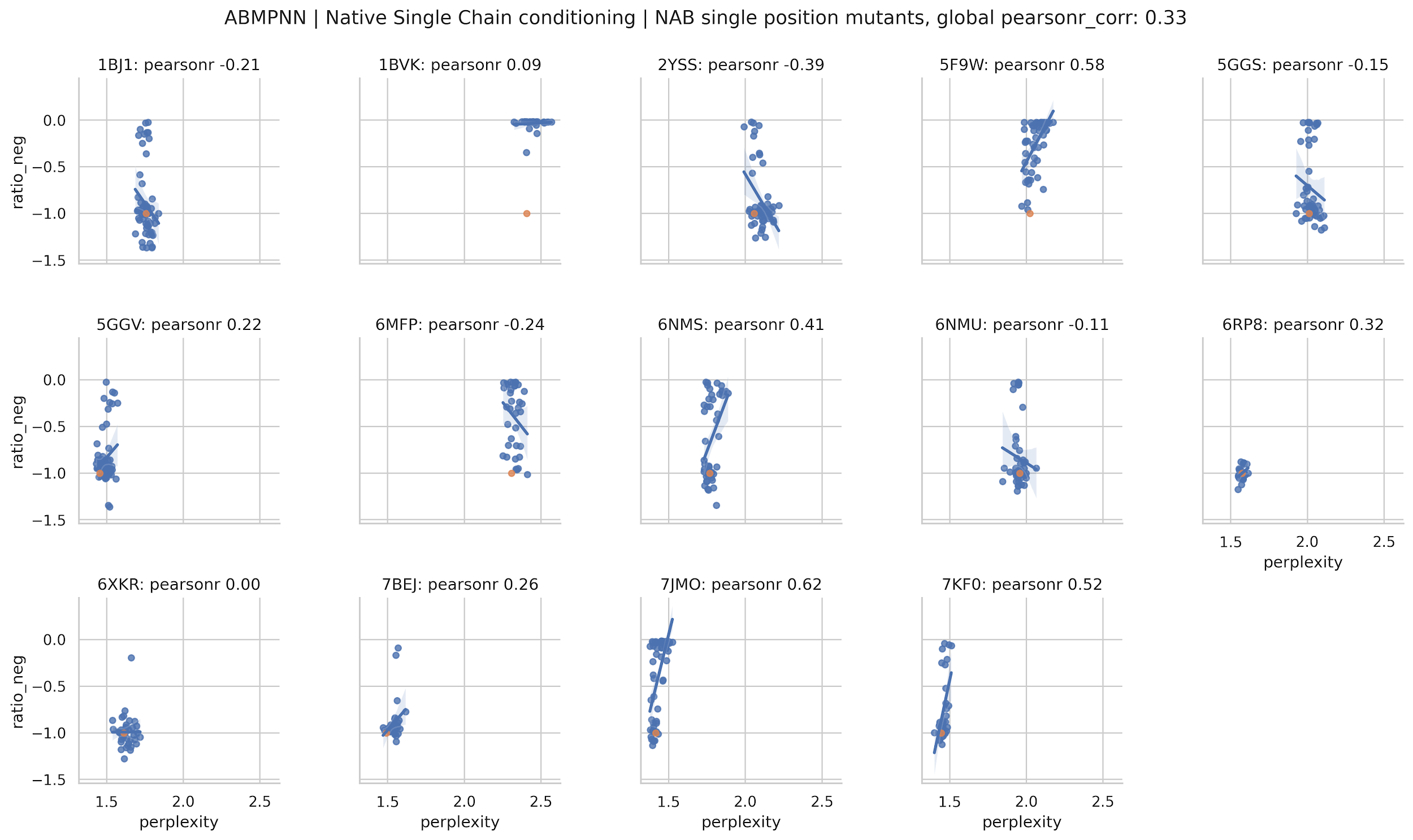
**Supplementary Figure 6. AbMPNN conditioned on native structure, no antigen.** Perplexity (x-axis) is plotted against the negative log ratio of the mutant/wt ELISA ratio (-1.0 is WT, closer to zero, worse binding).


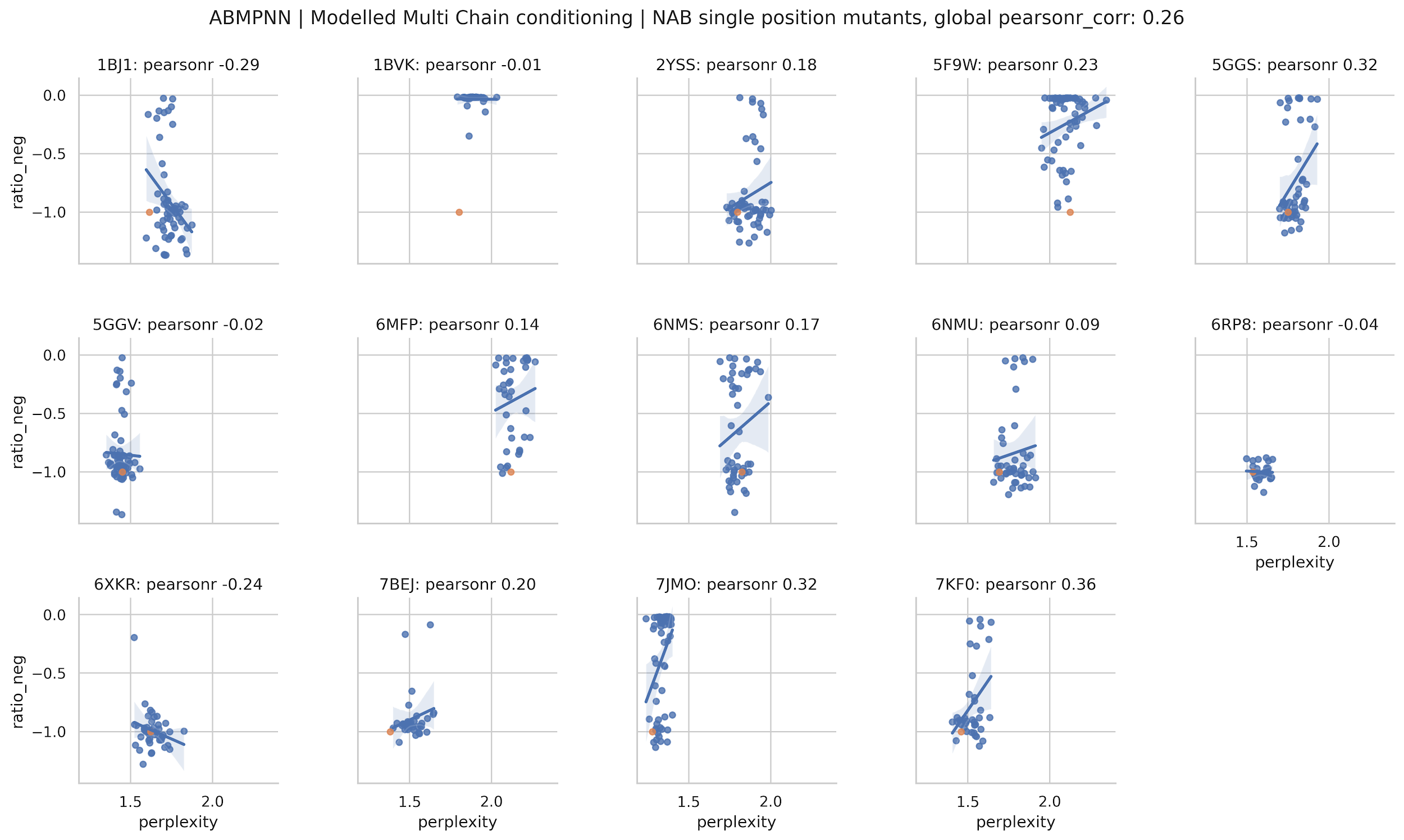


**Supplementary Figure 7. AbMPNN conditioned on modelled structure, with antigen.** Perplexity (x-axis) is plotted against the negative log ratio of the mutant/wt ELISA ratio (-1.0 is WT, closer to zero, worse binding).


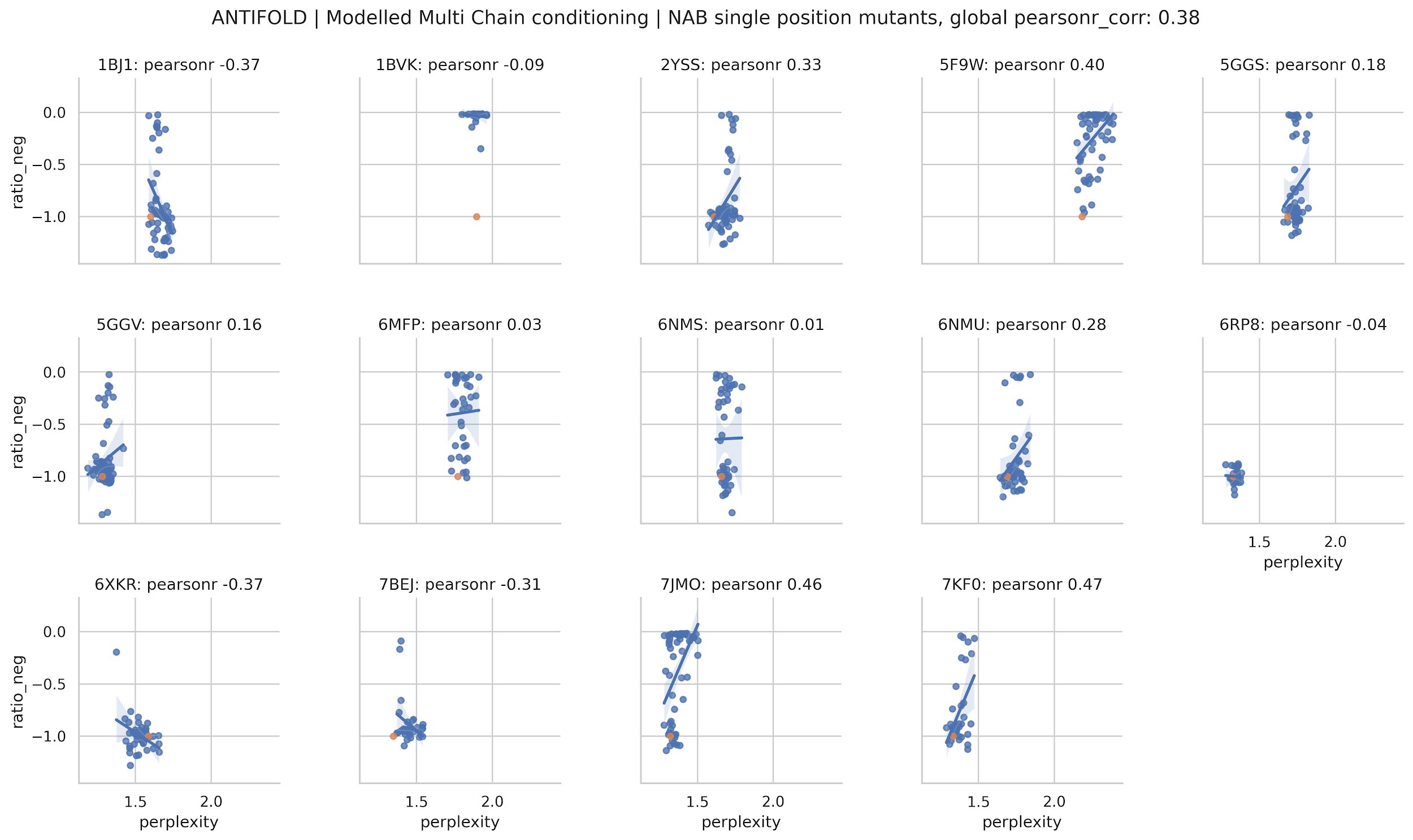
**Supplementary Figure 8. Antifold conditioned on modelled structure, with antigen.** Perplexity (x-axis) is plotted against the negative log ratio of the mutant/wt ELISA ratio (-1.0 is WT, closer to zero, worse binding)


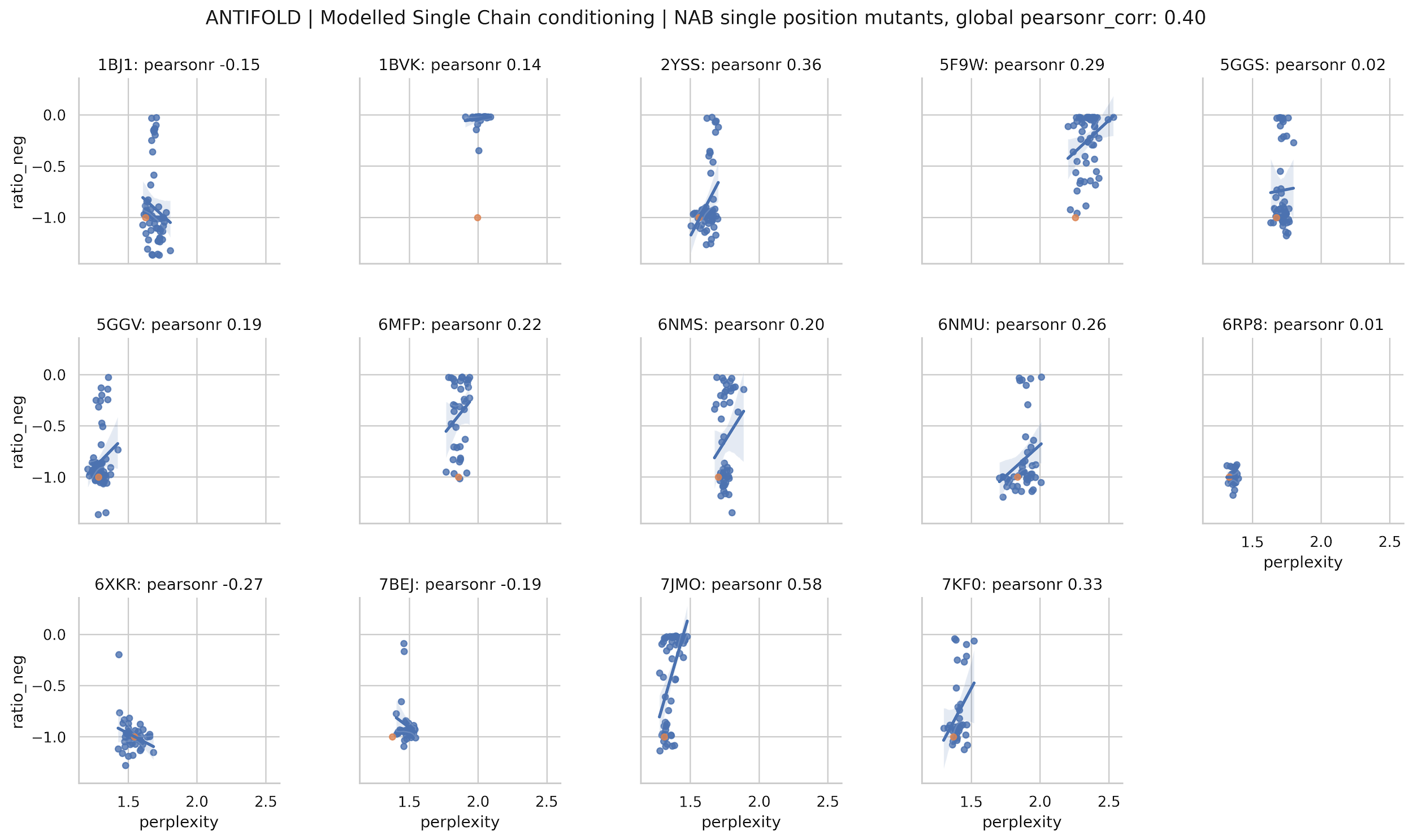
**Supplementary Figure 9. Antifold conditioned on modelled structure, no antigen.** Perplexity (x-axis) is plotted against the negative log ratio of the mutant/wt ELISA ratio (-1.0 is WT, closer to zero, worse binding)


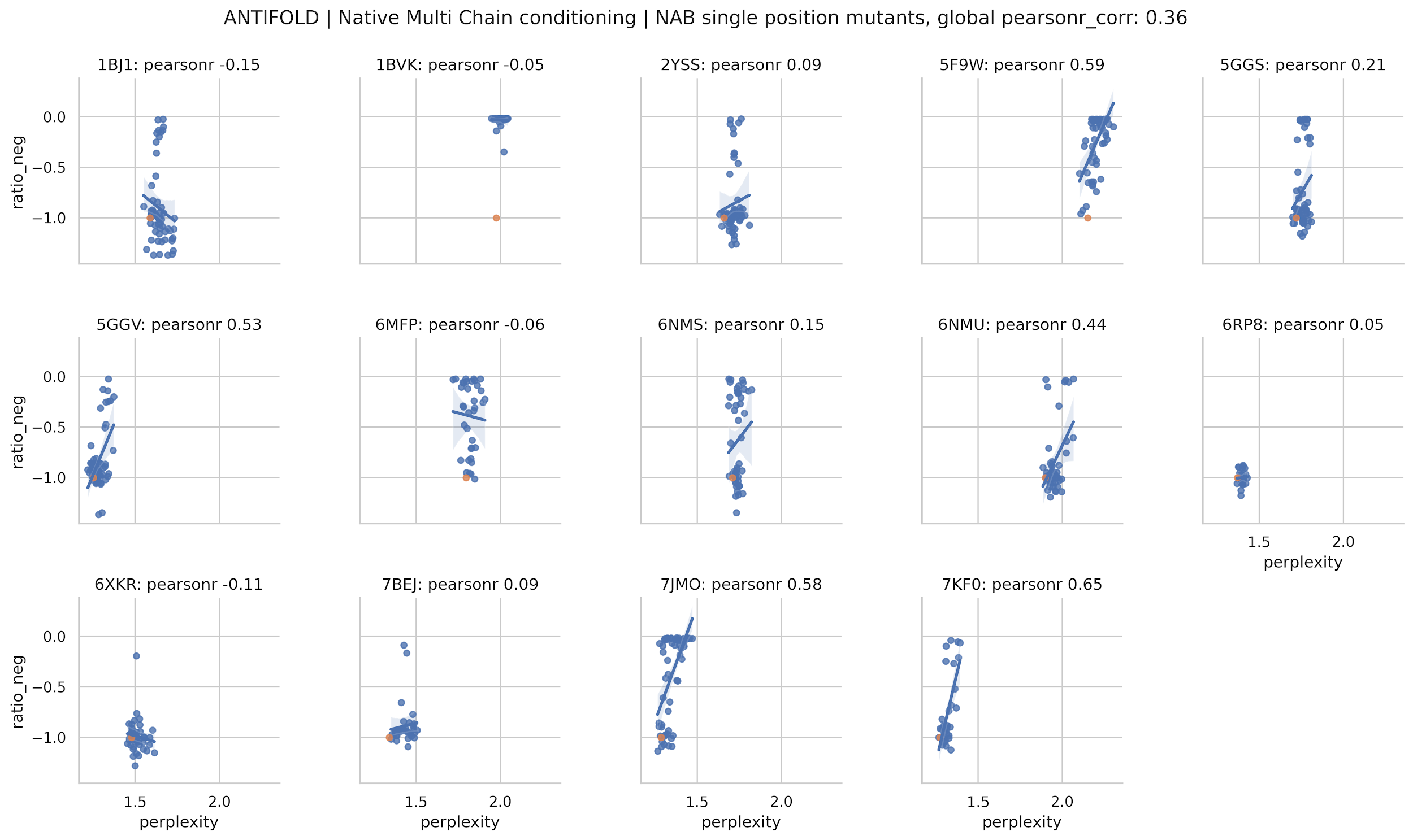
**Supplementary Figure 10. Antifold conditioned on native structure, with antigen.** Perplexity (x-axis) is plotted against the negative log ratio of the mutant/wt ELISA ratio (-1.0 is WT, closer to zero, worse binding).


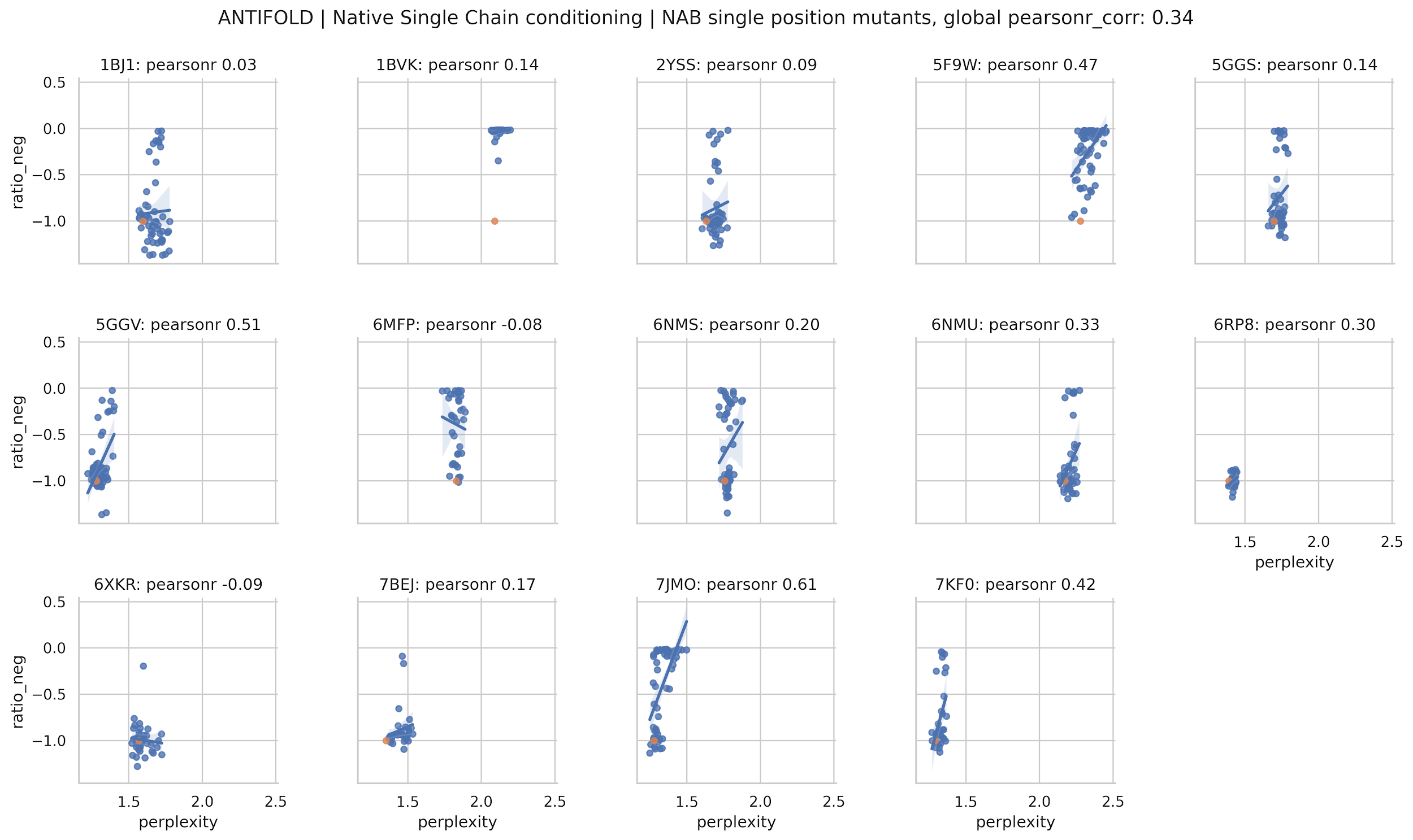
**Supplementary Figure 11. Antifold conditioned on native structure, no antigen.** Perplexity (x-axis) is plotted against the negative log ratio of the mutant/wt ELISA ratio (-1.0 is WT, closer to zero, worse binding).


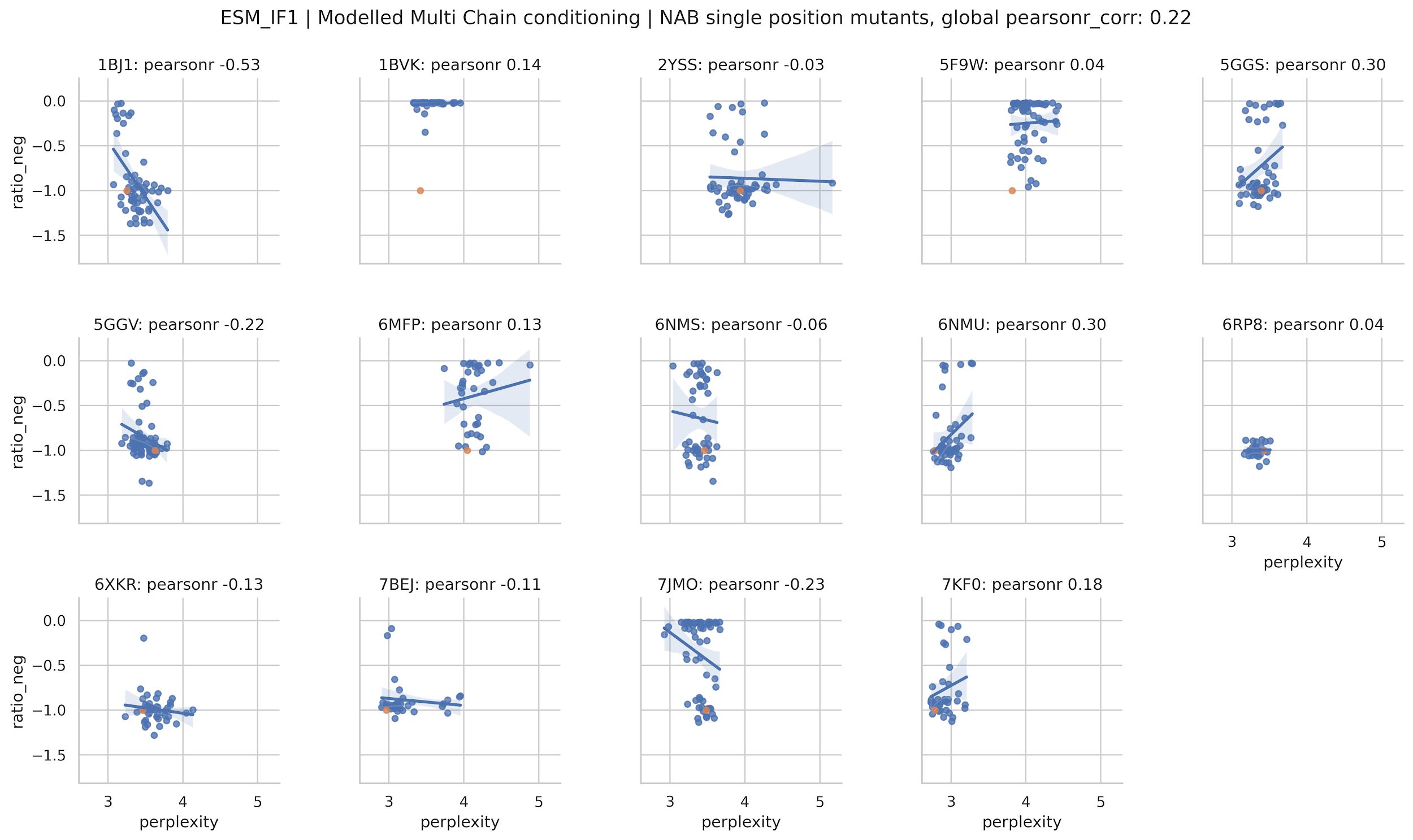
**Supplementary Figure 12. ESM-IF1 conditioned on native structure, with antigen.** Perplexity (x-axis) is plotted against the negative log ratio of the mutant/wt ELISA ratio (-1.0 is WT, closer to zero, worse binding).


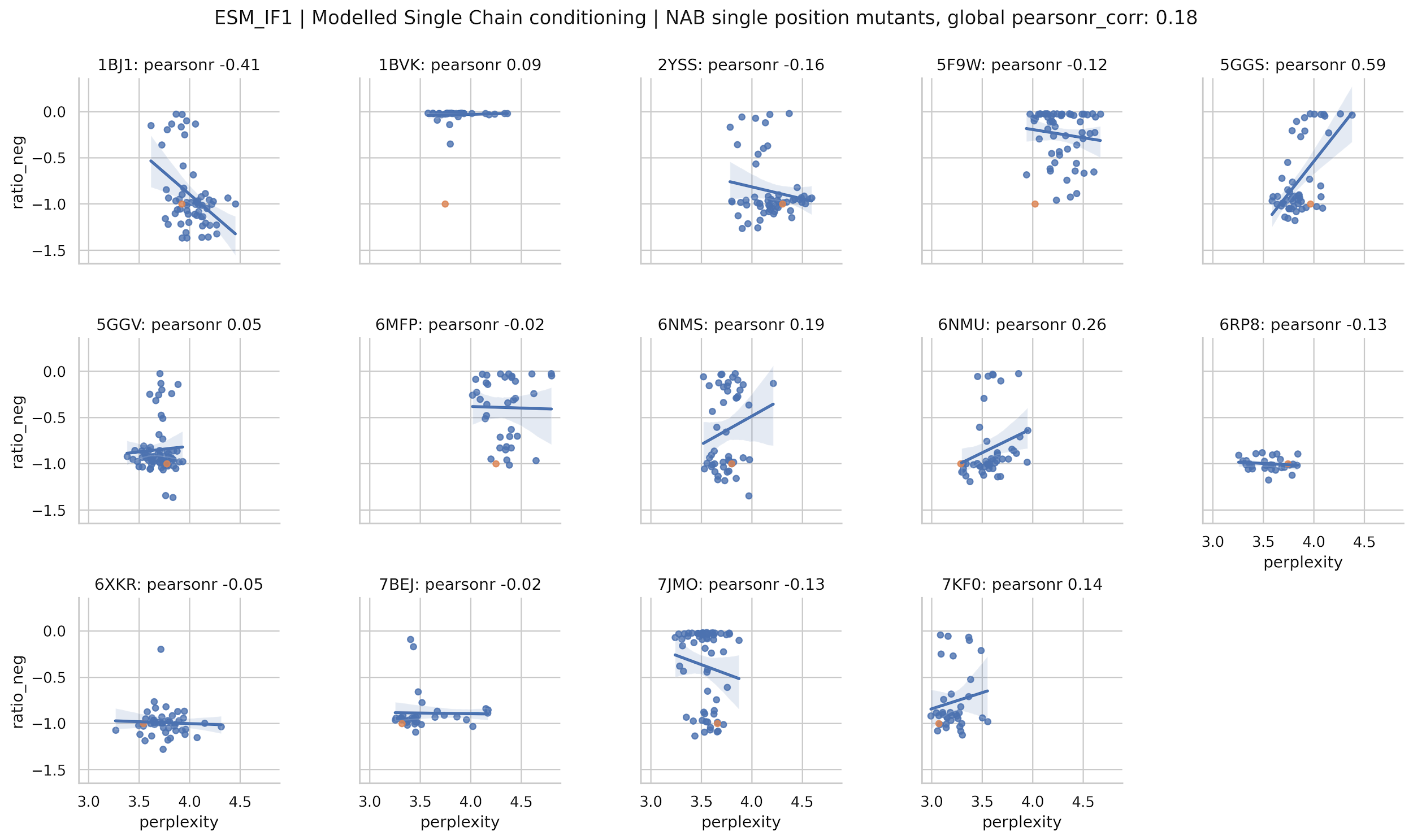


**Supplementary Figure 13. ESM-IF1 conditioned on modelled structure, no antigen.** Perplexity (x-axis) is plotted against the negative log ratio of the mutant/wt ELISA ratio (-1.0 is WT, closer to zero, worse binding).


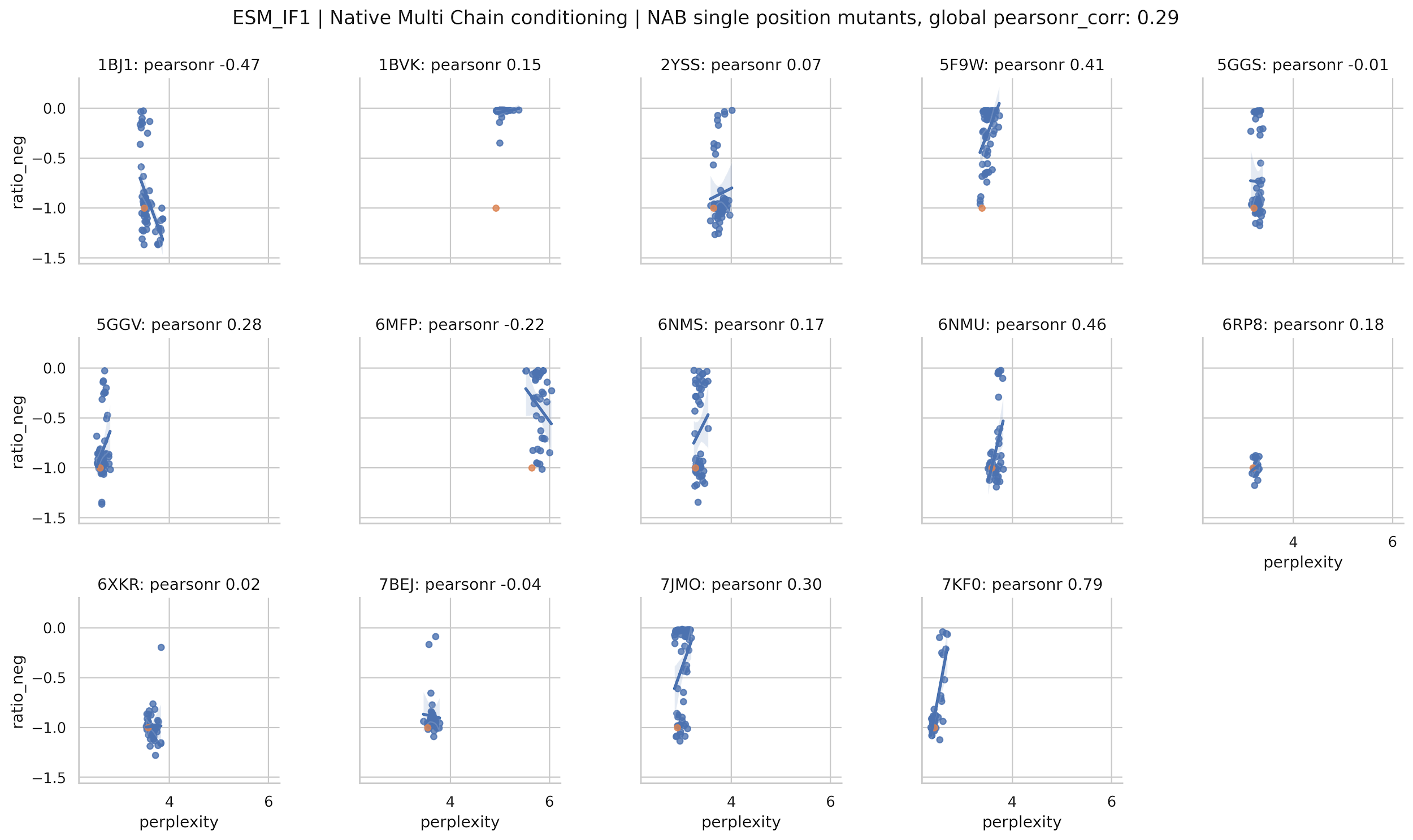


**Supplementary Figure 14. ESM-IF1 conditioned on native structure, with antigen.** Perplexity (x-axis) is plotted against the negative log ratio of the mutant/wt ELISA ratio (-1.0 is WT, closer to zero, worse binding).


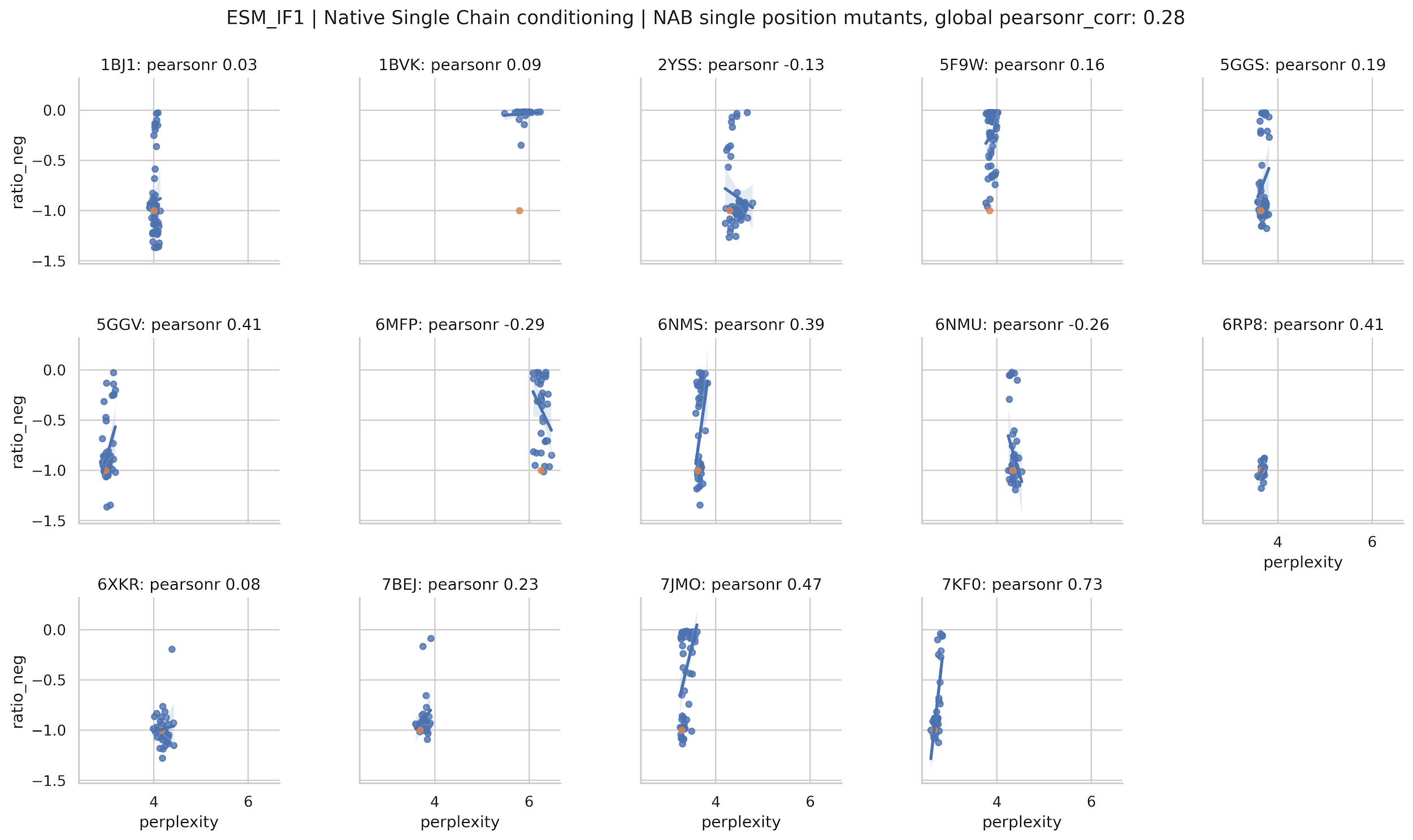


**Supplementary Figure 15. ESM-IF1 conditioned on native structure, no antigen.** Perplexity (x-axis) is plotted against the negative log ratio of the mutant/wt ELISA ratio (-1.0 is WT, closer to zero, worse binding).


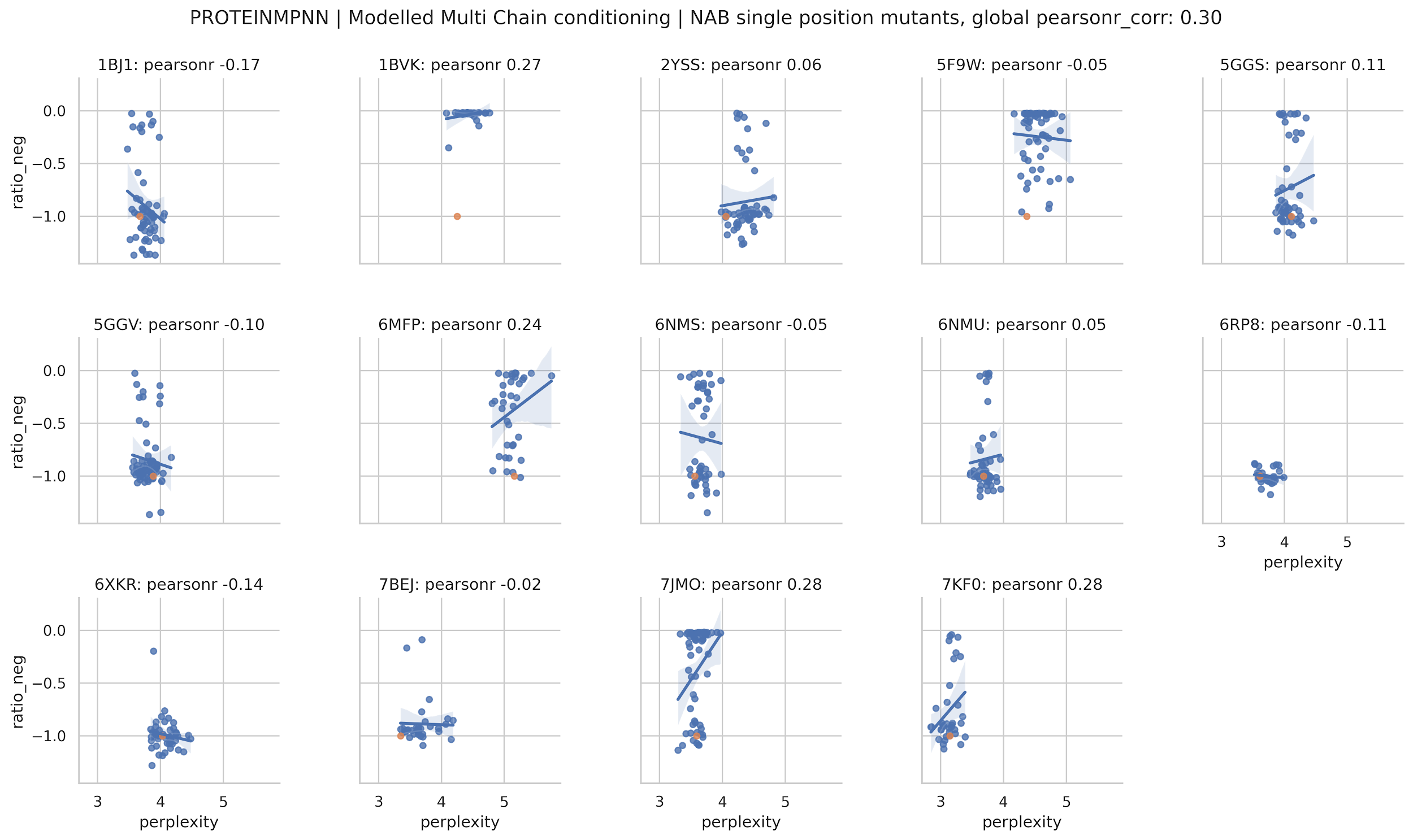


**Supplementary Figure 16. ProteinMPNN conditioned on modelled structure, with antigen.** Perplexity (x-axis) is plotted against the negative log ratio of the mutant/wt ELISA ratio (-1.0 is WT, closer to zero, worse binding).


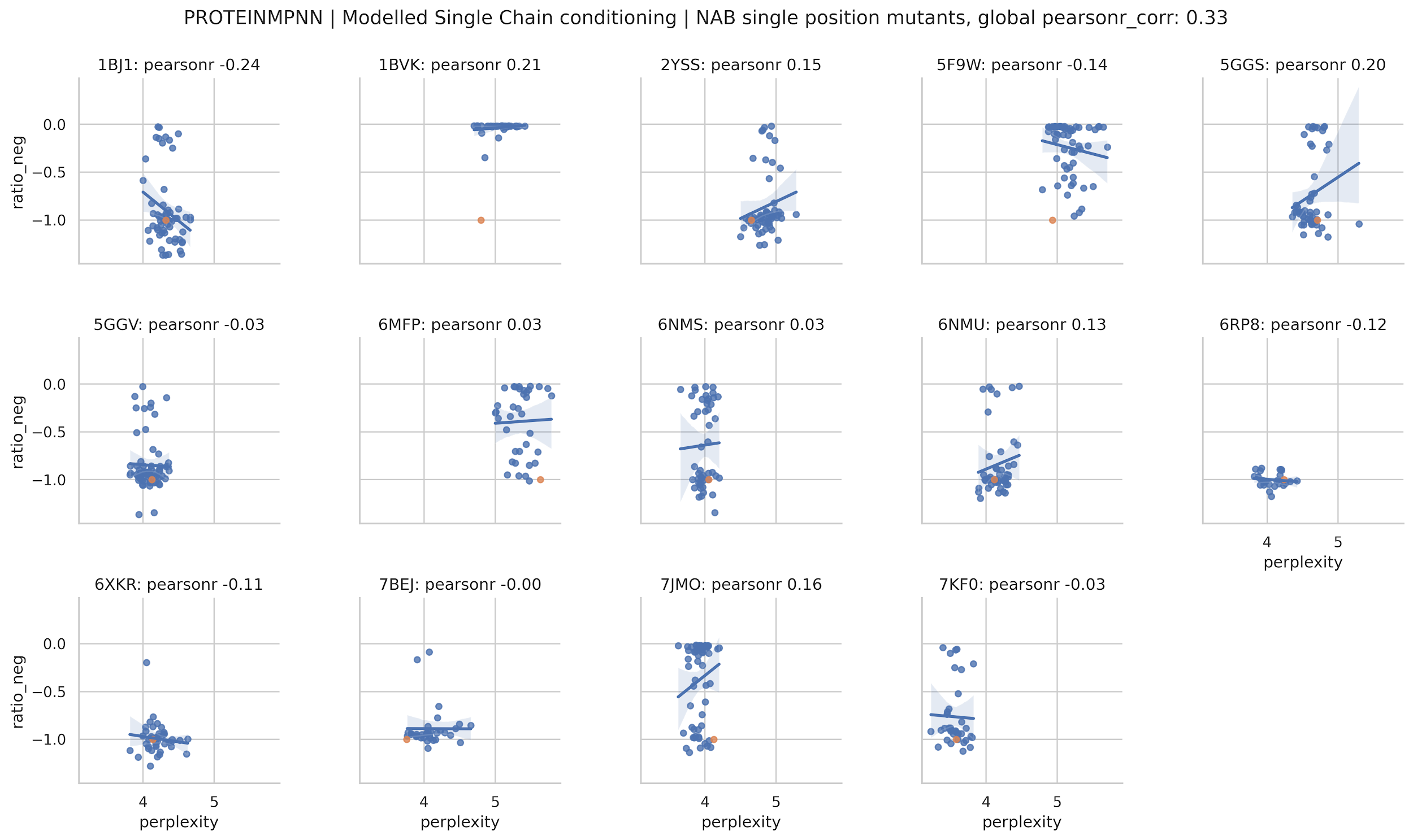
**Supplementary Figure 17. ProteinMPNN conditioned on modelled structure, no antigen.** Perplexity (x-axis) is plotted against the negative log ratio of the mutant/wt ELISA ratio (-1.0 is WT, closer to zero, worse binding).


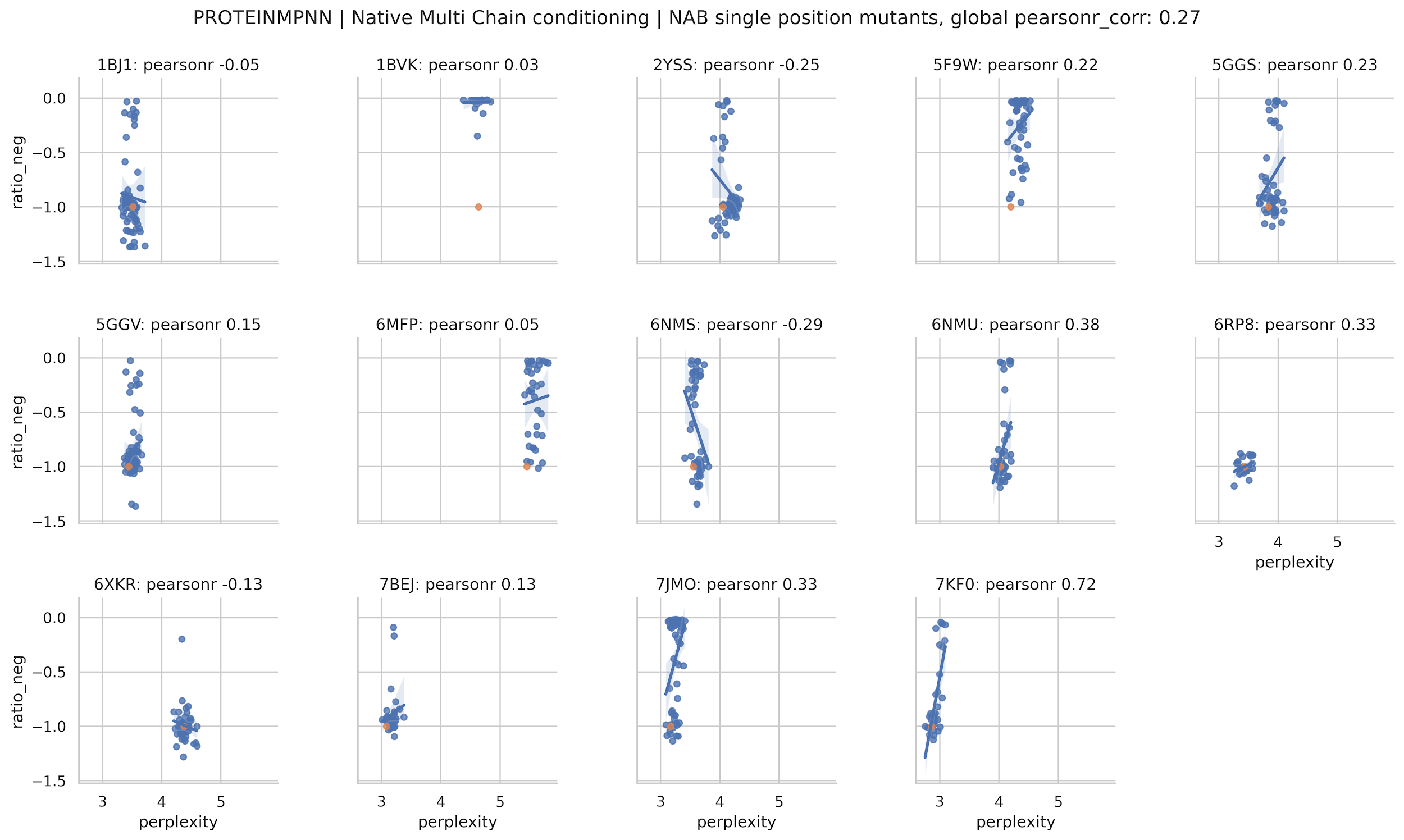


**Supplementary Figure 18. ProteinMPNN conditioned on native structure, with antigen.** Perplexity (x-axis) is plotted against the negative log ratio of the mutant/wt ELISA ratio (-1.0 is WT, closer to zero, worse binding).


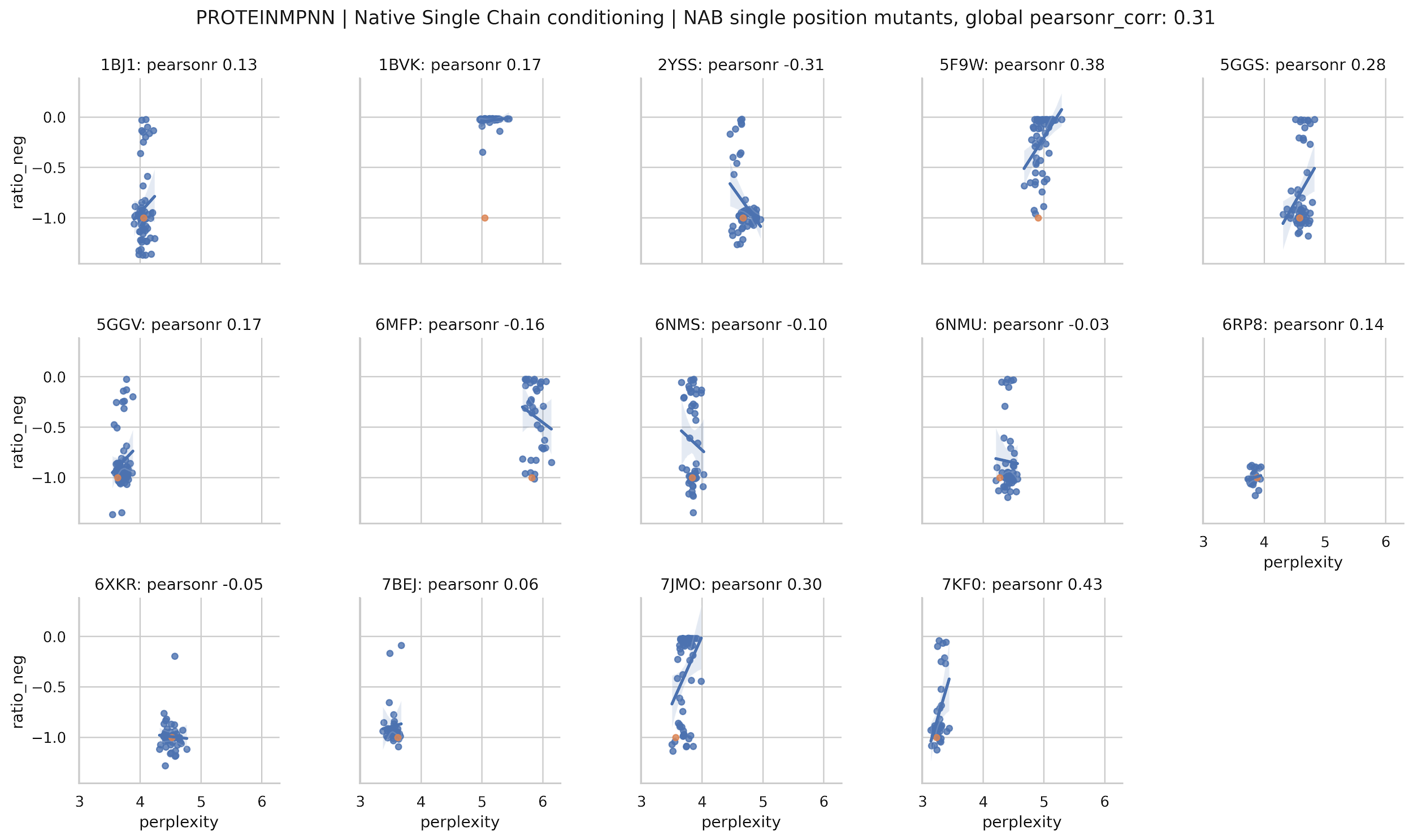


**Supplementary Figure 19. ProteinMPNN conditioned on native structure, no antigen.** Perplexity (x-axis) is plotted against the negative log ratio of the mutant/wt ELISA ratio (-1.0 is WT, closer to zero, worse binding).


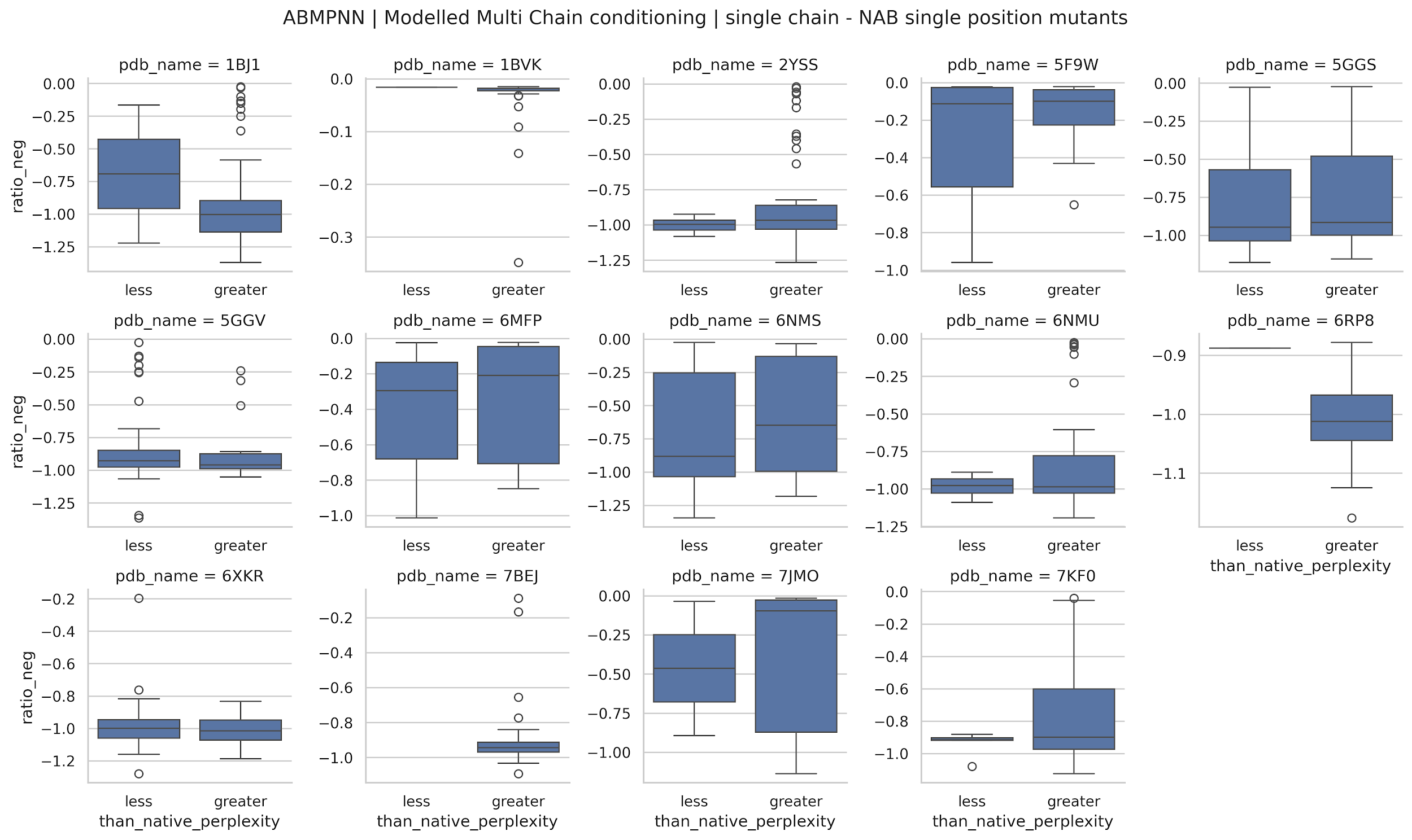
**Supplementary Figure 20. AbMPNN conditioned on modelled structure, with antigen. ‘**Less’ boxplots indicate perplexity scores smaller than Wild Type, whereas ‘greater’ indicate greater scores than wild type.


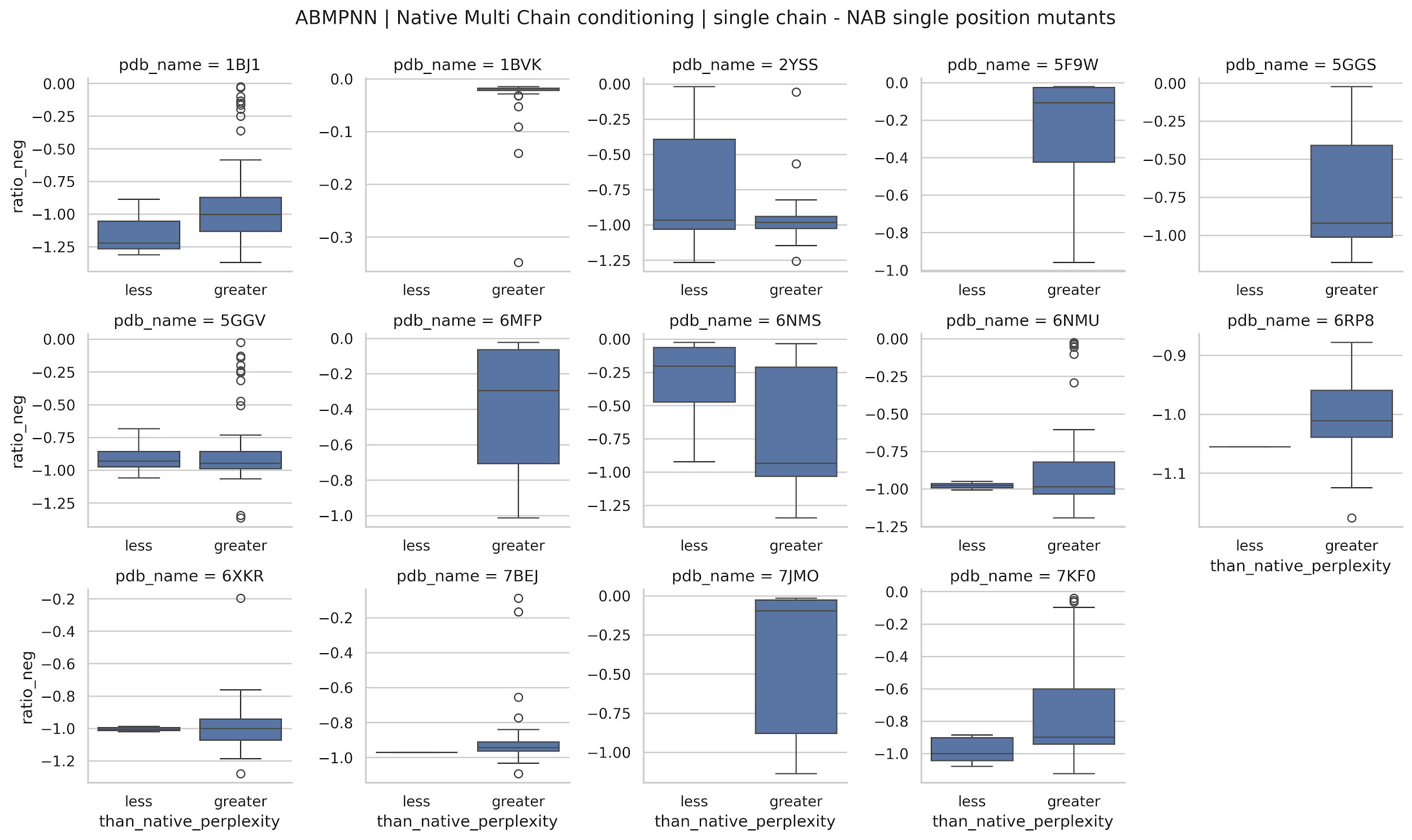
**Supplementary Figure 21. AbMPNN conditioned on native structure, with antigen. ‘**Less’ boxplots indicate perplexity scores smaller than Wild Type, whereas ‘greater’ indicate greater scores than wild type.


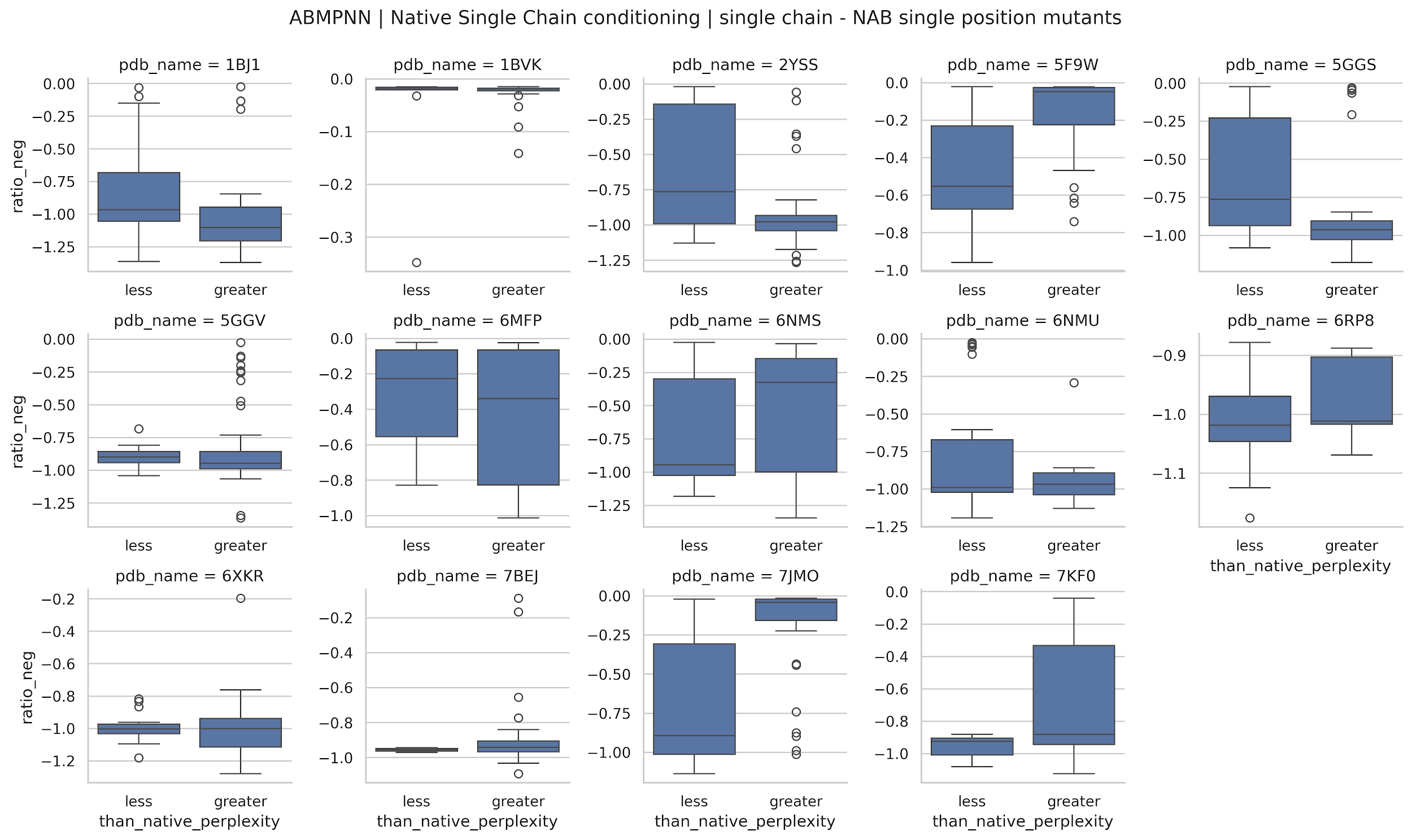
**Supplementary Figure 22. AbMPNN conditioned on native structure, no antigen. ‘**Less’ boxplots indicate perplexity scores smaller than Wild Type, whereas ‘greater’ indicate greater scores than wild type.
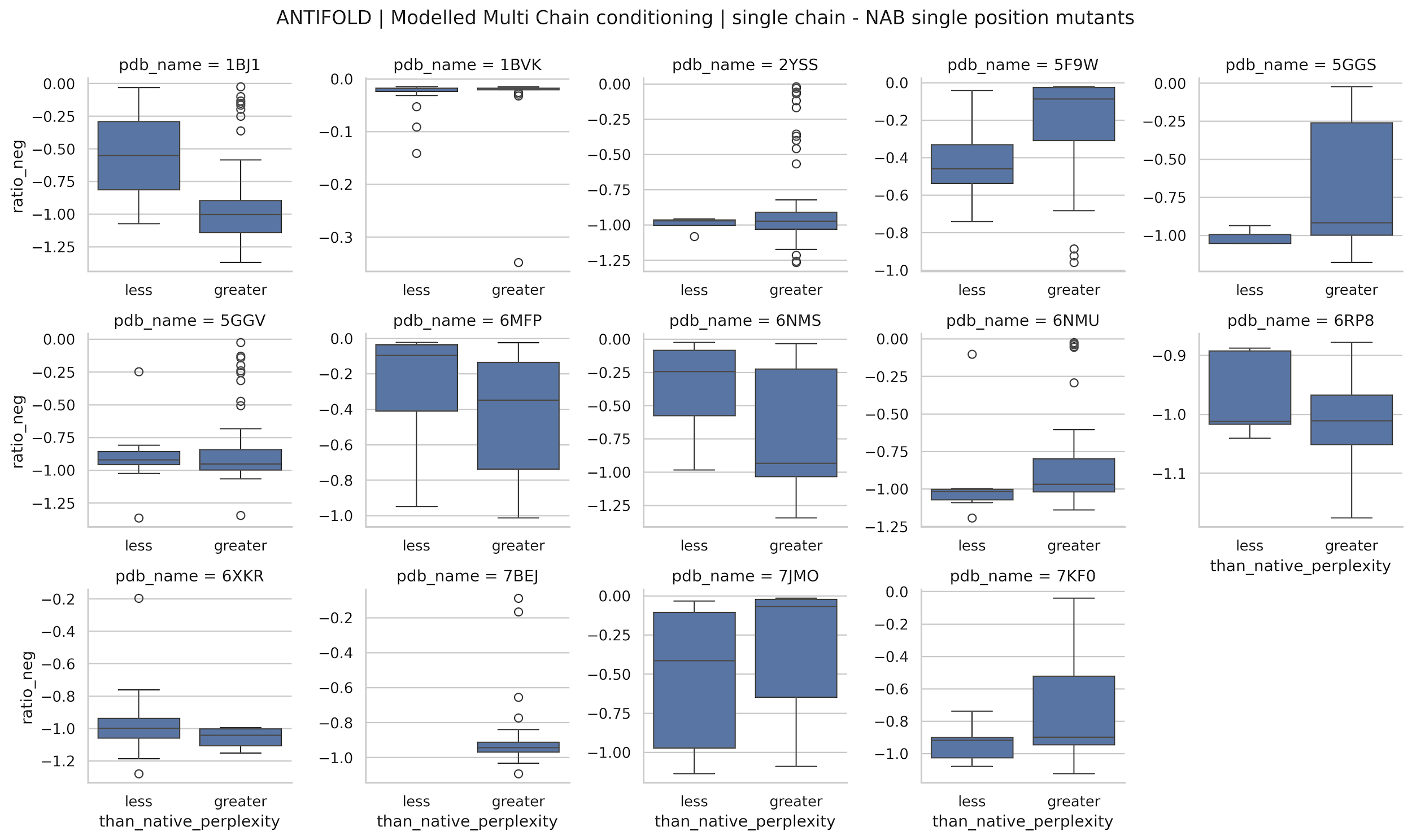
**Supplementary Figure 23. Antifold conditioned on modelled structure, with antigen. ‘**Less’ boxplots indicate perplexity scores smaller than Wild Type, whereas ‘greater’ indicate greater scores than wild type.
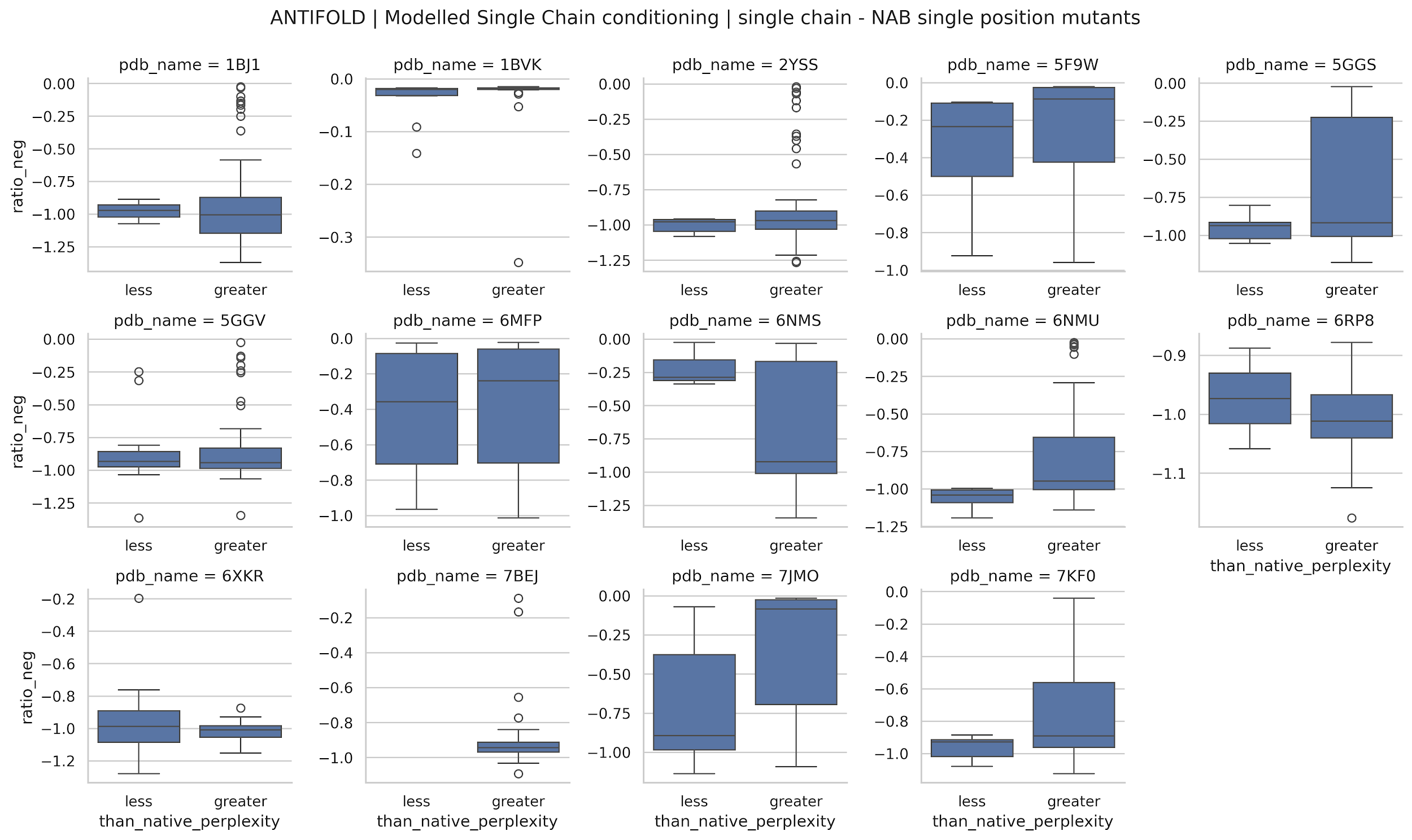
**Supplementary Figure 24. Antifold conditioned on modelled structure, no antigen. ‘**Less’ boxplots indicate perplexity scores smaller than Wild Type, whereas ‘greater’ indicate greater scores than wild type.


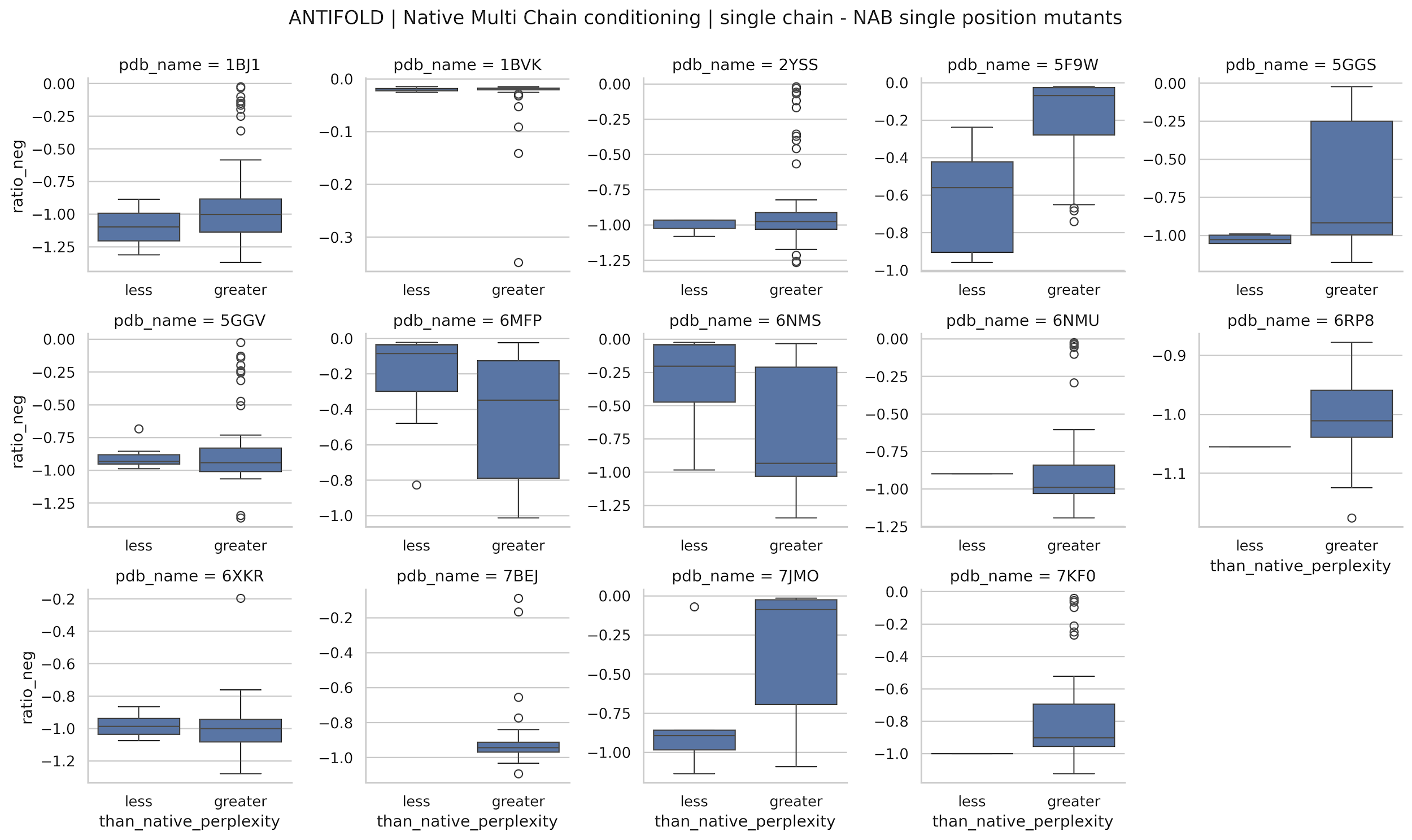
**Supplementary Figure 25. Antifold conditioned on native structure, with antigen. ‘**Less’ boxplots indicate perplexity scores smaller than Wild Type, whereas ‘greater’ indicate greater scores than wild type.


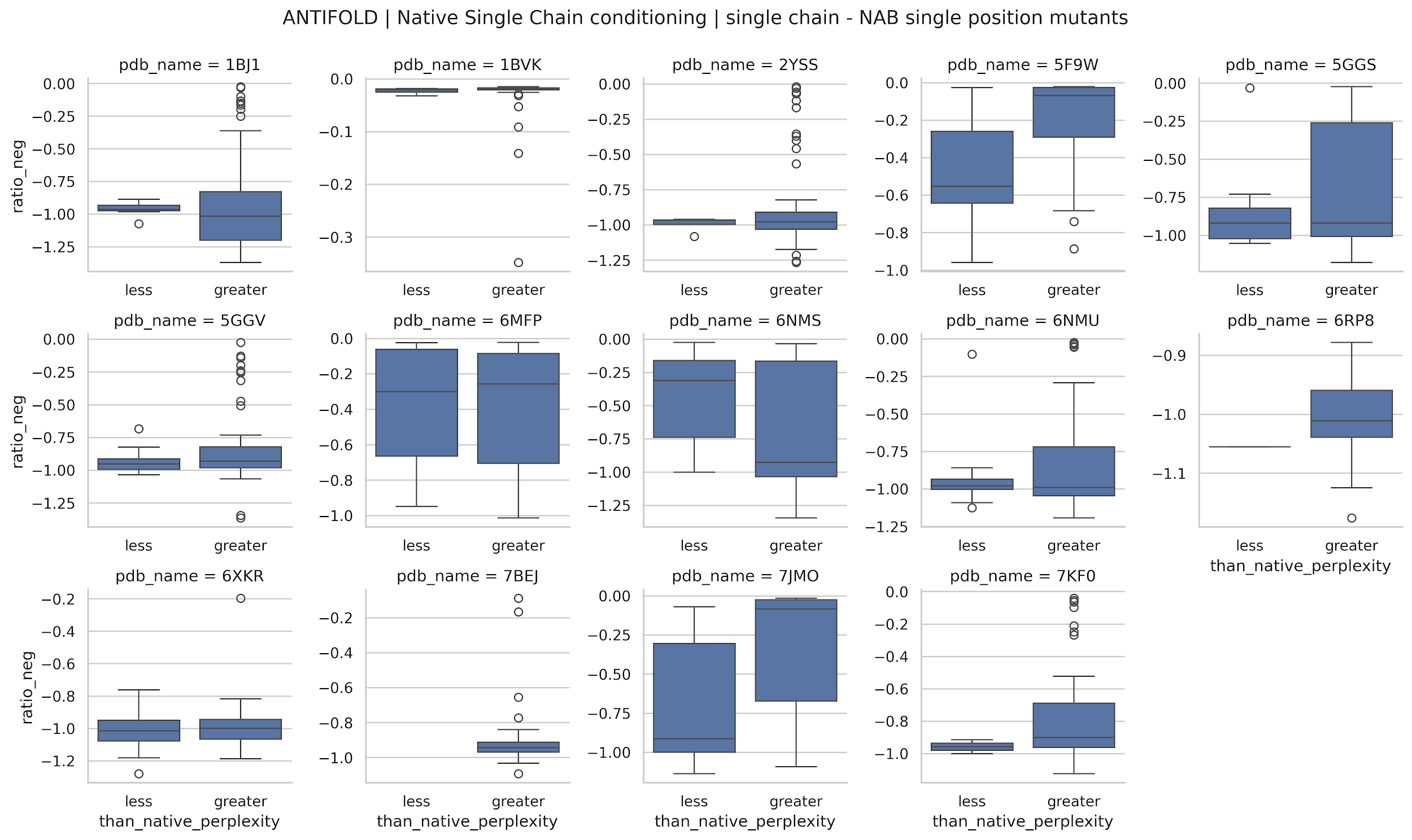
**Supplementary Figure 26. Antifold conditioned on native structure, no antigen. ‘**Less’ boxplots indicate perplexity scores smaller than Wild Type, whereas ‘greater’ indicate greater scores than wild type.


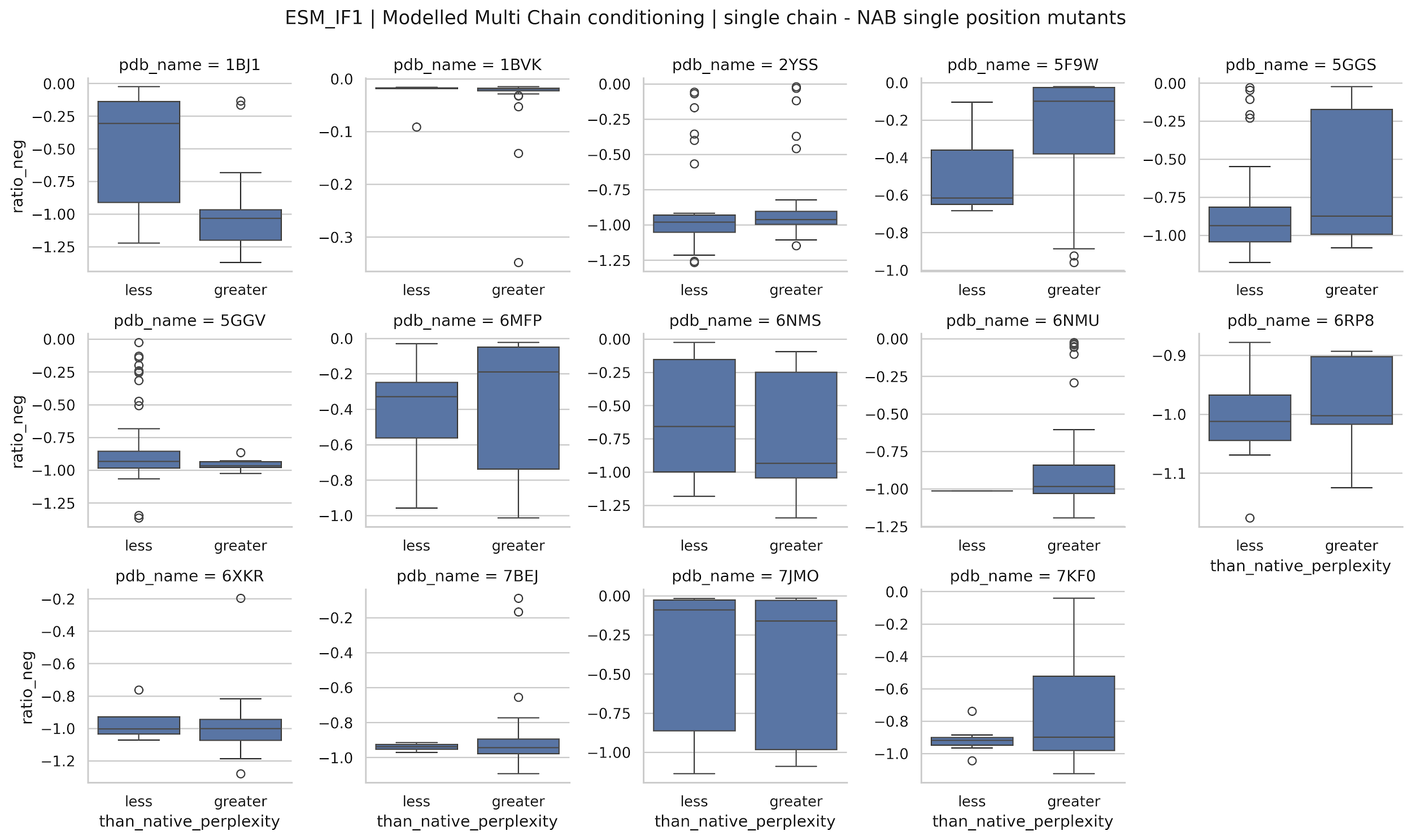
**Supplementary Figure 27. ESM-IF1 conditioned on modelled structure, with antigen. ‘**Less’ boxplots indicate perplexity scores smaller than Wild Type, whereas ‘greater’ indicate greater scores than wild type.


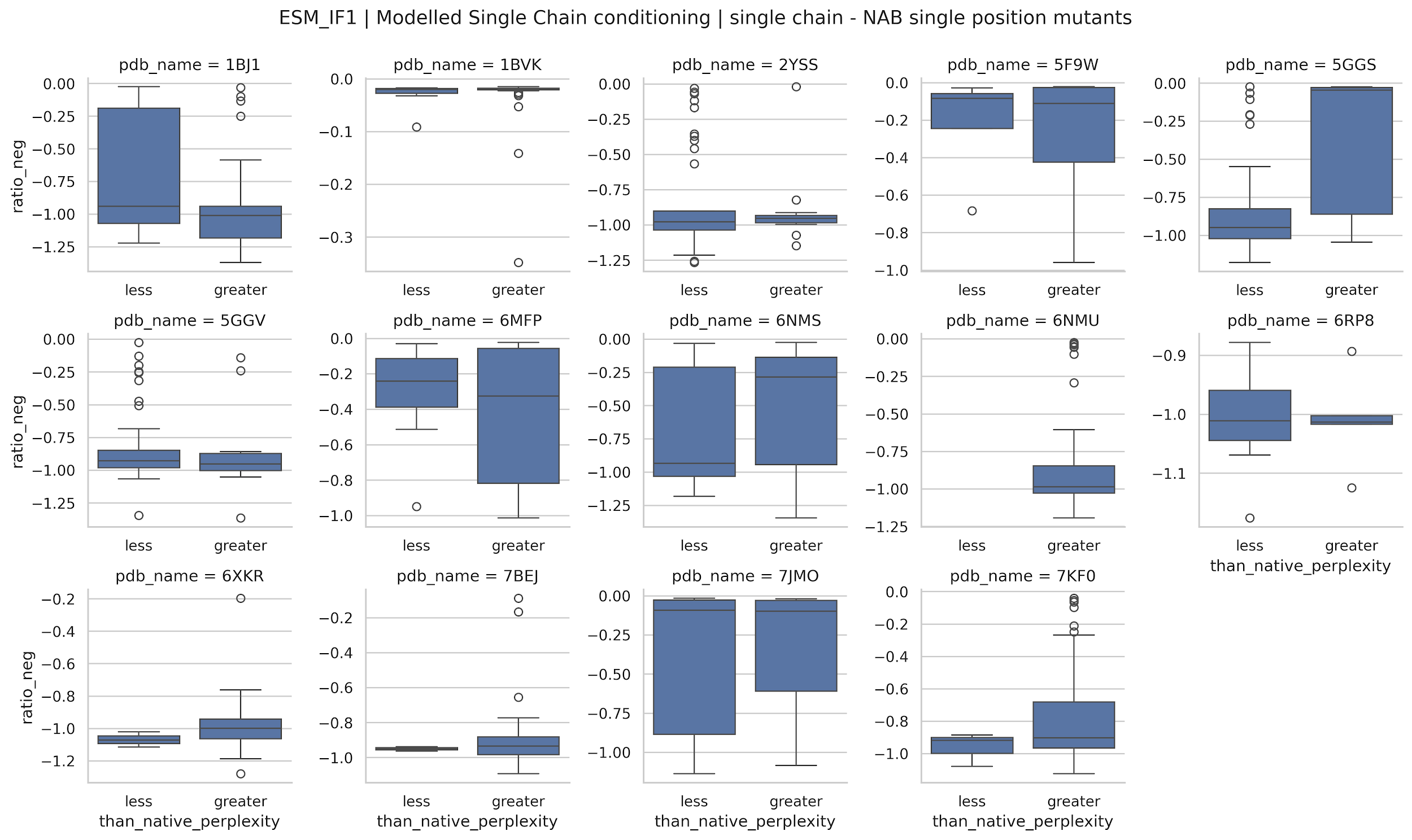


**Supplementary Figure 28. ESM-IF1 conditioned on modelled structure, no antigen. ‘**Less’ boxplots indicate perplexity scores smaller than Wild Type, whereas ‘greater’ indicate greater scores than wild type.


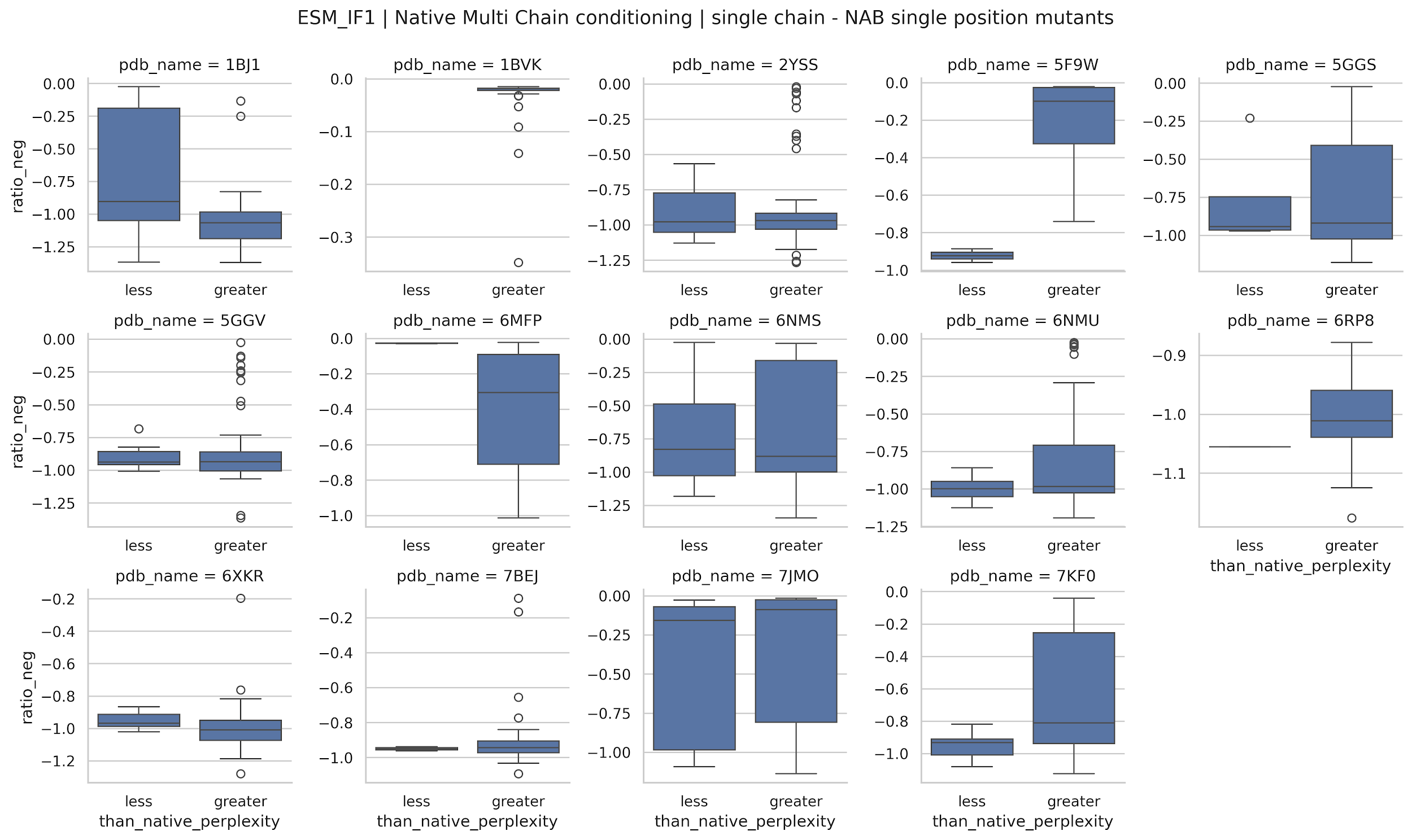
**Supplementary Figure 29. ESM-IF1 conditioned on native structure, with antigen. ‘**Less’ boxplots indicate perplexity scores smaller than Wild Type, whereas ‘greater’ indicate greater scores than wild type.


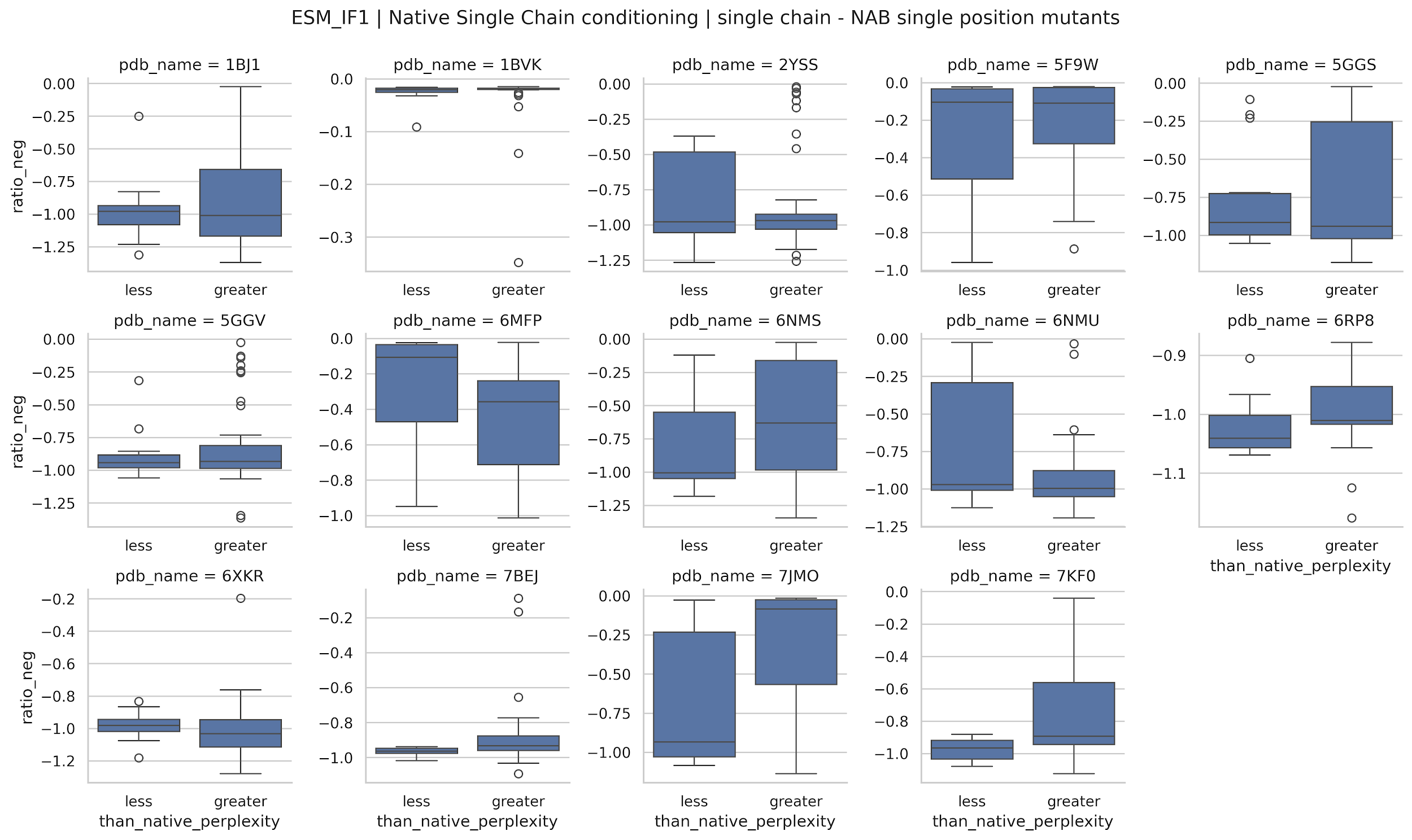
**Supplementary Figure 30. ESM-IF1 conditioned on native structure, no antigen. ‘**Less’ boxplots indicate perplexity scores smaller than Wild Type, whereas ‘greater’ indicate greater scores than wild type.


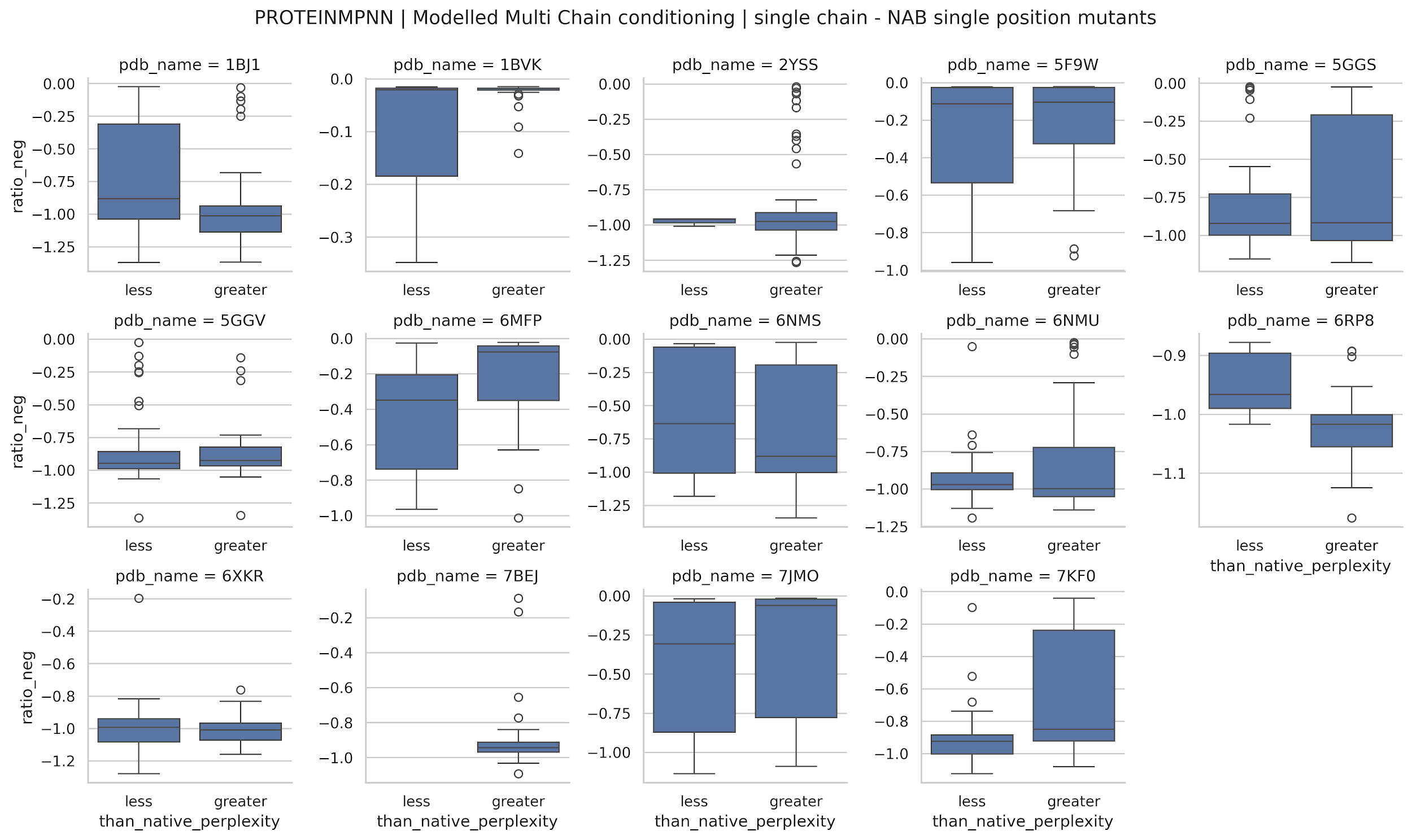
**Supplementary Figure 31. ProteinMPNN conditioned on modelled structure, with antigen. ‘**Less’ boxplots indicate perplexity scores smaller than Wild Type, whereas ‘greater’ indicate greater scores than wild type.


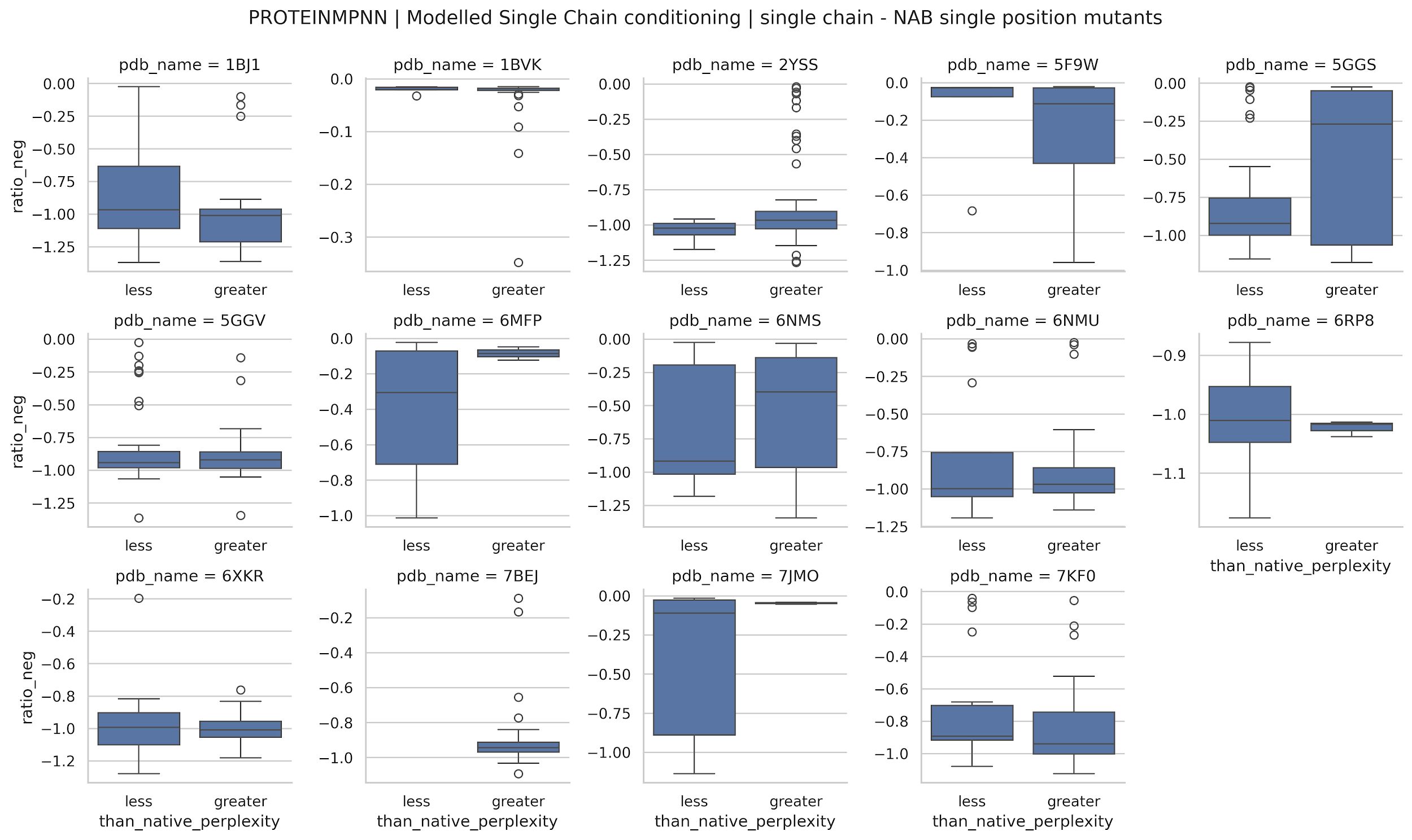
**Supplementary Figure 32. ProteinMPNN conditioned on modelled structure, no antigen. ‘**Less’ boxplots indicate perplexity scores smaller than Wild Type, whereas ‘greater’ indicate greater scores than wild type.
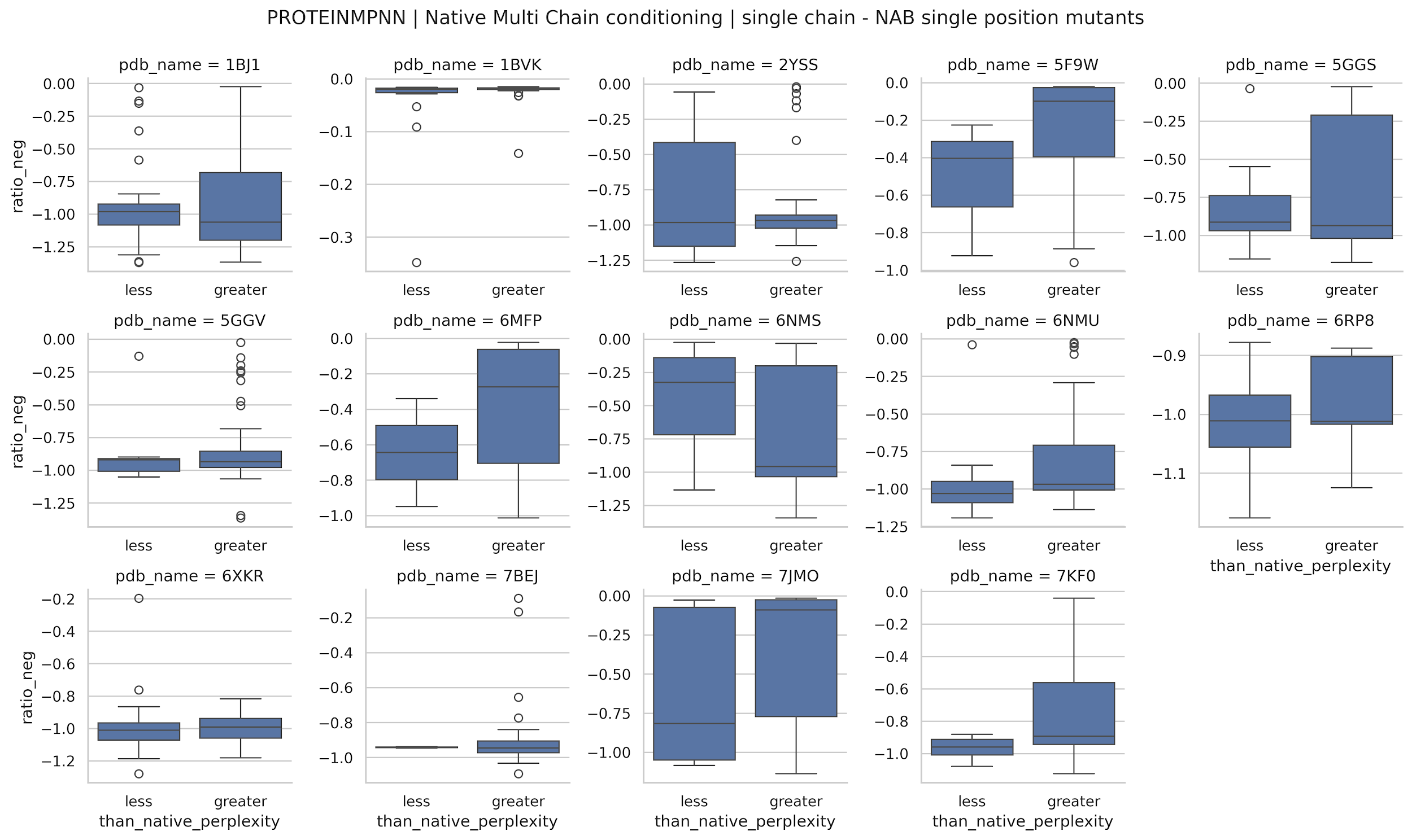
**Supplementary Figure 33. ProteinMPNN conditioned on native structure, with antigen. ‘**Less’ boxplots indicate perplexity scores smaller than Wild Type, whereas ‘greater’ indicate greater scores than wild type.


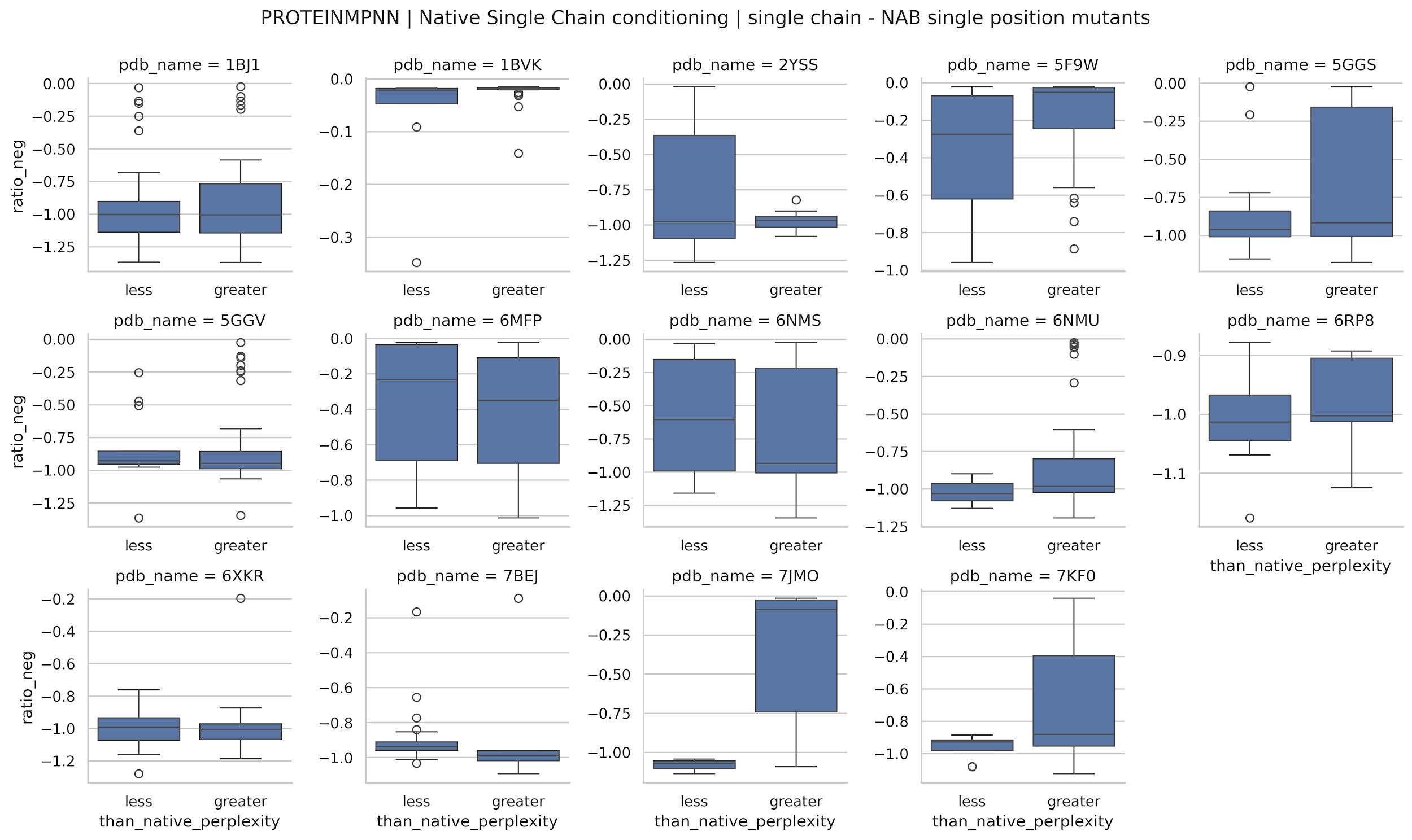
**Supplementary Figure 34. ProteinMPNN conditioned on native structure, no antigen. ‘**Less’ boxplots indicate perplexity scores smaller than Wild Type, whereas ‘greater’ indicate greater scores than wild type.


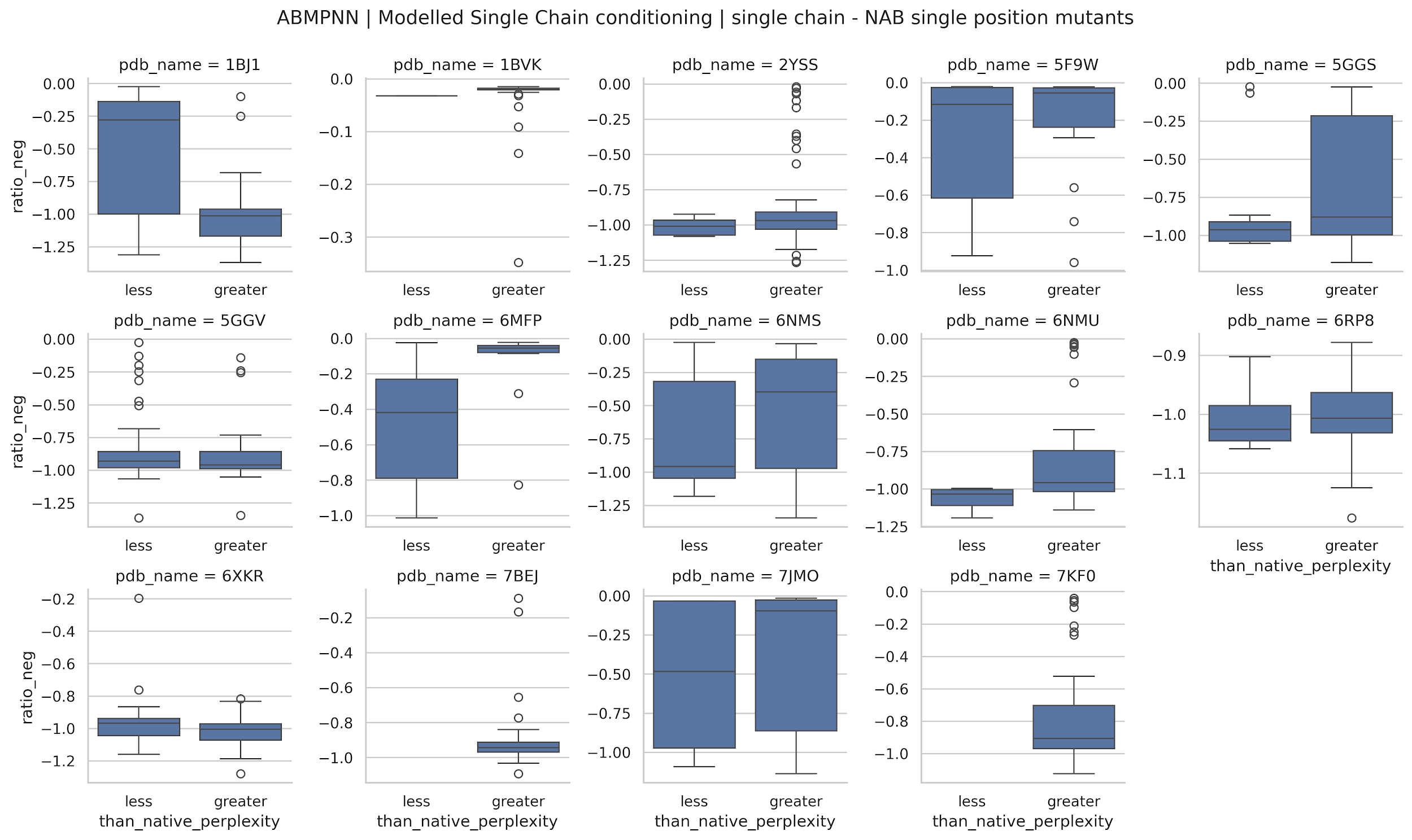


**Supplementary Figure 35. ProteinMPNN conditioned on modelled structure, no antigen. ‘**Less’ boxplots indicate perplexity scores smaller than Wild Type, whereas ‘greater’ indicate greater scores than wild type.


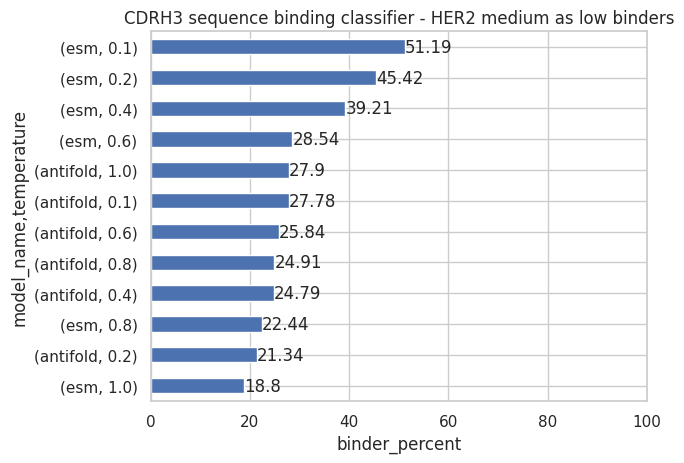

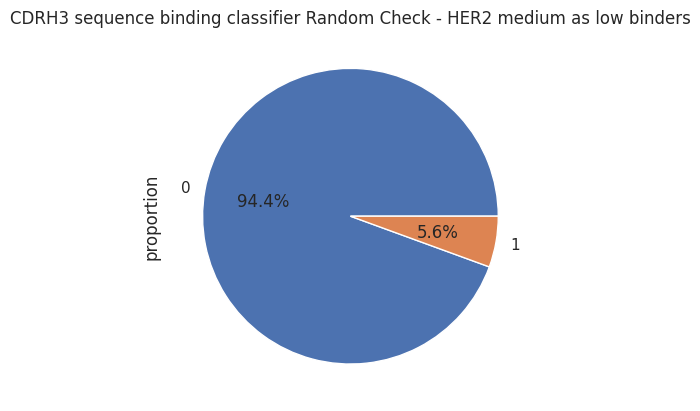


**Supplementary Figure 20: ESM-IF and Antifold sampled CDRH3 classification.** (Left) ESM-IF and Antifold sampled sequences classification by CNN model trained on CDRH3 sequences from HER2 dataset with medium labeled as low binders. Each bar corresponds to sequences generated using a given IF model on specific temperature setting. (Right) CDRH3 classifier sanity check - proportions of binder / non-binder classification on randomly generated CDH3 dataset.


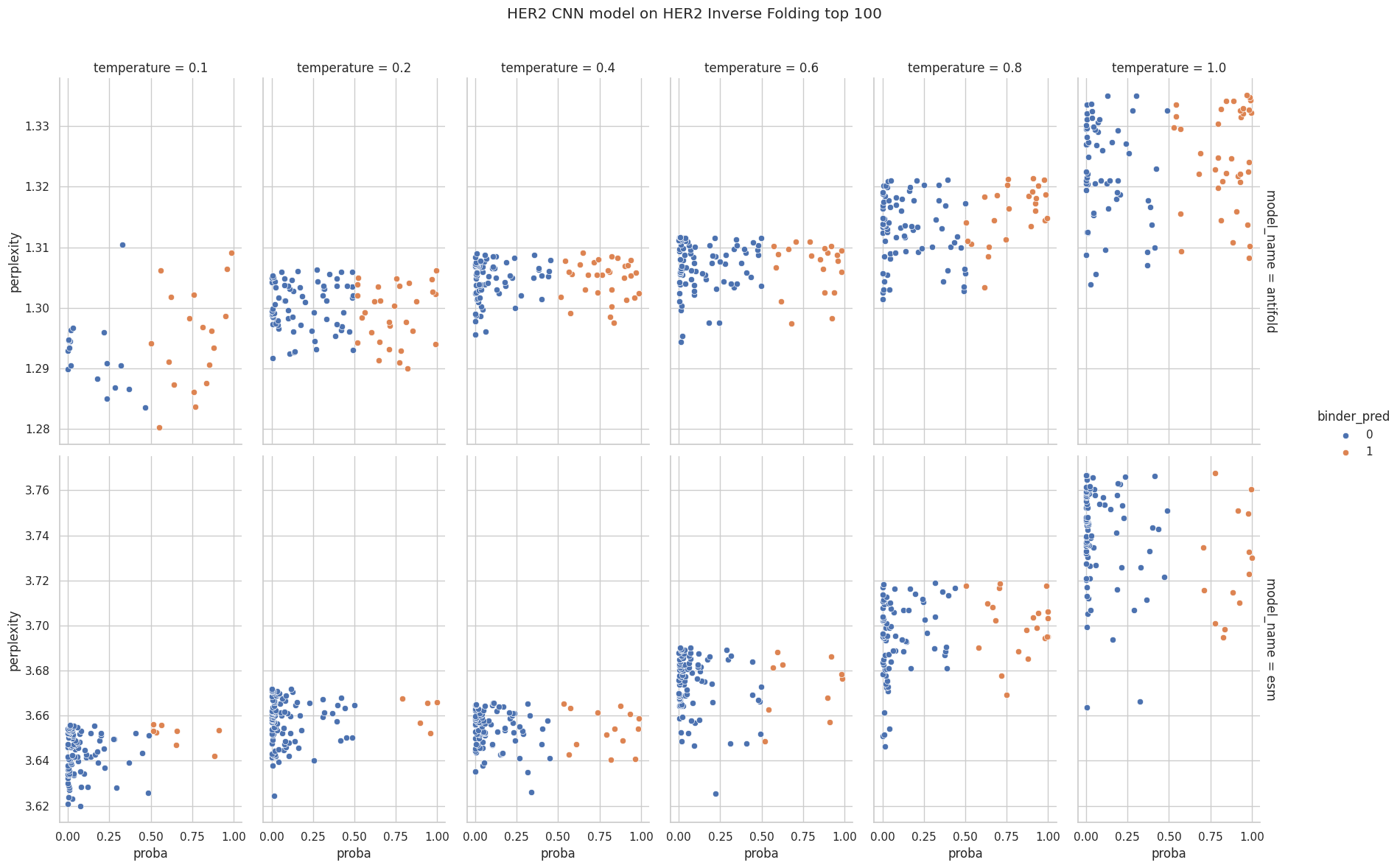


**Supplementary Figure 21: Perplexity distribution chart for ESM-IF and Antifold** - sampled sequences were scored using the same model they were generated with. Top 100 sequences for every model-temperature are displayed. Observations are colored by oracle classification CNN model trained on HER2 dataset with medium labeled as low binders.


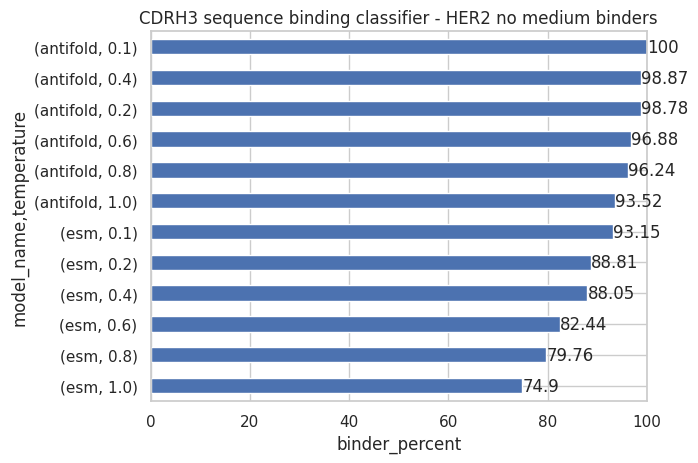

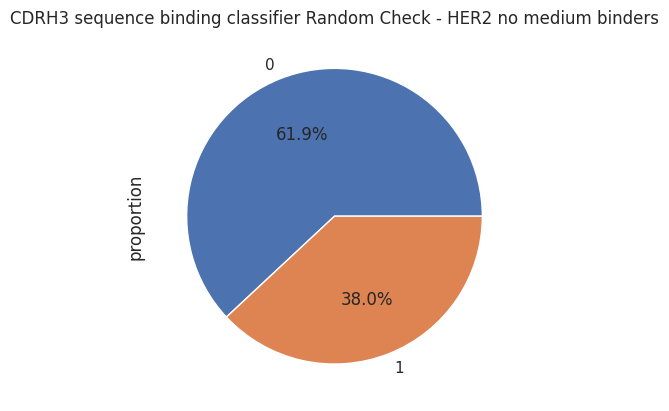


**Supplementary Figure 22: ESM-IF and Antifold sampled CDRH3 classification.** (Left) ESM-IF and Antifold sampled sequences classification by CNN model trained on CDRH3 sequences from HER2 dataset with removed medium binders. Each bar corresponds to sequences generated using a given IF model on specific temperature setting. (Right) CDRH3 classifier sanity check - proportions of binder / non-binder classification on randomly generated CDH3 dataset.


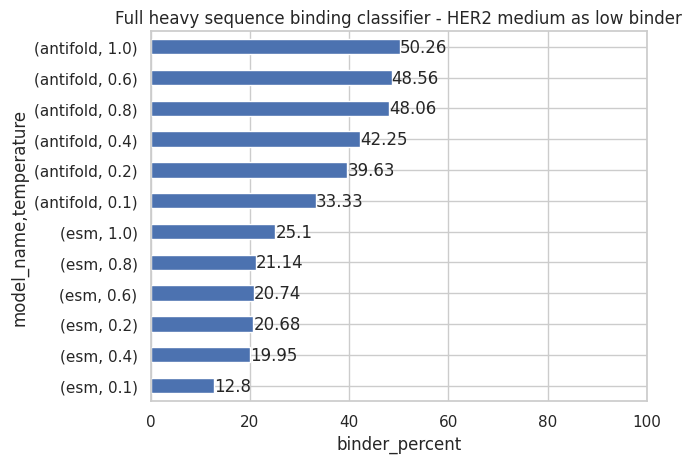

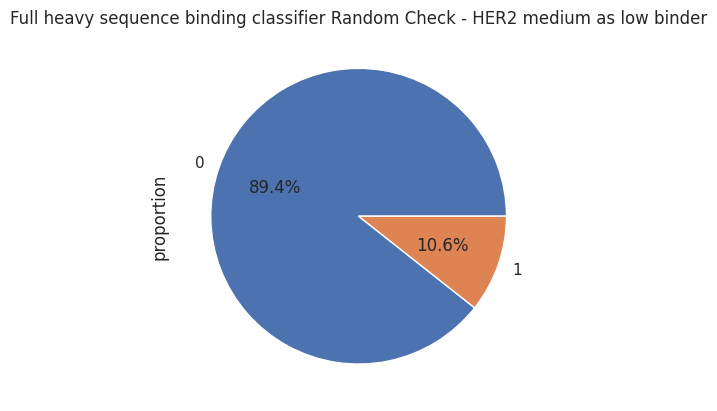


**Supplementary Figure 23: ESM-IF and Antifold sampled full sequence classification.** (Left) ESM-IF and Antifold sampled sequences classification by CNN model trained on full heavy HER2 dataset with medium labeled as low binders. Each bar corresponds to sequences generated using a given IF model on specific temperature settings. (Right) Full heavy sequence classifier sanity check - proportions of binder / non-binder classification on randomly generated CDH3 dataset.


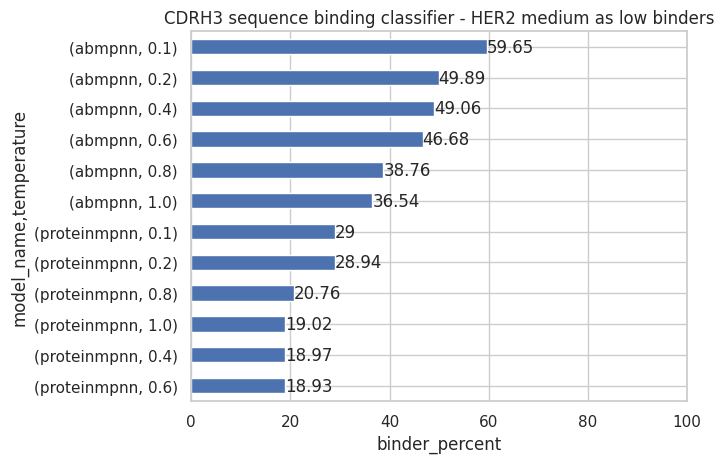

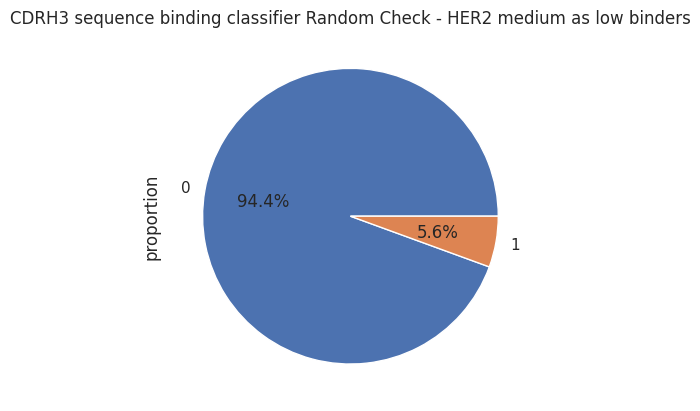


**Supplementary Figure 24: ProteinMPNN and AbMPNN sampled CDRH3 classification.** (Left) ProteinMPNN and AbMPNN sampled sequence classification by CNN model trained on CDRH3 sequences from HER2 dataset with medium labeled as low binders. Each bar corresponds to sequences generated using a given IF model on specific temperature setting. (Right) CDRH3 classifier sanity check - proportions of binder / non-binder classification on randomly generated CDH3 dataset.


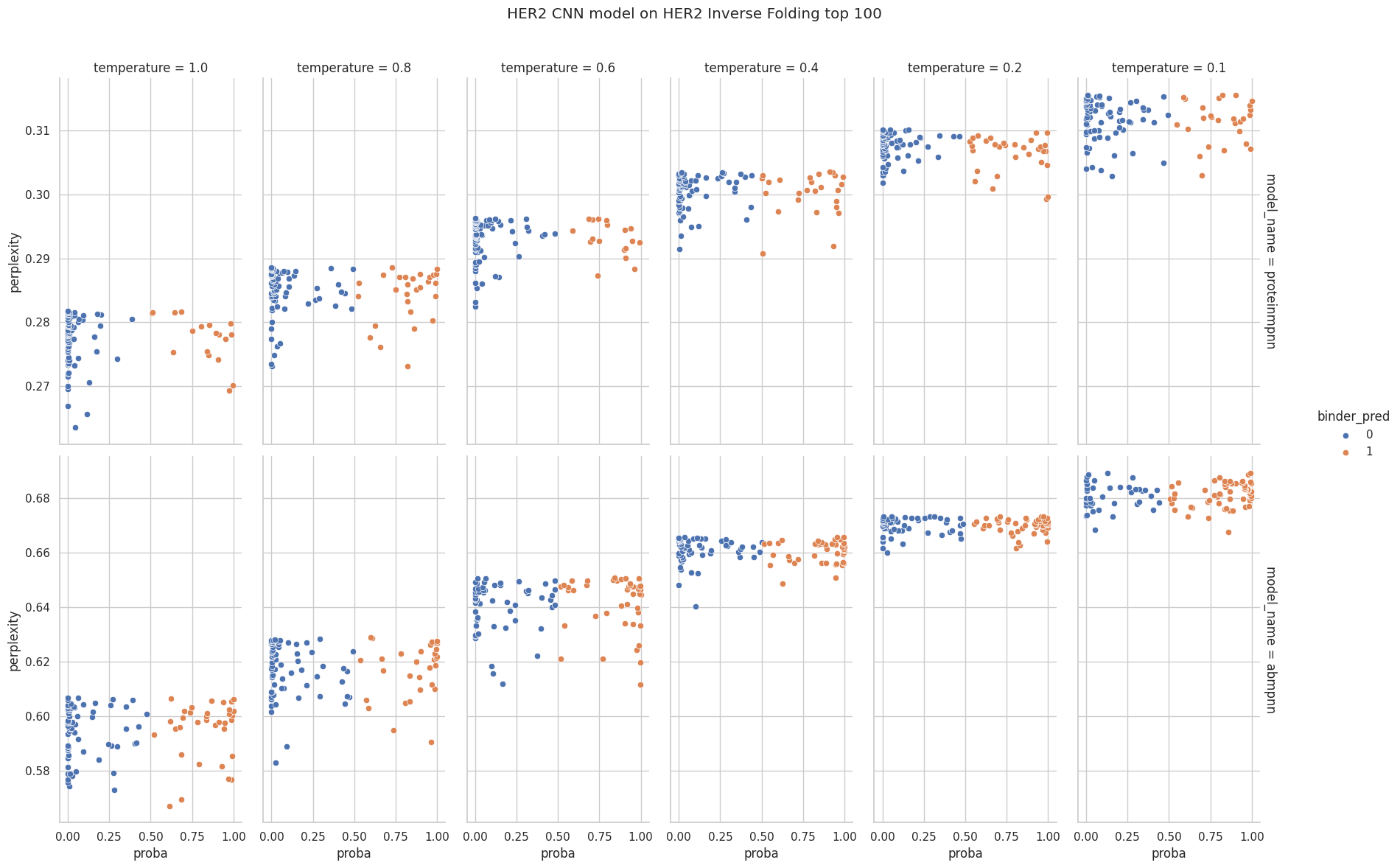
**Supplementary Figure 25: Perplexity distribution chart for ProteinMPNN and AbMPNN.** Sampled sequences were scored using the same model they were generated with. Top 100 sequences for every model-temperature are displayed. Observations are colored by oracle classification CNN model trained on HER2 dataset with medium labeled as low binders.


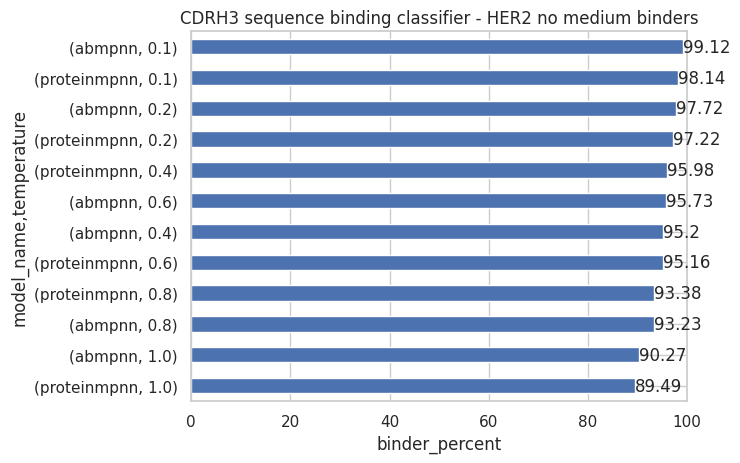

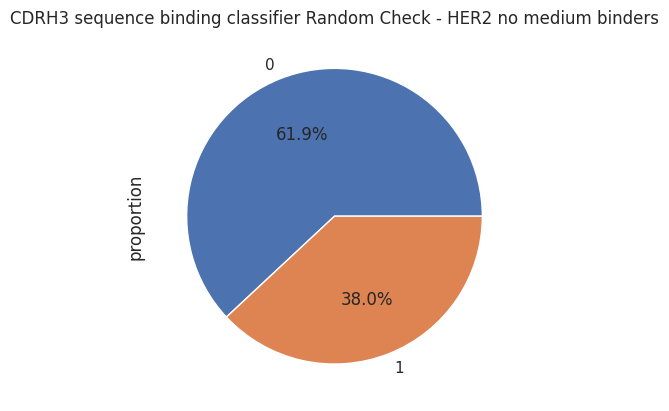


**Supplementary Figure 26: ProteinMPNN and AbMPNN sampled CDRH3 classification.** (Left) ProteinMPNN and AbMPNN sampled sequences classification by CNN model trained on CDRH3 sequences from HER2 dataset with removed medium binders. Each bar corresponds to sequences generated using a given IF model on specific temperature setting. (Right) CDRH3 classifier sanity check - proportions of binder / non-binder classification on randomly generated CDH3 dataset.

####
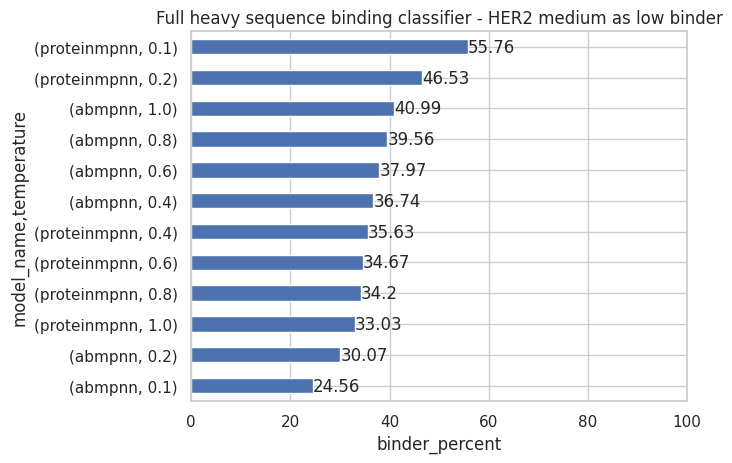

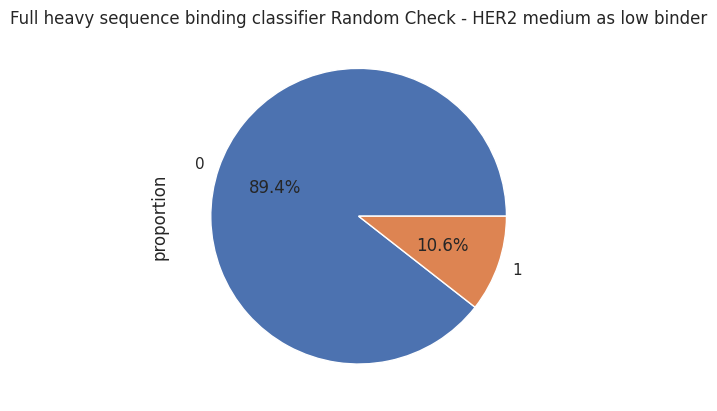


**Supplementary Figure 27: ProteinMPNN and AbMPNN sampled full sequence classification.** (Left) ProteinMPNN and AbMPNN sampled sequences classification by CNN model trained on full heavy HER2 dataset with medium labeled as low binders. Each bar corresponds to sequences generated using a given IF model on specific temperature setting. (Right) Full heavy sequence classifier sanity check - proportions of binder / non-binder classification on randomly generated CDH3 dataset.


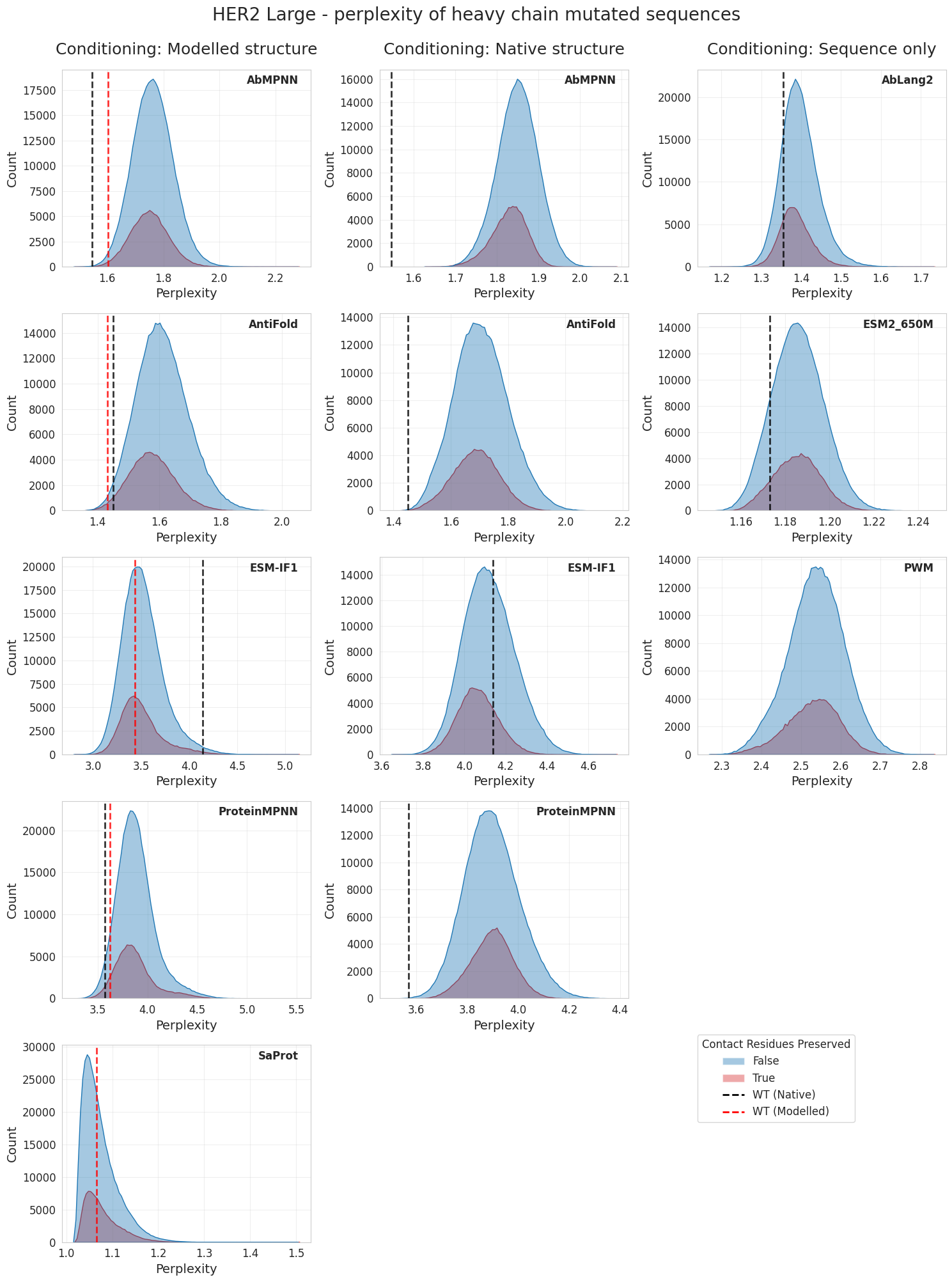


**Supplementary Figure 28: Perplexity distributions for Trastuzumab mutants with contact residues preservation and without**

For any benchmarked model we did not find any significant influence on perplexity score if Trastuzumab’ WT contact residues (IMGT 107W, 111G and 113Y) were left unchanged.


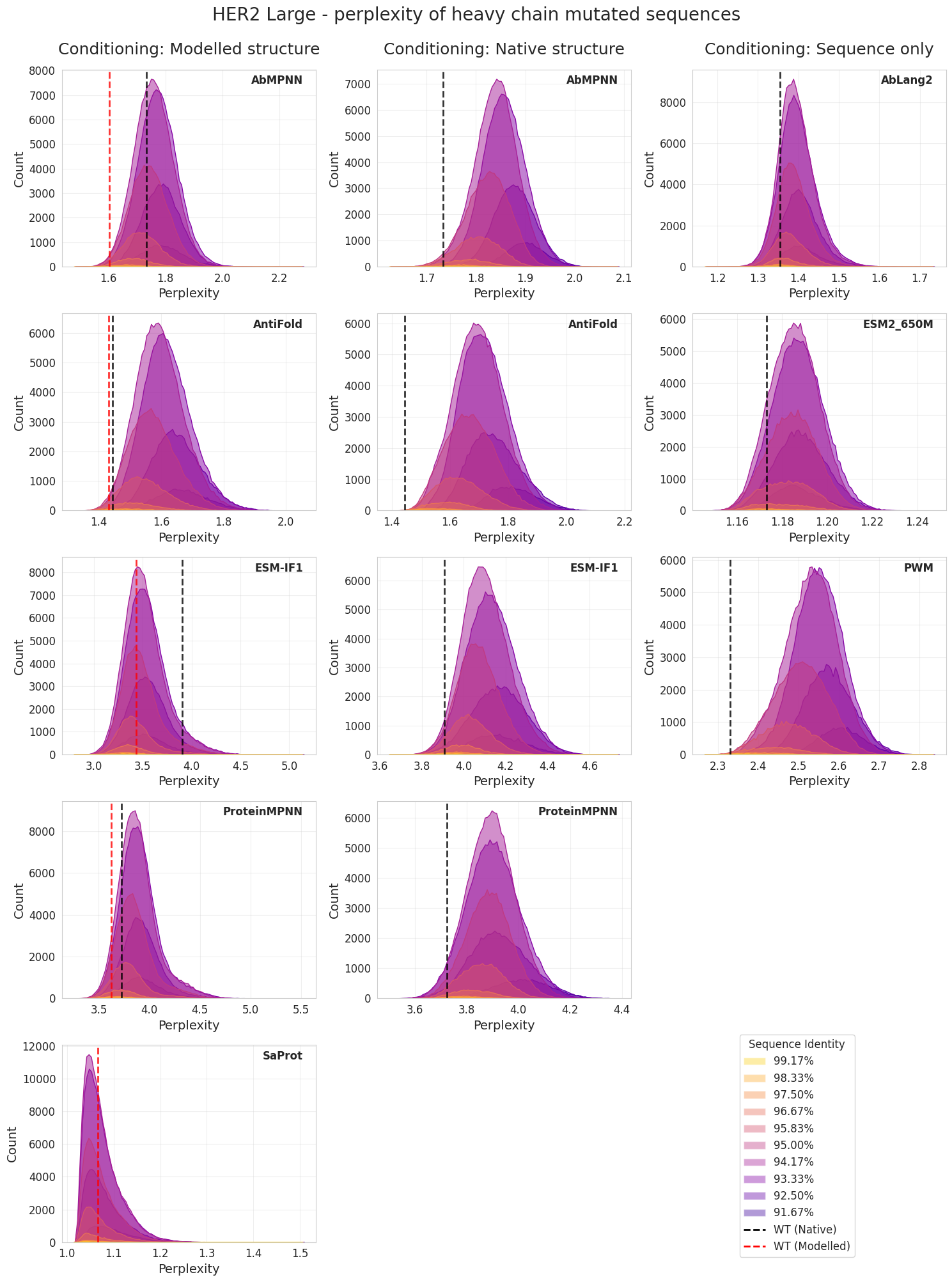


**Supplementary Figure 29: Perplexity distributions for Trastuzumab mutants over mutant’s sequence identity.**

All benchmarked models seem to favor higher sequence identity mutants, however highly unbalanced data in this regard lowers this finding significance.


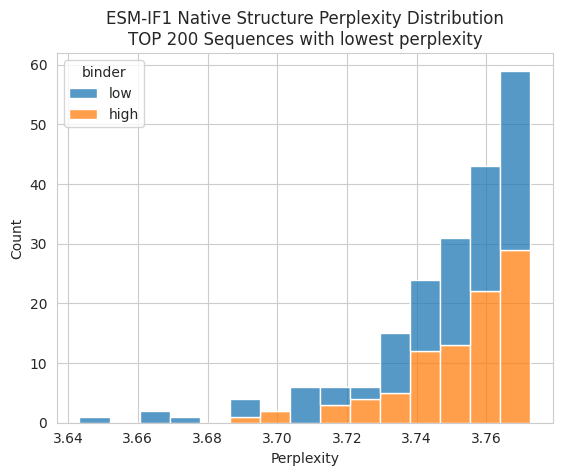


**Supplementary Figure 30: ESM-IF1 Top N outlier analysis**

From all benchmarked models, ESM-IF1 conditioned on native structure stands out as an outlier, due to many low binders scored with low perplexity values. Global distribution histograms hide those outliers.


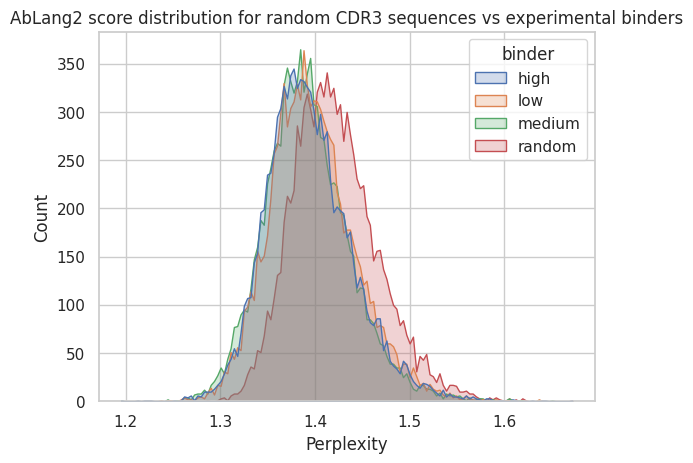


**Supplementary Figure 31: AbLang2 scores on high / medium / low and random binders.** AbLang2 shows the ability to distinguish between experimental and random Trastuzumab sequences.
